# Developing the UK’s capability in behavioural research: Identifying strengths, challenges and strategies through cross-sector workshops

**DOI:** 10.1371/journal.pone.0357909

**Published:** 2026-09-10

**Authors:** Guanyu Yang, Katherine R. K. Saunders, Niamh Hart, Joanna Davan Wetton, Nia Coupe, Isabelle Olson, Lucy Porter, Sharon Cox, Jo Hart, Susan Michie

**Affiliations:** 1 Centre for Behaviour Change, University College London, London, United Kingdom; 2 Usher Institute, University of Edinburgh, Edinburgh, United Kingdom; 3 Division of Medical Education, University of Manchester, Manchester, United Kingdom; 4 Department of Behavioural Science and Health, University College London, London, United Kingdom; University of Oxford Nuffield Department of Clinical Medicine: University of Oxford Nuffield Department of Medicine, UNITED KINGDOM OF GREAT BRITAIN AND NORTHERN IRELAND

## Abstract

Behavioural research shapes policy across multiple sectors; building strong capability for researchers and research users is essential to maximise its impact. This study aimed to identify strengths, barriers, and opportunities for improvement among those conducting or using behavioural research across UK sectors. We conducted five qualitative workshops, with 58 participants from academia, government, public and third sectors, and the private sector. Using framework analysis, growing visibility and successful embedding of behavioural research in some organisations were identified as key strengths. However, barriers included public misconceptions, limited funding and capacity, difficulties in research translation, siloed working, inconsistent training provision, and insufficient leadership support. Participants proposed actionable strategies, including making behavioural research more accessible, reforming funding structures, and enhancing cross-sector collaboration and training. Our findings highlight the need for sustained investment and stronger collaboration to better align behavioural research with public and policy priorities, address sector-specific challenges, and build on existing strengths.

## Introduction

Human behaviour is central to addressing major societal challenges in health, sustainability, education, crime and justice, social welfare, engagement with digital technology and artificial intelligence, and beyond. High-quality behavioural research has the potential to significantly strengthen policy and practice in these areas [1–5]. Behavioural research benefits from large-scale collaboration across disciplines and sectors, and explicit links between basic research, applied research, policy and practice [6–8]. However, the spread of behavioural research across many disciplines and sectors also means that behavioural evidence, theory and research methods can be fragmented, weakening the field and limiting its potential to be useful and impactful [8]. Improvements are also needed in scaling up interventions [9–11], safeguarding the ethical use of behavioural science [12–15], and accessing more comprehensive, diverse and high-quality data sources [5,16,17].

We currently lack a comprehensive picture of how well-equipped UK-based organisations (including academic institutions, governmental departments and teams, and private, public and third sector companies or teams) are (i) to conduct high-quality behavioural research to meet identified needs in policy and practice, and (ii) translate it into impactful outcomes. These organisations usually operate in silos with limited learnings from each other. This study formed part of a comprehensive assessment of UK behavioural research capability conducted by Behavioural Research UK (BR-UK) [18,19]. BR-UK is an ESRC-funded leadership hub within a wider UKRI programme to build national capability for behavioural research, with a mission to conduct interdisciplinary research addressing societal challenges.

Building on a documentary review of national and international strategy documents for advancing behavioural research capability [15], organisational network mapping [20] and a UK-wide survey [21], the workshops reported in this study represent the next phase in BR-UK’s capability building programme of work. It aimed to provide essential qualitative insights into sector-specific challenges and opportunities for strengthening behavioural research capability. In investigating capability, we adopted a broad definition inspired by the capability approach [22]: “the opportunities and conditions that enable people, groups, and institutions, whether they are researchers doing behavioural research or users applying it, to carry out and meaningfully engage with research”. This extends beyond access to resources such as funding and time to encompass the autonomy, expertise, and supportive culture required to translate those resources effectively into impactful contributions.

Recognising the rapid development of behavioural research in recent decades, scholars have reflected on various aspects of research capabilities via narrative review, qualitative, quantitative and mixed-methods designs [3,23–26]. This literature has documented progress and capability challenges such as funding constraints [24,25] and translation difficulties [27], but each study offers only a partial perspective. Most studies have concentrated on single sectors or contexts, such as behavioural science units advising policymakers [24,25], health services [27], or academic psychology departments [28]. In addition, much of the research on capability building originates from the health community [23,27,29,30], even though behavioural research spans a wide range of academic and professional disciplines, and topic domains. Existing meta-research also concentrates on the producers of behavioural research (e.g., Lecouturier et al. [24]), giving limited voice to its users. While recent research has examined the collaborative work between researchers and public bodies, it is confined to behavioural experiments [26], rather than extended to the broad array of methods used in behavioural research (such as qualitative methods). A multi-sectoral, multi-disciplinary perspective that incorporates the views of both researchers and research users is therefore needed.

Based on the findings from BR-UK’s previous capability building research phases and gaps identified in the wider literature, this study used inputs from researchers, organisational leaders, funders, and training bodies across academia, government, private, public and third sector bodies seeking to strengthen behavioural research capability across the UK. By mapping strengths, barriers and strategies for improvement across the sectors outlined above, we aimed to provide these stakeholders with i) evidence for strategic decision-making and organisational improvement through outlining barriers and enablers and ii) actionable, participant-generated strategies for strengthening their production and use of behavioural research, and for coordinating behavioural research across and within teams, organisations and sectors.

Building on previous phases of the capability scoping study, as described above, we aimed to answer:

1. What are the current strengths of existing behavioural research, including its impact on policy and practice in academia, government, private, public and third sector organisations?
2. What are the barriers to and missed opportunities in conducting and using behavioural research in academia, government, private, public and third sector organisations?
3. In what ways (through what potential strategies) can research capability be strengthened to facilitate more, better quality behavioural research that meets the needs of both researchers and research users in academia, government, private, public and third sector organisations?

## Methods

### Study design

We conducted five online workshops with UK behavioural researchers and research users across different sectors (Protocol: https://osf.io/r2fhv/overview). Workshops are a well-established research method for gathering qualitative data from diverse stakeholders, encouraging them to collaboratively generate strategic insights through group creativity and deliberation [31–33]. Although traditionally conducted face-to-face, the shift to online formats post-COVID-19 has provided a convenient, flexible and inclusive option for convening stakeholders across geographic boundaries [34–37]. This study received ethical approval from the UCL Research Ethics Committee (27193/001) and was reported in accordance with the Standards for Reporting Qualitative Research (SRQR) guidelines (See S1 File for checklist) [38].

### Participant recruitment and sampling

#### Recruitment.

We organised workshops by sector; academia, government, public and third sector, and small and medium-sized enterprises (SMEs), to capture sector-specific perspectives on behavioural research capability. We primarily identified participants from the pool of respondents to the BR-UK Cross-Sector Survey who had consented to follow-up contact [21]. If potential participants did not clearly fall into one sector, we allocated them to a workshop based on which was most aligned with their role. Government participants were invited via a cross-government behavioural science network. Participants were offered a voucher for their participation at a rate of £10 per hour up to £30. Government participants were not permitted to take payment. All participants were offered support funds for attendance/access costs. Participants were recruited between 15/10/2024 and 06/12/2024.

To be eligible for inclusion, participants were required to (1) meet the definition of a behavioural researcher or behavioural research user as described previously [39]; (2) work in a relevant organisation, department, or on topics relevant to the focus of the workshop; and (3) work in the UK. We excluded co-investigators or researchers within our own research consortium (BR-UK), as well as start-up or scale-up employees, since workshops tailored for this latter group were conducted in a separate study by a partner organisation [40].

#### Sampling.

We used purposive sampling to ensure representation across research topics, seniority levels, geographical locations, and personal characteristics. We held a final “cross-sector” workshop to convene voices missing or underrepresented in the previous four workshops, as observed by the research team and identified by previous workshop participants via the Equality, Diversity, Inclusion and Intersectionality (EDII) follow-up questionnaire.

### Workshop procedure

We conducted five online workshops (2.5 hours each) between November and December 2024 on Microsoft Teams: academia (n = 14), public/third sector (n = 13), SMEs (n = 9), government (n = 9), and cross-sector (n = 13). Due to low response levels from larger private sector organisations, participants in this group were invited to the cross-sector workshop. All participants provided written informed consent. We implemented measures to ensure inclusive participation [41], including offering access funds, holding sessions during core working hours, and enabling multiple forms of participation (e.g., chat and microphone).

Participants attended an opening plenary session before being divided into 2–3 breakout rooms per workshop, with 11 breakout rooms in total, each with a facilitator and note-taker. Facilitators and note-takers had experience in qualitative research methods and received briefing on the study protocol. Workshops followed a structured format with three facilitated group discussions in breakout rooms: (1) current strengths and examples of successes in behavioural research (approx. 20 minutes); (2) challenges and missed opportunities (approx. 20 minutes); and (3) strategies for capability improvement (approx. 40 minutes). Discussions were conducted under Chatham House Rule, meaning that contributions could be reported but were not attributed to individuals or organisations [42].

### Data collection

The workshops were recorded and transcribed, with detailed notes taken during discussions. Notes were anonymised and compiled as the primary data source for timely and efficient analysis considering the large amounts of parallel discussion data, with transcripts used to verify and illustrate key points as needed. Workshop materials to guide discussions and anonymised facilitator notes are available on Open Science Framework (See Data Availability Statement), with more detailed workshop procedure available in S2 File (Appendix A).

### Data analysis

Workshop data were analysed using framework analysis [43], a systematic method suitable for multi-participant workshop data where researchers can compare perspectives both within and across cases. As an iterative approach, it involved familiarising with data, coding, developing initial frameworks, charting data into matrices, and interpretation. We produced a matrix output with cases (breakout rooms in this study) as rows, and themes as columns, enabling us to examine patterns across sectors while maintaining the ability to trace findings back to specific workshop contexts [44,45]. Each matrix is referred to as a “framework”.

#### Familiarisation and initial coding.

The core analysis team of three researchers (KS, GY, NH) familiarised themselves with the data. The analytical approach was piloted with a subset of notes from three selected breakout rooms, which the three researchers first coded independently before comparing and calibrating their coding with a fourth researcher (NC). Codes agreed in this pilot phase formed a shared initial coding frame; disagreements were resolved through consensus discussion, and consistency was maintained through frequent team meetings throughout the analysis. It became clear that participants discussed strengths, barriers and strategies across all three breakout room activities and developed conversations around key topics of interest to them, which were important to represent in the framework. Therefore, indexing and charting data strictly according to the key research questions (strengths, barriers and strategies) would not capture nuanced discussions and emergent themes, and we therefore adapted our approach.

#### Indexing.

Taking a more inductive approach, the core analysis team iteratively coded the remainder of the notes, using agreed codes from the initial coding phase and creating new ones as necessary. These were iteratively reviewed and refined, resulting in 141 codes.

#### Charting.

The indexed notes were structured into framework matrices using Microsoft Excel. The codes were grouped into 23 umbrella themes based on conceptual similarity and then were further organised into eight separate framework matrices based on thematic coherence. Areas of ambiguity or overlap between frameworks were reviewed and resolved through team discussion. The full coding tree, showing the correspondence between the codes, the key themes, and the eight final frameworks, is provided in S5 File (Appendix D).

#### Interpretation.

With data organised across eight thematic frameworks, which are presented in the results section of this paper, we analysed patterns both within and across sectors in line with our research questions. We identified challenges and strengths across and within sectors, and examined how proposed strategies varied, or were consistent, by sector. We narratively summarised each framework and tabulated key strategies.

Although strategy was the focus of the final breakout session of each workshop, where participants were directly asked to explore strategies or solutions that would improve or strengthen behavioural research in their organisation, strategies and solutions were spontaneously discussed throughout the workshop sessions. Strategy data were mapped onto the same thematic frameworks as the strengths and challenges data, so that each framework contained its own set of participant-proposed strategies. From each framework, the research team selected the most salient strategies through team discussion; selection was guided by the level of discussion, agreement and support in workshops, and corroboration from the strengths and challenges data. For reporting, the selected strategies were then consolidated into 10 strategies by combining those that recurred across multiple frameworks. Each of the 10 strategies comprises a set of concrete operational actions proposed by participants. Each strategy was mapped back onto the framework(s) from which it originated and onto the actor groups, that is, the individuals and organisations with potential agency and responsibility to implement it.

Workshop participants were invited to review and validate the initial framework draft in July 2025 to ensure accuracy of the interpretation. The consolidated strategy table, developed later, was shared with the work package group for expert input and confirmation.

## Results

### Strengths and barriers to building behavioural research capability

Participants identified a range of strengths and barriers to building behavioural research capability across academic, government, private, public and third sectors across the UK. These eight core frameworks were derived through the analysis: (1) public perceptions and understanding, (2) communication and translation, (3) collaboration, networking and engagement, (4) research practice and methodology, (5) behavioural research impact, (6) funding, resources, capacity and accessibility, (7) training and career development, and (8) leadership, embedding and prioritisation. Each is represented in a separate framework table for clarity, although some frameworks are connected and overlapping in nature. A summary of the strengths and barriers across each is shown in Table 1 and Table 2 and subsequent narrative summaries, with complete frameworks available in S3 File (Appendix B).

**Table 1:** Summary of strengths in behavioural research identified across eight frameworks.

Framework	Strengths	Workshops^1^
**1. Public perceptions and understanding**	Growing visibility and adoption	All workshops
	Use of common language	All workshops
	Widely used theories and frameworks	Academia, Government, Cross-sector
	Solid evidence base	Government, SMEs, Public & third sector, Cross-sector
**2. Communication and translation**	Successful cases of communication and translation	Public & third sector, SMEs, Cross-sector
**3. Collaboration, networking, and engagement**	Effective collaborative and knowledge-sharing mechanisms	All workshops
**4. Research practice and methodology**	Ability to tailor to contexts	Academia, Public & third sector, Cross-sector
	Progress in applying systems thinking	Academia, Government
	Momentum in methodological innovation, ethical awareness and standards	Government, SMEs, Cross-sector
**5. Behavioural research impact**	Demonstrated value in policy systems; shift to real-world application	Government, SMEs
**6. Funding, resources, capacity and accessibility**	None identified	—
**7. Training and career development**	Various established training provided across sectors	SMEs, Cross-sector
	New generation of behavioural researchers with growing career options	Public & third sector, SMEs, Cross-sector
**8. Leadership, embedding and prioritisation**	Improving leadership and client appetite	Government, Cross-sector
	Successful cases of embedding behavioural research	Government, Public & third sector, SMEs, Cross-sector

Note: ^1^ The Workshops column denotes the sector-specific workshops where the respective points of strengths were identified.

**Table 2:** Summary of barriers in behavioural research identified across eight frameworks.

Framework	Barriers	Workshops^1^
**1. Public perceptions and understanding**	Oversimplification as just “nudging”	Government, SMEs
	Seen as “social engineering” or manipulation	Government, Public & third sector
	Confusion around terminologies and technical processes	Public & third sector, SMEs, Cross-sector
	Misconception that everyone understands human behaviour	SMEs, Cross-sector
**2. Communication and translation**	Difficulty translating research outputs	All workshops
	Difficulty bringing research into practice	Academia, Public & third sector, SMEs, Cross-sector
**3. Collaboration, networking, and engagement**	Academics working in silos	Academia, Public & third sector, SMEs
	Weak connections across sectors	Government, Public & third sector, SMEs
	Lack of trust and understanding	Public & third sector, SMEs, Cross-sector
	Low visibility of ongoing work	Public & third sector, SMEs
	Difficulty attending networking events	Academia, SMEs, Cross-sector
	Difficulty managing expectations	Academia, Public & third sector, SMEs
**4. Research practice and methodology**	Difficulty tailoring to complex contexts	All workshops
	Heavy focus on individual behaviour over context	Academia, Government, Public & third sector
	Continuing inconsistent methodological and ethical standards	Academia, Government, Public & third sector
	Difficulty capturing outcomes	Academia, Government, Public & third sector, Cross-sector
	Methodological ideals vs real-world constraints	Academia, SMEs, Cross-sector
	Academic norms limiting impactful practice	Public & third sector, SMEs
	Undervaluing of qualitative research	Cross-sector
	Concerns over data quality and integrity	Government, Cross-sector
**5. Behavioural research impact**	Difficulty influencing policymakers	Academia, Government
	Constrained capacity and time for outcomes	Government, Public & third sector, SMEs
	Difficulty evaluating policy impact	Government, Public & third sector, Cross-sector
	Knock-on effects in other policy domains	Academia, Government
	Demand for quick results vs long-term impact	Government, Public & third sector
	Short-term funding cycles	Public & third sector, SMEs
**6. Funding, resources, capacity and accessibility**	Lack of long-term, stable funding	Academia, Government, Public & third sector, SMEs
	Limited time, resource, and expert knowledge	Academia, Government, Public & third sector, Cross-sector
	Difficulty accessing academic journal articles	Government
**7. Training and career development**	Difficulty identifying suitable training	Government, Cross-sector
	Limited resources for training	Government, Public & third sector
	Difficulty breaking into the industry and government	Academia, Government, SMEs
	Difficulty defining researcher identity	Public & third sector, Cross-sector
**8. Leadership, embedding and prioritisation**	Limited understanding among senior leaders and stakeholders	Public & third sector, Cross-sector
	Limited behavioural research integration in research process	Academia, Government, Public & third sector, Cross-sector
	Hard to position behavioural research within organisations without leadership champions	SMEs, Cross-sector
	Lack of interest from decision-makers	Academia, Public & third sector

Note: ^1^ The Workshops column denotes the sector-specific workshops where the respective points of barriers were identified.

#### Framework 1: Public perceptions and understanding.

The four key themes within this framework are: 1) characteristics of behavioural research, 2) strong interest in behavioural research, 3) increased visibility and adoption of behavioural research, and 4) misconceptions and related challenges. Consensus across sectors shows behavioural research has achieved markedly increased and improved visibility and adoption, expanding beyond health and environmental research areas. Other key strengths mentioned include frameworks like COM-B [46] providing a common language for collaboration and a robust, highly regarded evidence base (particularly in health applications, e.g., smoking cessation) delivering scientific credibility that appeals to many audiences. However, fundamental misconceptions were found to persist across sectors: stakeholders may oversimplify behavioural research as “nudging”, dismiss research or interventions as manipulation, or confuse terminology. Participants also described a pervasive belief that everyone inherently understands human behaviour, which they felt led to resistance to evidence-based approaches.

#### Framework 2: Communication and translation.

The two key themes in this framework are: 1) approaches to and importance of research communication and translation and 2) challenges in communication, translation and implementation. While we recognise that communication and translation are distinct concepts, workshop participants discussed and explored them as intrinsically related concepts and therefore they have been analysed and reported together. Examples of communication and translation successes included the use of evidence-based advisory groups throughout a project on organ donation in Northern Ireland, the development of a sexual health website for young people that resulted in increased bookings for sexual health screenings, and charities optimising email language to focus on enabling recipients rather than appearing manipulative. A major challenge was translating complex behavioural research in an accessible way without compromising scientific rigour, with cross-sector participants recognising difficulties in explaining methods to external audiences. There were also significant time constraints for reading and digesting long reports, with participants calling for succinct summaries. Government participants noted their continuing struggle to translate policy problems into behavioural research and vice versa, making it challenging to meet the specific needs of policy colleagues.

#### Framework 3: Collaboration, networking, and engagement.

The three key themes in this framework are: 1) cross-sector collaboration, 2) experience sharing, networking and community building, and 3) stakeholder engagement. There were successes in the development of cross-sector collaboration models: for instance, the Competition and Markets Authority’s adoption of choice architecture language enabled lawyers and economists to work together effectively. Experience-sharing systems brought up during the discussion include the Welsh government’s community of practice and cross-government behavioural insights networks, with SME participants describing a “strong, tight-knit global community” that positions the UK as optimal for behavioural research. One barrier identified is researchers working in silos, with government and SME discussions further highlighting the disconnect between academia, the third sector, and government. Mismatches in stakeholder expectations were reported across workshops, with demands for quick results and large-scale outcomes conflicting with the small changes typical of effective behavioural interventions. Experience sharing was found to be limited by a lack of visibility of ongoing, external work and networking difficulties caused by costly, often London-centric events.

#### Framework 4: Research practice and methodology.

The two key themes within this framework are: 1) tailoring to contexts and systems thinking and 2) research best practices. Participants highlighted the ability to tailor behavioural research to contexts as a key strength. Cross-sector participants emphasised how flexible approaches enabled safer research engagement, such as remote participation for vulnerable populations, while public and third sector participants shared examples of adapting behavioural frameworks through bespoke internal training. SME participants also valued the strong ethical practices underpinned by the UK’s robust regulatory frameworks (i.e., the Financial Conduct Authority and the Competition and Markets Authority), which provide credibility. One key methodological challenge identified is a lack of systems thinking: government participants warned of the potential unintended knock-on effects across sectors and populations when behavioural research or interventions focus solely on individual behaviour, without consideration of context. Cross-sector participants also noted gaps between behavioural science training and practical application, while SME participants highlighted tensions between the need for real-world testing and client demands for controlled evidence upfront.

#### Framework 5: Behavioural research impact.

The two key themes in this framework are: 1) considering impact in behavioural research and 2) behavioural research and policy. In terms of strengths, government participants highlighted the value of behavioural research in strengthening policymaking systems. Specifically, they noted that behavioural research enhances accountability, helps to clarify underlying problems, and prompts policymakers to consider the unintended consequences of proposed actions. Government participants also cited the successful application of insights from behavioural research during the COVID-19 pandemic as a good example of evidence-based policymaking. Regarding barriers, academic participants reflected on their difficulties in influencing decision-makers, which was supported by government participants who emphasised that political realities may prevent implementation even when evidence is robust, thus limiting impact. Concerns across government and public and third sector groups also include demands for short-term results despite the long-term and non-linear nature of behaviour change.

#### Framework 6: Funding, resources, capacity and accessibility.

The four key themes in this framework are: 1) accessibility of behavioural research and related resources, 2) central resource bank strategies, 3) issue of funding and related challenges, and 4) limited capacity and resources. No strengths were identified, with the discussion concentrating on barriers. Participants from all sectors cited lack of long-term stable funding as a key barrier and felt that funders do not understand the costs involved in behavioural research and that impact can be difficult to demonstrate in short-term funding cycles. Both SME and academic participants desired more funding for longer-term collaborations. Government and cross-sector workshops noted behavioural research is often cut first during budget reductions, despite being core to the research strategy. Government teams also often lost expertise through staff turnover, leading to outsourcing of research. Lack of accessibility to resources was seen as a significant barrier to conducting and using behavioural research across sectors, for example, government participants noted that they are unable to access and make use of work contained within academic journals.

#### Framework 7: Training and career development.

The three key themes in this framework are: 1) importance of, need for and barriers to training, 2) examples of and potential approaches to research training, and 3) career routes and researcher confidence. Participants shared examples of effective training approaches, both in terms of providing or attending behavioural research training or using behavioural research techniques in delivering training. Examples discussed from academia and the public and third sector respectively include i) using the Behaviour Change Wheel framework and COM-B model as teaching tools and ii) developing in-house training on social sciences and behavioural science frameworks. Participants also reported that behavioural research had increasing career options, with SME participants seeing behavioural science as an outlet for psychology graduates. Challenges include difficulty identifying opportunities outside academia, gaps in high-quality, consistent training provision, and limited external training budgets. Career barriers outside academia include difficulties in breaking into industry and low behavioural research presence in government, which participants said made it harder to attract quality researchers. In addition, public and third sector participants questioned what qualifications or experiences are required to claim the title of ‘behavioural researcher’. Some described feeling fraudulent identifying as behavioural researchers without formal training.

#### Framework 8: Leadership, embedding and prioritisation.

The three themes in this framework were 1) importance of leadership and ownership of engagement, i.e., buy-in, 2) embedding behavioural research into organisations and structures, and 3) prioritisation of behavioural research. Strengths identified include an increased appetite in government for behavioural research despite variations between governments and individual ministers, and the growth of behavioural science as a profession. Elsewhere, it was noted that behavioural research approaches, such as the COM-B model, have long been used (in markets and commercial research, for example) but not cited as such, and that these techniques are now starting to be more clearly embedded. Challenges include limited impact due to behavioural research being considered at the last minute, clients feeling excited about behavioural research initially but then failing to translate this into longer-term engagement, and requiring demonstration of cost savings to senior leaders who lack understanding of time and resource requirements. Public and third sector participants felt that charismatic leadership was needed to elevate the status of behavioural research.

### Strategies to strengthen research capability

Across the workshops, participants suggested and discussed strategies by which behavioural research capability could be improved in their sector and/or area of expertise. Strategy ideation was the focus of the final third breakout session of each workshop, but participants also raised and discussed strategies spontaneously throughout the workshops. The strategies centre around 10 overarching approaches to improving capability: 1) Creating accessible communication resources, 2) Establishing a central repository/resource bank, 3) Embedding behavioural science into policy and research processes, 4) Building multi-level training programmes, 5) Reforming funding structures, 6) Establishing behavioural research leadership and champions, 7) Strengthening stakeholder and public engagement, 8) Creating transdisciplinary roles and networks, 9) Shifting to systems-level approaches, and 10) Establishing quality standards and accessible guidance. Each strategy comprises a set of concrete operational actions proposed by participants; the strategies are thematic and are not presented in ranked order. Table 3 presents these alongside one illustrative quote per strategy, the corresponding frameworks, and the key ‘actors’ (individual and organisational) with potential agency to implement them. The example quotes presented in this table were extracted from the workshop transcripts for the purpose of illustration and to provide contextual detail. A complete list of strategies is available in S4 File (Appendix C).

**Table 3:** Key strategies identified in workshops for capability building in behavioural research.

Key strategies	Example participant quotes	Corresponding framework(s)	Key actors
**1. Create accessible communication resources** • Provide plain language summaries alongside technical details • Use bite-sized case studies with clear methodology • Use multiple channels (social media, newsletters, podcasts, and school education) • Use storytelling and narratives	*“I know my colleagues wouldn’t have time to look at that [research papers], so seeing kind of bite size [...] case studies and then possibly kind of webinars explaining those things in more detail just makes it that much more kind of accessible and relatable.”* (Public & Third sector)	Public perceptions and understanding; Communication and translation; Collaboration, networking, and engagement; Training and career development	Researchers; Research community/networks
2. **Establish a central repository/resource bank** • Create searchable databases of interventions and case studies • Include failings, learnings, null results and replications • Provide AI-powered search functions to find tailored cases • Include job boards and career opportunities	*“It would have certainly been useful to us at the beginning of our programme - a repository of case studies on previous behavioural science programmes. How they were implemented, what worked, what didn’t.”* (Government)	Research practice and methodology; Behavioural research impact; Funding, resources, capacity and accessibility; Training and career development	Researchers; Research community/networks; Universities/research institutions
**3. Embed behavioural science into policy and research processes** • Add behavioural science to policy development checklists • Position behavioural research earlier in decision-making • Invest in organisational-level capability, including formal facilitating roles • Create secondment opportunities	*“In government departments, […] there’s a checklist which currently asks ‘what is the science advice in relation to this policy and development?’. And I think to have a separate box or checklist which says ‘is there a behavioural science advice?’ [...] is a system change that at least ensures that the issues will be considered.”* (Government)	Research practice and methodology; Behavioural research impact; Leadership, embedding and prioritisation; Training and career development	Universities/research institutions; National/local governments; International organisations; Industry partners; Research user organisations
**4. Build multi-level training programmes** • Provide training at different levels (introduction to advanced) • Include mentorship and implementation support • Build a searchable list of training opportunities	*“It would be really helpful to have training and guidance that’s kind of tailored to different audiences [...] A lot of the people[…] in public health roles or health and social care [...] do want to kind of draw on people research and undertake it themselves, but often are scared by [...] the level of robustness required.”* (Public & Third sector)	Research practice and methodology; Funding, resources, capacity and accessibility; Training and career development; Leadership, embedding and prioritisation	Universities/research institutions; Research user organisations
**5. Reform funding structures** • Create funder collectives for larger projects • Target local authority partnerships • Support cross-sector collaboration • Define impact and success realistically when working with funders	*“People are kind of our resource. They’re our Large Hadron Collider and they are expensive.”* (Academia)	Behavioural research impact; Funding, resources, capacity and accessibility	Research funders
**6. Establish behavioural research leadership and champions** • Create formal supportive leadership positions within organisations • Identify and support organisational champions • Train leaders on strategic planning and realistic expectations	*“I’d like to see changes [...] in the higher, higher leadership. There just seems to be not a clear understanding of what behavioural science is and how it can help so oftentimes [...] we get asked to contribute to things and we’re thought of as like a magic bullet. And that way if you get us involved, we’ll fix it [...] it’s not how we work at all.”* (Public & Third sector)	Public perceptions and understanding; Leadership, embedding and prioritisation	Researchers; Research users/practitioners; Research community/networks; Universities/research institutions; Research funders; National/local governments; International organisations; Industry partners; Research user organisations; Publishers
**7. Strengthen stakeholder and public engagement** • Co-design solutions with affected populations and advisory groups • Educate clients and stakeholders about research processes • Ensure local and national government involvement in research for policy impact	*“There’s a need to have a conversation around the different stakeholders [...] for example, another sector might see a nudge differently from sectors such as marketing, for example.”* (SMEs)	Collaboration, networking, and engagement; Behavioural research impact	Researchers; Research users/practitioners; National/local governments; International organisations; Industry partners; Research user organisations
**8. Create transdisciplinary roles and networks** • Bridge gaps between sectors and disciplines • Facilitate cross-sector learning and collaboration • Establish networking mechanisms (forums, conferences, mentorship) • Ensure geographic accessibility through online and regional events	*“More opportunities to bridge the gap between the fields would be great... also just improving networks for collaboration between like researchers, NGOs.”* (Public & Third sector)	Collaboration, networking, and engagement; Research practice and methodology	Research community/networks; Universities/research institutions; International organisations; Industry partners; Research user organisations
**9. Shift to systems-level approaches** • Move beyond individual behaviour models • Use systems mapping as a foundation • Focus on long-term and sustainable change	*“Now we’re really interested in how you apply systems thinking in particular to creating sustainable behaviour change, because we recognise there’s a need for that in particular.”* (Academia)	Research practice and methodology; Behavioural research impact	Researchers; Research users/practitioners; Research community/networks; Industry partners; Research user organisations
**10. Establish quality standards and accessible guidance** • Clear definitions and terminology • Methodological standards • Sector-specific guidance	*“It would be good to have sort of something to lean on external to the organisation […] like the NICE guidelines, you know, shape how healthcare happens and they’re referred to. I almost visualise something a bit like that, but a behavioural science lens.”* (Public & Third sector)	Public perceptions and understanding; Research practice and methodology; Funding, resources, capacity and accessibility	Research community/networks; Universities/research institutions; International organisations; Publishers

Note: “industry partners” refers to partners co-producing research; “research user organisations” refers to public, private and third sector organisations applying research.

## Discussion

### Overview of key findings

This study identified current strengths, barriers to and missed opportunities in conducting and using behavioural research across sectors in the UK, and potential strategies which can strengthen research capability across sectors. Strengths included cross-sector increased visibility and institutional presence across the UK, perceived credibility rooted in a growing robust evidence base (particularly in health), successful applications in policy and practice such as during COVID-19, and strong collaboration and methodological innovation. Concerning the barriers and missed opportunities, the workshops revealed persistent misconceptions about behavioural research, siloed working, and challenges in translating evidence into practice. Cross-sector issues such as short-term funding, capacity limitations, and a lack of training were consistently reported, in addition to institutional resistance and a lack of leadership to sustain long-term behavioural research practices. Participants proposed 10 priority strategy areas, including improving behavioural research accessibility and establishing central resources, structural embedment of behavioural research in research processes, and improving leadership engagement. The cross-sector workshop produced findings that were largely in agreement with our sector-specific workshops, emphasising the cross-sector nature of many of the strengths and challenges and the mutual need for collaboration and resource sharing.

### Comparison with relevant evidence

This study found that behavioural research in the UK has gained significant public visibility and understanding over the last few years, which mirrors the global pattern [25,47]. It has also taken root in an increasing number of UK organisations with dedicated teams being set up [3]. The study identified several successes in translating research into practice and existing networking mechanisms, reflecting strong collaborative relationships and knowledge exchange between researchers and stakeholders [24]. Behavioural research was seen as having an impact on policymaking, for instance, during the COVID-19 pandemic, and in private-sector applications, consistent with other findings [3,24,48,49]. These successes raise the question of how the rapid routes for behavioural evidence into government decision-making seen during the COVID-19 pandemic could be sustained and embedded in future crisis responses.

No strengths were mentioned regarding funding, resources, or capacity, suggesting a lack of supportive resources and capacity in behavioural research, which is reflected also in other evidence [15,27,28]. Gaps were identified between research and practical application, with research silos and limited cross-sector connections constraining collaboration. This research-practice gap has been identified previously across several contexts [15,24–26]. For example, policymakers often demand quick, conclusive results while researchers require time for rigorous evaluation; the problems of this short-term approach have been widely discussed (see Sanders et al. [3]). Another finding that resonated with previous evidence was participants’ call for systems-level approaches to align with growing international recognition that individual behaviour change interventions face inherent limitations when structural constraints are present [15,50].

The 10 strategies that emerged from workshops provide targeted responses to the barriers identified above. Creating accessible communication resources and central repositories directly addresses both the persistent translation gaps between research and practice [24,26], as well as public misconceptions about behavioural research. The emphasis on embedding behavioural science into policy processes and establishing formal leadership positions and champions responds to the institutional resistance and marginalisation challenges that prevent sustained impact [15,21]. Multi-level training programmes can address the fragmented and inconsistent training provision and the identity crisis facing behavioural researchers [21]. Transdisciplinary networks and roles specifically address siloed working and limited cross-sector connections. Meanwhile, strengthened stakeholder engagement will help manage the mismatched expectations between researchers who require rigorous evaluation and policymakers and industrial clients who demand quick results, as found in previous studies [40]. What distinguishes these strategies from recommendations in prior work is their specificity regarding implementation mechanisms: participants proposed concrete tools, such as formal facilitating roles, specific funding collective models and behavioural science checklists, to supplement abstract recommendations with more actionable steps.

### Strengths and limitations

This study benefitted from using workshops for data collection and the inductive approach of framework analysis, which allowed for easy stratification and synthesis of data from each workshop and across sectors and themes. The workshop guide was designed to generate actionable insights, resulting in the collection of concrete strategies for improving behavioural research to inform capability-building efforts. We gathered participants from multiple sectors, providing a rich mix of perspectives and diverse insights. The piloting of the analytical approach, using a purposefully selected small number of workshop notes and frequent meetings for calibration, ensured the team used a harmonised approach in coding the notes. Holding a final workshop for missing voices helped to capture additional perspectives. Workshops as a data collection method can create challenges through group dynamics, including dominant voices, underrepresentation of dissenting views, social desirability and conformity pressures. This was addressed through i) the use of experienced and well-briefed facilitators, ii) agreeing at the outset that contributions would be treated as confidential to the workshop and not attributed to individuals or organisations, iii) encouraging use of the chat function, and iv) in the analysis process, triangulating topics of discussion across transcripts. The analytical approach chosen also enabled us to show that the cross-sector workshop essentially confirms rather than contradicts sector-specific findings. While some topics were unique to specific sectors, core issues such as funding, translation, and collaboration appeared consistently across workshops.

There are a few key limitations of this study. There was a risk of selection and self-selection bias as those already invested in increasing behavioural research capability may have agreed to participate, and the primary pool for invitees consisted of those who had already engaged with BR-UK in their initial survey, making them a particularly research-engaged group. Due to resource constraints, we primarily analysed workshop notes rather than verbatim transcripts, so there is the potential for some missed nuances of the discussions. We did not collect detailed information on the composition and representativeness of participants across sectors, making us unable to comment on the generalisability of our findings across sectors. Furthermore, the proposed strategies were not implemented or evaluated in this study, resulting in limited information regarding feasibility, acceptability, priority, cost, or potential impact. Finally, these findings describe behavioural research capability in a single national context. While many of the barriers raised, such as fragmented funding and gaps between research and practice, are consistent with international evidence [15,24,25,50], participants’ examples and strategies referred to UK institutions and government arrangements. The proposed strategies are therefore transferable in principle but would require adaptation to other funding systems and policy structures.

### Implications for policy and practice

Many of the strategies to improve behavioural research capability provide practical next steps for those wanting to support behavioural research and its use in their organisation in the UK. For example, government departments could benefit from making behavioural research roles in their civil services more visible and creating more opportunities for secondments for behavioural researchers into government, to facilitate better research-policy translation. Training bodies and research institutions could develop a central resource bank of high-quality behavioural research examples and training, along with sector-wide methodological standards and guidelines, particularly relating to data protection and AI use. More strategically, organisational leaders across sectors could consider embedding behavioural research into organisational decision-making processes and establishing formal champion roles to address the leadership gap, while research funders could usefully commit to increased, long-term funding for cross-sector multi-disciplinary collaboration. This would address the critical funding barriers identified across all sectors by establishing longer funding cycles and creating funder collectives for large-scale initiatives.

### Implications for research

Behavioural research should continue to prioritise a systems approach to ensure that it has the capability of being effective over the long term in addressing community and societal challenges. Behavioural research teams should consider including key stakeholders from the outset of projects and make efforts to effectively translate findings for their target audiences. Learnings from high-impact research case studies should be highlighted and shared in keeping with open-science principles, in order to improve practice and support the sharing of learnings outside of academia. Where behavioural research forms a component of a broader, multifaceted project, it should be built into the overall project strategy from the beginning. As a study that builds directly on BR-UK’s UK-wide capability survey [21], this workshop study adds qualitative depth to previously identified quantitative patterns. In-depth individual interviews could further enrich specific findings without the social complexities of a group workshop.

## Conclusion

This study provides a multi-sector examination of behavioural research capability across the UK, revealing a field with significant visibility and institutional recognition but facing challenges that threaten its current and sustained impact. The findings outline strategic priorities crucial for advancing behavioural research, and provide a comprehensive blueprint for coordinated action across stakeholder groups. Such action ranges from developing systems thinking capabilities among researchers to restructuring funding approaches for decision-makers. Only through coordinated investment in behavioural research infrastructure can fragmented efforts be transformed into cohesive, transparent, evidence-based practice with greater societal impact.

## Supporting information

S1 File Standards for Reporting Qualitative Research (SRQR) checklist. (PDF)

S2 File Appendix A: Workshop procedure. (DOCX)

S3 File Appendix B Framework tables. (DOCX)

S4 File Appendix C: List of detailed strategies from each framework and illustrative quotes. (DOCX)

S5 File Appendix D: Coding tree showing the correspondence between codes, key themes, and the eight frameworks. (DOCX)
