# Supplementary material for: Developing the UK’s capability in behavioural research: Identifying strengths, challenges and strategies through cross-sector workshops
Source: PLoS One. 2026 Sep 10;21(9):e0357909. doi: 10.1371/journal.pone.0357909 (PMC13561315; doi:10.1371/journal.pone.0357909)
Supplement: Supplementary file 2 [file pone.0357909.s002.docx]

## Appendix A: Workshop procedure

### A1. Number and duration of workshops

We conducted five workshops in November and December 2024, one with academics (n=2 breakout rooms, n=14 participants), one with the public and third sector (n=3 breakout rooms, n=13), one with small and medium enterprises (SMEs) in the private sector (n=2 breakout rooms, n=9 participants), one with government representatives (n=2 breakout rooms, n=9), and one cross-sector workshop (n=2 break out rooms, n=13 participants). We originally intended to hold two private sector workshops, one for SMEs and one for larger firms. However, too few participants from large firms signed up to participant in the workshops to warrant a separate session and they were instead invited to the cross-sector workshop. All workshops were conducted on Microsoft Teams. The workshop activities were highly structured, and each took place over 2 hours 30 minutes.

### A2. Format and facilitation

Each workshop was split into break out rooms. The number of break out rooms depended on the number of confirmed attendees and research team capacity for facilitation and note taking. Each break out room was allocated one facilitator and one note taker. One member of the research team remained available to support with tech issues, managing the breakout rooms and chair feedback sessions in the main room. Participants were informed that the discussions were held under the Chatham House Rules [1] and reminded of this again at the end of the workshop. In break out rooms, activity 1 involved a facilitated group discussion of current strengths in behavioural research and examples of successes, activity 2 involved a facilitated group discussion of current challenges in behavioural research and examples of missed opportunities, and activity 3 built on the first two activities with a facilitated group discussion on potential strategies for increasing UK capability in behavioural research. Materials used to guide each workshop discussions can be found in on [Open Science Framework](https://osf.io/yzjvb/overview?view_only=8cf33cf1a437487f9dbb180a6f60648e) (OSF). Anonymised facilitator notes from the workshops (i.e. the main primary data used for analysis) are shared on OSF.

### A3. Ethical considerations and consent

This study received ethical approval from the institutional Research Ethics Committee (ID number 27193/001). Participants provided informed consent to take part, anonymous notes were taken during the workshops and were provided with the opportunity to review the draft results to ensure accuracy of the researcher interpretation for two weeks in July 2025. Participants reviewed draft results for accuracy in July 2025.

### A4. Approach to Equality, Diversity, Inclusion and Intersectionality (EDII)

To ensure inclusivity, we held workshops during core working hours, offered an access fund, and shared materials in advance. We invited a diverse range of participants and collected anonymous EDII data to understand representation. Our approach was reviewed by the BR-UK EDII Group, and post-workshop feedback helped assess accessibility. Our workshops were held online with measures in place to support digital accessibility, for example, using platforms that meet [Web Content Accessibility Guidelines](https://www.gov.uk/service-manual/helping-people-to-use-your-service/understanding-wcag) (WCAG) standards, enabling multiple ways to participate (e.g., chat, microphone, captions), and inviting disclosure of access needs in advance to offer tailored support. Facilitators were briefed on accessibility practices, and a team member was available to resolve technical issues in each workshop. See the [study protocol](https://osf.io/yzjvb/overview?view_only=8cf33cf1a437487f9dbb180a6f60648e) for full details.

### A5. Data Collection

Workshops and break out rooms were recorded and auto-transcribed using Microsoft Teams, and note takers took detailed notes of discussions in breakout rooms. The notes from each note taker were then anonymised, compiled into a master file, which was used as the primary source of data for the framework analysis. These notes were complemented by the recordings and transcriptions where details were unclear. All research data were managed and stored safely according to our [study protocol](https://osf.io/yzjvb/overview?view_only=8cf33cf1a437487f9dbb180a6f60648e).

### Reference

1.	Chatham House. Chatham House Rule. n.d. Available: https://www.chathamhouse.org/about-us/chatham-house-rule
