# Supplementary material for: Developing the UK’s capability in behavioural research: Identifying strengths, challenges and strategies through cross-sector workshops
Source: PLoS One. 2026 Sep 10;21(9):e0357909. doi: 10.1371/journal.pone.0357909 (PMC13561315; doi:10.1371/journal.pone.0357909)
Supplement: Supplementary file 3 [file pone.0357909.s003.docx]

# Appendix B Framework tables

## Appendix B1

**Table B1. Framework 1: Public perceptions and understanding**

| **Breakout room** | **Characteristics of behavioural research** | **Strong interest in behavioural research** | **Increased visibility and adoption of behavioural research** | **Misconceptions and related challenges** |
| --- | --- | --- | --- | --- |
| Academic 1 | Behavioural science is valued in interdisciplinary research, particularly in the built environment, helping move beyond technical solutions to better understand people and design more sustainably. Behavioural research allows us to use its data in a co-productive way to include human elements and emotions, bringing data and people together. Behavioural science can bridge disciplines.  In behavioural research, there are behavioural labs that can test, measure and quantify interventions like nudges, allowing comparison of different approaches.  Behavioural science provides a common language and structured approach that helps change mindsets when communicating with government and reassures healthcare communities through frameworks like COM-B. This shared language benefits collaboration with software developers and enables meaningful inclusion of lived experiences. Standardisation in behavioural research includes using established language like COM-B diagnosis that reassures healthcare communities. Some kite mark of standardisation showing what’s (not) backed by science or evidence would help combat misinformation.  Behaviour is complex, and simplifying it remains a question. | The aggressive nature of marketing drives companies to seek behavioural analysis, with more companies hiring behavioural analysts because practitioners need the data. Some sectors now recognise and take behaviour change seriously, while others question what behaviour even is, creating challenges. Behavioural science is well-established in public health.  Social media discussions about behaviour change often lack scientific backing, potentially devaluing research. Areas like speech and language therapy (SALT) and physiotherapy still struggle with understanding behaviour change. | Some sectors are showing real recognition that behaviour change is legitimate and take it seriously.  Behaviour change needs to be more common and popular, with general awareness being an uphill challenge. | Misconceptions include oversimplification of behaviour and missing cultural interpretations. There is sometimes a perception when working with companies that behavioural scientists are constantly seeking evidence, which isn’t always appreciated. People on social media are discussing behaviour change without scientific backing can devalue the research.  While some sectors recognise and take behaviour change seriously, in other areas there is the challenge of answering questions about "what is behaviour". Recruiting people into behaviour change studies is challenging as people have different views about participating compared to medical/health studies. How behaviour change is explained to people is important, with examples like handwashing uptake during the pandemic, suggesting a need for standardised ways of explaining behaviour change to the public. |
| Academic 2 | Behavioural research is strong because of its multi-disciplinary nature.   That behavioural research has its strengths does not mean that all the questions are answered. Inherent complexity: Capturing outcomes from behavioural research can be difficult, and there is a need to protect the nuance in the research findings.  Limited effectiveness: Behavioural research has limitations and isn’t a magic bullet, it requires more honesty with funders about what behavioural research can achieve and emphasising the need to do good work well first. |  | Behavioural research terms, such as behavioural insights, are becoming more visible and being widely used. Recognition and appeal of behaviour change has broadened beyond health into sectors including AI, animal welfare, sustainability, safety, and security. | There is a challenge in using behavioural research case studies in teaching, as students struggle to understand. There is a need for behavioural researchers to accept that behavioural research is hard. |
| Government 1 | Behavioural science can often add value in a useful way during times of a tightening fiscal scenario.  There is a need for developing shared understanding of terminology and language that can be used interchangeably between departments. Having pre-agreed standardised science ready for emergencies is valuable but difficult to justify in advance.  Changing health behaviours is much more challenging than expected. | It is easy to get people interested initially in behavioural science, but trickier to engage them at a more serious level once the complexity of behaviour becomes apparent, and this complexity can reduce uptake of behavioural science. | When behavioural researchers are known to colleagues, they [colleagues] reach out. It is evident that colleagues are beginning to think in a more behaviourally informed way.  Behavioural science lacks presence in government, making it harder to attract good quality behavioural scientists, and making it more externally visible with accessible routes into the civil service would help. | Using the term behavioural science externally causes people to feel suspicious and as though they were going to be manipulated. When people have heard about behavioural insights, they usually talk about nudges, but there is a need to move beyond this limited understanding. |
| Government 2 | Behavioural research strengthens policy by keeping people more accountable and helping them focus policy problems better while considering unintended consequences.  Behavioural research has a strong track record of empirical evidence that can be applied in health contexts, such as smoking cessation. The evidence base for health and wellbeing applications is strong.  Existing evidence from behavioural research on choice architecture helped organisations articulate appropriate terminology, with the Competition and Markets Authority adopting this language to enable lawyers and economists to describe issues in their space. This addresses similar evidence usage problems across economics and social science disciplines. Some models exist to standardise the language used in evaluation for encouraging behaviours and determining which options are most effective. Behavioural research has established guidance and shared language.  Complexity creates challenges, as advice might not always be universally applicable. For instance, during COVID, Northern Ireland taking advice from wider Ireland led to difficulties. |  | Behavioural science as a profession has grown in the government sector in recent years, with more interest and recognition, contributing to both production and advocacy and use of behavioural science. There is a huge missed opportunity in business research, with significant value that social and behavioural research could add, though governments struggle with this integration.  Lack of knowledge and understanding that ontologies exist prevents people from learning from other evaluations. | Limited knowledge about what behavioural science is leads people to think it’s just about communication, resulting in it being underused in areas where it could make an impact. |
| Public&Third 1 | Making changes to barriers that are a priority for people is a positive shift, such as transitioning away from petitions to offering discounts on heat pumps. Using core behavioural models helps get to the bottom of challenges effectively. Now is considered the best time to highlight behavioural science’s value considering pressing issues like diet and climate change. Behavioural research helps communicate difficult evidence by setting out how data was collected and finding common priorities, even when results challenge stakeholders’ interests. Evidence-based approaches provide the best solutions that address problems, as demonstrated when a clinical advisory group was established to improve organ donation. It is important not to oversimplify scientific content while highlighting the value of behavioural science compared to other sciences.  Behavioural science buzzwords are used in health as it is fundamentally about behaviours. However, complicated operational definitions in mental health and suicide make it hard to agree on definitions and measurement, affecting how these issues are addressed. PR efforts should focus on how behavioural research works, why it works, examples, and clear terminology.  Organ donation research revealed complex readiness issues in Northern Ireland that differed from the rest of the UK when changing from opt-in to opt-out systems. Climate change presents the challenge of getting people to change behaviours, with participants noting message fatigue and the importance of broader context. What people say they will do is different from their actual behaviour, so it is important to separate the two. People lack knowledge of how to solve behavioural change issues.  Behavioural research tools like COM-B often diagnose problems but are not taken further to solve them. There are challenges changing interventions that aren't working, with examples like cardiovascular interventions being ineffective due to who they attract. There is a need to share insights about both successful and failed interventions, as learning from what didn’t work is equally valuable. Nudges are limited in effectiveness, and behavioural science can feel limited compared to traditional “big S” sciences.  Sometimes knowing less can be advantageous, as information without support for change can be counterproductive. Lack of understanding in behavioural change application can have counterproductive effects. During funding cuts, rushed behavioural research due to resource constraints can have detrimental effects. | There is enthusiasm for pilots, but rolling out more widely is challenging.  A new generation who had education in behavioural research are starting to have more growth in the field. | Most people are aware of what behaviours lead to a healthier life. Recognition of behavioural research is growing.   Low visibility of ongoing work leads to wasted time and difficulty knowing what others are working on or doing well. There is a need for PR to get people up to scratch on what behavioural research is and how it can be used. With many projects done on tight timelines, there is often no time to publish results to create visibility, and publishing more findings could create credibility for behavioural research. | Behavioural science can feel limited compared to the hard sciences (suggesting perceptions of it being less rigorous or legitimate).  Fatigue with climate change messages creates challenges for behavioural researchers in engaging audiences and highlights the importance of considering people’s broader context. There’s a lack of understanding of what behavioural research is and how to implement it properly in public health, and misconceptions about behavioural research can lead to people becoming hostile or feeling helpless when faced with these approaches. |
| Public&Third 2 | When people are shown clear examples of behavioural research applications, they can see the benefit. Evidence of cost-effectiveness when using behavioural science to inform interventions is particularly powerful for demonstrating value. The COVID-19 pandemic provided an opportunity to demonstrate the impact and relevance of behavioural insights. Organisations like the National Council for Voluntary Organisations (NCVO) can help with capacity building by demonstrating research value. A private company commissioned excellent behavioural research work, producing a thorough framework, and understood their organisation, the behavioural issue, and objectives, with clear methodology and tailored presentation. The Local Government Association (LGA) website provides examples of local authorities successfully using behavioural insights.   Emphasising the rigorous, science-based evidence base establishes credibility. Doing the research can help increase confidence in decision-making. There is a need to define social science (to help clarify the scientific foundations of behavioural research.)  There is a need for trans-disciplinary roles to bridge language gaps between disciplines and build on previous work. Collaboration is difficult when people don’t share common language or understand each other’s contexts. There is a need for clearer definition of terms  like social science. | It is recognised by the majority that there is a lot of interest and growing awareness of behavioural theories and methods. | There is growth in government adoption of behavioural research. For instance, Welsh Government has recently brought in behavioural scientists in the past 3 years. The majority have seen a lot of interest and growing awareness of behavioural theories and methods.  Only public health and environmental sciences are using behavioural research, so it needs to be more widely used. There is a need to raise awareness of good research in a competitive landscape where behavioural research competes against privately commissioned research. Building awareness of the importance of behavioural science and greater understanding of what it entails remains an ongoing challenge. | Scepticism about the role of social sciences is worse in organisations aligned to physical sciences. There are challenges around perceptions that behavioural change work is “social engineering,” requiring more narrative to decision-makers to debunk this misconception.  Lack of understanding of how behavioural science works creates funding challenges, as funders often ask for all activities to be mapped out before giving funding, but this may not be possible before the intervention is fully defined. |
| Public&Third 3 | There is a need for standardised guidelines, like NICE guidelines, to refer to in the absence of leadership. |  | There is a need to make behavioural research courses more accessible and visible. | Non-behavioural scientists often misconceive behavioural science as viewing ‘people as the problem that need fixing.’ This misconception is common even for the early stages of organisations that have embedded behavioural science.   In some organisations, bringing in behavioural research expertise is challenging as it goes ‘against the tide,’ with the extra barrier of needing to explain what behavioural science is, what it does, practitioners’ skillsets, and when they need to be involved. Some organisations don’t know where to place behavioural science within their organisational structure, suggesting more supportive leadership roles might help. |
| SMEs 1 | Behavioural science has provided some solid evidence for “what works,” such as in stopping smoking. Behavioural research brings the human elements into more tech-type roles.  However, people often want to modify evidence-based approaches despite engagement, requiring support for decision makers to trust that scientific evidence will deliver.  A key strength is the available and growing knowledge, but challenges in behavioural research are considered greater than the strengths. | People are naturally passionate about human behaviour, with curiosity driving this forward. Those with this mindset, passion and willingness to grow in their careers are drawn to the field. Selling to financial people and CEOs is particularly tricky. | There is a strong behavioural thread that runs through one company. There is growing interest from clients in applying behavioural science principles for competitive advantage. People from marketing show increasing awareness of behavioural science and its strategies as a growing approach. | There is a misconception that behavioural science still needs to be launched when it has already been established, and misunderstanding about how the behavioural component fits and how to use the research. Popularity creates challenges as people understand ‘nudges’ but not wider behavioural science, and everyone thinks they know human behaviour because they are human, leading to sweeping statements. There is a need for a new narrative as behavioural science has been viewed as ‘magic’ and ‘instantaneous’ rather than focused on sustained changes.  One challenge is that clients often have specific ideas without understanding the relevance of behavioural science, requiring shifting their thinking to align with behavioural approaches. Clients want hard data before implementation, but behavioural science is not purely numbers-based and requires a leap of faith. Even when people are engaged with evidence, they often want to modify approaches rather than follow them, delaying implementation. There is ongoing struggle with experimental processes and getting people to understand mathematical concepts like ‘significance’ rather than treating them as general assessments. |
| SMEs 2 | There are many good practitioners and experts who bring robustness to behavioural research. The health sector appreciate the evidence-based approach. While terminology has evolved, the scientific fundamentals remain the same.   In healthcare, the advantage of behavioural research is that practitioners understand research language. There is a lack of consistent ontology or naming convention for terminology, leading to difficulty in searching.   There are trust issues in the results when clients want quick wins for large-scale, complex problem.  Limited effectiveness: Behavioural research through communications cannot fix everything, requiring clarifying what can be addressed through communications and what other interventions are needed. | The client base is becoming much more interested in behavioural science with more education on behavioural research being seen. Some clients are happy to try behavioural research, as they are willing to do anything that will give them a commercial advantage. Adding a behavioural science lens to strengthen market research is found motivating and rewarding. | While previously there were relatively few people up to speed with behavioural science and expertise was lost when they moved on, this is happening less now as more people have experience and interest in behavioural science. Communications campaigns focused on positive social change are increasingly using behavioural research. | Despite plenty of skills and experience in the sector, how behavioural research is perceived and used can be challenging. Knowledge retention issues mean clients forget previous work, leading to duplication and being commissioned for the same work multiple times. Clients sometimes do not understand the research enough to realise it may not meet their expectations, often falling outside the remit of those dealing with results. Perception challenges create missed collaboration opportunities, such as government reluctance to work with companies perceived as “bad for you.” There is also tension in blending behavioural science with communications approaches that tend to be single-minded. |
| Cross-sector 1 | The value of behavioural research is shown in practical applications like understanding why product launches fail by examining colour and word meanings in specific locations. Its flexible methodological approach (quantitative, qualitative, focus groups, interviews) is valuable even when not strictly academic. It enables creative measurement of behaviour and deeper investigation of sensitive issues like negative driving behaviours that people would not admit in traditional surveys. Success examples include developing a one-stop shop, comprehensive sexual health website that increased STD test bookings and improving user experience beyond analytics. Behavioural science’s strength lies in understanding different needs and behaviours rather than assuming average users, taking context into account.  The methodology in behavioural research is shared and transferable across different disciplines, enabling wider application and collaboration.  More accurate and better definition of terminology would mitigate confusion and be more helpful. | People are interested and curious about behavioural science, and they like to learn about it and put it into practice. People are excited in the beginning but it can be hard to get them to actually use the methods and research. Using behavioural science and its benefits are not always understood, though some companies are happy to pursue this route. Interest may depend on company type, with pharmaceutical companies being more open due to their familiarity with experimentation and product development. Pure sales companies may be less interested due to their staff lacking research background. Some companies remain resistant to behavioural research. | Behavioural science and behavioural economics have expanded significantly over the past 10-15 years with more career opportunities and options available. Efforts to increase visibility include introducing behavioural research into classrooms to educate young children, which helps the field become better known and may influence future career choices.  Behavioural science is not always represented well at conferences. | There is confusion about what people mean by behavioural science, behavioural research and behaviour change, making these terms unclear. Some view design [jobs] as an art rather than a science and feel that behavioural research takes away their autonomy.  People do not know what they do not know about behavioural research when lacking that background, making behavioural science daunting and requiring demonstration of its value. Some companies remain resistant to behavioural research, and media misconceptions can lead to project shutdowns, as shown in a described case where parents’ concerns resulted in the removal of a website.  Other challenges include explaining research methods to others and clients having different ideas about how research should be conducted that researchers do not agree with. Co-design is not always popular with creative directors, though understanding behavioural research helps. |
| Cross-sector 2 | The evidence base of behavioural research makes it attractive because it can be quantified. There’s extensive experience and knowledge available, though it’s contextual, creating opportunities for collaboration.  COM-B has become widely embedded in market and commercial research, giving language and labels to practices that advertisers have long used, providing credibility and shared terminology. |  | Behavioural research has been used for a long time in agri-environmental sectors (though not always called BR) with good evidence around behavioural barriers and government recognition. This leads to employment of social researchers in DEFRA and other agencies, despite a long way to go. Behaviour change is used extensively in the NHS, and private industry is now embracing behavioural research following recognition that preventative care and digitalisation need prioritising, where behaviour change is key for pathway design and has been embraced quickly by private industry. COM-B is now everywhere beyond health and wellbeing, being used flexibly and effectively, especially in communications. | Those in the voting behaviour sphere don’t consider themselves part of behavioural research and view it as not credible or beneficial, describing it as “airy fairy” while preferring hard evidence of voting behaviour changes without applying theory. There are negative attitudes towards qualitative research when surrounded by quantitative researchers in both academia and industry.  In market research, clients’ lack of understanding of what behavioural research is makes them apprehensive and unwilling to engage with it, creating challenges and missed opportunities. |

## Appendix B2

**Table B2. Framework 2: Communication and translation**

| **Breakout room** | **Approaches and importance of research communication and translation** | **Challenges in communication, translation and implementation** |
| --- | --- | --- |
| Academic 1 | Strategies to popularise behaviour change include social media and advertising campaigns. Workshops to learn and promote different types of research are helpful for disseminating behavioural research findings. | Translating behavioural research faces challenges when environmental/contextual factors compete against the tools and techniques being provided, such as with alcohol consumption decisions. There are difficulties in reaching people who could benefit most from research and translating academic outputs into policy, which requires significant funding. |
| Academic 2 | To sell behavioural research, there is a need to increase social media use on the geopolitical stage and improve understanding of how to promote behavioural research. | Policymakers lack time to read behavioural research reports, creating challenges in how academics compress their work for practical application. The translational aspect is particularly difficult and requires capacity building, especially as theory is difficult to translate into practice and there's a need to build capacity in teaching translation skills. |
| Government 1 | None | None |
| Government 2 | None | There is a lack of ability to translate problems into behavioural research and social research, with economics dominating much of the field (the reverse of research translation). How to produce resources that meet policy colleagues’ specific needs is a problem. |
| Public&Third 1 | Effective communication approaches include using bite-sized case studies and webinars that are digestible for busy stakeholders, and sharing both strategic information and practical examples with evidence of why they work, with both the why and how being important. Key strategies involve finding common priorities when communicating results people do not want to hear, making behavioural research less academic and daunting while maintaining credibility, and using straightforward language without oversimplifying content. A comprehensive approach should explain how behavioural research works, why it works, and provide examples and terminology. Sharing insights through repositories in condensed ways allows people to access information easily while enabling deeper exploration, and academics should consider the needs of diverse audiences when leading knowledge-sharing opportunities.   Successful approaches involved establishing evidence-based advisory groups to develop solutions that directly address the core problem, such as in the case of organ donation, and focusing on addressing barriers that are actual priorities for people such as giving discounts on heat pumps. It is also important to communicate behavioural insights in a way that enables people unfamiliar with behaviour change to understand and take ownership of the solutions. It is important to highlight behavioural science’s value compared to other sciences.  Translating information and learning to wider scale sustained behavioural change is important. There would be benefits from making more practical information accessible. Demystifying behavioural change is important, and communication is important. | Communicating unwanted results is challenging, especially when ministers have vested interests in policy changes, though setting out evidence and finding common priorities can help. People struggle to translate complex academic information that is not always accessible. While academic findings are needed for credibility, making them more accessible and shorter would be helpful.   Translation challenges include getting people to change climate related-behaviours and the gap between prevention agendas and actual application, with translation into action often missing. There are also challenges where people stop at diagnosing problems using frameworks like COM-B without using them to actually solve problems, failing to take the application further. Despite extensive behaviour change research, implementation in practice is lacking, and while public health strives for this, there is poor understanding of how to implement it properly. It is challenging to feed into the fast-paced decision-making processes in policy and government.  Implementation challenges include the time required to examine readiness and implement changes, as seen with organ donation in Northern Ireland. There are resource constraints in creating sustained behavioral change and a challenge of scaling from pilots that generate enthusiasm to broader implementation across wider populations or settings. |
| Public&Third 2 | Case studies are effective when people are shown clear examples of how behavioural research has been used, allowing them to see the benefits. Using examples of research to illustrate behavioural science impact is effective for those less familiar with it, with case studies being particularly useful tools that bring research to life and demonstrate value. Effective case studies should provide relevant examples of behavioural research benefits to the people being served and clearly lay out how things were done.  A private company successfully produced an excellent behavioural response framework by thoroughly understanding the organisation and behavioural issue, outlining methodology in depth, and presenting it clearly and tailored to their needs. Evidence of cost-effectiveness in interventions is particularly powerful. There is a need for more translation from behavioural research to impact on policy. | Building awareness of the importance of behavioural science among participants remains challenging.   The slow pace of delivering behavioural change doesn’t meet expectations, creating translation challenges when working with postgraduate researchers. Challenges in conducting research without academic partners include whether in-house ethics processes are rigorous enough for academic spaces and the requirement for ethical review when disseminating findings through academic journals. |
| Public&Third 3 | An effective translation approach involves behavioural scientists speaking the language of users and addressing their specific problems to make the research relatable. Case studies, storytelling and narratives are needed to help people make sense of behavioural research. There is a recognised need to spend time communicating and translating behavioural research to practice. | None |
| SMEs 1 | Translation approaches include moving to more granular applications, such as helping charities be more impactful with email by tailoring messages beneficially rather than manipulatively. Storytelling works as a translation method, and tools like Google’s can help translate academic evidence into other sectors such as marketing.  There is a need to improve how evidence gets incorporated into policy making. | Misunderstanding about how to use behavioural research requires hand-holding to leverage the research effectively. Clients often have specific ideas without placing behavioural science at the forefront, needing help to shift their thinking. Clients want hard data before implementation, but behavioural science is not purely numbers-based. People often want to modify evidence-based approaches rather than follow them.  Implementation science is identified as a core problem. There is a conflicting challenge that while context matters and real-world experimentation is needed, testing is limited due to the many moving parts within systems. |
| SMEs 2 | Translation approaches include modifying behavioural research language for clients to ensure it is easy to understand, focusing on outcomes rather than process. Small bite-sized presentations that can be easily shared across organisations are helpful, and educating clients about the research process and what to expect is important. | Case studies may not always be representative of the broad range of sectors using behavioural research.  Regulatory issues sometimes prevent solutions from being put into practice. |
| Cross-sector 1 | Translation approaches include building on people’s willingness to learn and put behavioural research into practice incrementally, and encouraging the uptake of new methods that better inform practice. When explaining behavioural research to PPI groups, it is important to break it down into basic terms, speak clearly, create an open space without appearing as “the experts,” and involve people in analysis when appropriate. Since behavioural science can be daunting for those without that background, demonstrating its value is key, and defining terms more accurately would help reduce confusion. A successful approach involved developing a comprehensive one-stop website for young people on sexual health through a behavioural science project, with evidence showing increased STD test bookings as a result. Effective communication approaches include bringing behavioural research into classrooms to educate young children and improve understanding, using podcasts for storytelling with bite-sized chunks, and providing funding for evaluation frameworks for those who don't understand behavioural science. There is also potential for studying the best ways to communicate with the community itself. | Behavioural science can be daunting for those without that background, making it key to demonstrate its value. Explaining research methods to others is challenging. Additional challenges include getting clients to see the importance of taking certain approaches and difficulty speaking to audiences or replicating work with comparable audiences. Understanding why communication initiatives fail is also challenging, as seen with Global Association for Behavioural Science (GABS)’s mentoring programme that didn’t take off.  While good training courses are available, there are challenges in knowing how to apply learnings to specific areas.  Implementation problems include decision-making being driven by the loudest person rather than evidence, even in large companies with extensive research teams, sometimes making research a tick-box exercise. Media interference can derail projects creating anxiety about future projects. Political involvement rarely improves situations, with too many people finding problems for every solution. |
| Cross-sector 2 | Translation approaches include using LinkedIn and newsletters to share key research rather than academic papers, as these are more valuable and accessible for busy professionals. Cross-sector collaboration projects and making research more accessible through summaries rather than open access helps avoid academic bubbles, particularly for PhDs. Broader case studies or summarised evidence are more valuable than lengthy papers, and getting the language right when sharing research with non-academic colleagues helps build their capacity and capability. | Behavioural research frameworks like BCW or COM-B could be useful but need better translation and presentation for different areas to be credible and applicable to specific contexts. Academics, particularly in environmental behavioural research, get absorbed in their own work and expect people to use their models without making the research accessible. The challenge that people cannot sit down to read academic papers for hours makes broader case studies or summarised evidence more necessary. |

## Appendix B3

**Table B3. Framework 3: Collaboration, networking and engagement**

| **Breakout room** | **Cross-sector collaboration (need, value, and trends)** | **Experience sharing, networking and community building (trends, future, value, and challenges)** | **Stakeholder engagement (trends, need, and challenges)** |
| --- | --- | --- | --- |
| Academic 1 | Cross-sector collaboration exists through interdisciplinary teams developing interventions, such as working with students on a weight loss project that expanded into meat production interventions across different sectors. | Cross-sector networking includes engaging policy makers interested in behavioural science aspects. Wales has a community of practice for behavioural research that helps non-behavioural scientists get involved and use behavioural science.  Sharing experiences of difficulties in working with sectors is helpful.  There is a need to raise awareness in academia of collaborating on research funding proposals and to facilitate such opportunities. | There is a need for public involvement to include lived experiences meaningfully.  Industry partners have different expectations about research outcomes, as they often want science to confirm their preconceptions rather than accepting contrary findings. Policy makers seek simple solutions when behaviour change is more complex, wanting behavioural science to validate their assumptions without engaging with research findings. Companies sometimes perceive behavioural scientists as constantly seeking evidence.  Funders have expectations for large-scale outcomes, making them uninterested in small changes that academics identify as potentially having big impact but do not appear significant enough. There are conflicts of interest and ethical challenges when the built environment industry resist hearing that problems require behavioural interventions rather than technological or structural changes. |
| Academic 2 | There is a lack of collaboration, with researchers needing to talk and share more rather than working in isolation.   Multi-disciplinary discussion is essential for success in grants and projects.  Future collaboration can involve creating a collective of research councils with bigger budgets to champion multidisciplinary collaborations, encouraging early career researchers to work together, and using physical environment planning to create collaborative opportunities. Local authorities and government should be included as funding partners for national policy implementation, despite their budget constraints. | There is a need to create feedback mechanisms for industry practitioners to share experiences and include industry people in networks and forums.  Collaborative funding applications require having the right people involved and clear pathways to impact, supported by multidisciplinary discussion. However, time and resources for these conversations are limited, with teams often stretched by competing demands. There is a need for collective networking and community goals, recognising that collaboration strengthens the field despite existing jealousy over funding success. There is a need for early career researchers to work together, physical spaces and opportunities for collaboration, larger-scale forums including all career stages from PhD students to experienced researchers, and constant networking to nurture relationships. There is also a need to include local authorities in bids could strengthen applications and connect research to real policy implementation. | Access to PPI groups early in grants can aid success, which can be enabled with relationships to key organisations.   Funder expectations need managing through honest communication about potential failures alongside successes, and recognition that behavioural research is not cheap. As behavioural research has limitations and is not a magic bullet, there is a need to be more honest with funders about what it can achieve and to do good work well first. Success should be defined modestly rather than exaggerating outcomes, addressing the pressure to promise unrealistic results to secure funding. Increased education for funders and grant reviewers about realistic project possibilities is needed.  Engaging with stakeholders through co-design is difficult when limited by resources including time, research staff and funding. There are also difficulties where focus remains on research outputs rather than the main aim of informing practice. |
| Government 1 | There is a need for shared understanding of current research and better linkage between academia, third sector and government. Even within government, the evidence base could be more connected, with opportunities to tap into existing research rather than duplicating efforts.  Connections with academia is highly valued. | Existing networks include the emerging X-Gov BI Network cross-government community of practice, cross-government collaboration on behavioural science emergency response, and valuable informal networks for sharing knowledge and best practice. | There is a need for extensive stakeholder outreach to establish infrastructure and communications for encouraging uptake, involving multiple policy levers.   The multidisciplinary nature of policy making presents real challenges when trying to collaborate and engage with stakeholders across different disciplines. |
| Government 2 | There is a need for better communication channels between government and academia to discuss issues and evidence gaps regularly. While academic support is useful and secondments provide opportunities, there is a huge disconnect between sectors. Government departments publish research interests, but this is not sufficient for shaping access to academic research support, particularly where internal capacity is lacking. There is a need for government to raise awareness of projects and educate academia on bidding processes, with organisations like BR-UK consortium helping bridge this gap. Strengthening cross-sector government collaboration is important since much work is commissioned out, recognising government as one part of a bigger evidence picture. The Competition and Markets Authority adopted choice architecture language, enabling lawyers and economists to work together in describing issues. | We need to shape access to academics and research support for work where government lack in-house capacity. There is also a need for better networking mechanisms between government and academia around funding, including government raising awareness of available projects, organisations like BR-UK consortium facilitating connections, and educating researchers on bidding processes and resource requirements. | None |
| Public&Third 1 | Clinical advisory groups were established to improve organ donation by using evidence to provide solutions, demonstrating successful collaboration as a strength of behavioural research.  Local government offers collaborative advantages through quick audience access, small team efficiency, and rapid ethics processes. Different groups can be successful at different points of the behavioural research to impact journey. Planning behavioural research projects is challenging and requires early collaboration with multidisciplinary teams. | Real life stories of local successes and sharing why they worked is helpful. Low visibility of ongoing work wastes time and makes it difficult to know what others are doing well. There is a need for access to expertise when facing challenges. Networking challenges include conferences where people present duplicate projects with same findings, poorly structured sharing sessions that do not deliver, mismatched relevance due to participants being at different career stages. There are also difficulties in efficiently identifying whether people have done relevant work, and in accessing the right people and research. | Managing expectations involves communicating when interventions may be ineffective, such as cardiovascular interventions. Stakeholders often want short-term results rather than understanding that sustained change takes time; they need to understand that behavioural intervention outcomes can take a long time to have an impact, making expectation management with policy makers and decision makers particularly important.  There are challenges like communicating unwelcome results to ministers with vested interests, requiring careful evidence presentation and finding common priorities. Planning multidisciplinary projects requires buy-in from multiple stakeholders, which is complex. Pushback can indicate reaching the right audience, though targeting resistant groups generates more opposition. Stakeholders often delay decisions wanting more information, and it is challenging to feed into fast-paced government decision-making processes. Academic-led sharing opportunities tend to become geared toward academic needs rather than considering the requirements of other invited stakeholder groups, creating barriers to effective cross-sector engagement. |
| Public&Third 2 | There is a need for more trust and understanding when working across disciplines, requiring recognition of each other’s expertise. More opportunities are needed to bridge gaps between fields, particularly connecting researchers and NGOs.  Effective partnerships are beneficial but there is a need for more transdisciplinary roles to bridge gaps between fields and to ensure work is effectively built upon. These roles help translate across disciplines and improve collaboration. Cross-sector collaboration involved delivering behaviour change training that combined UCL course learning, council team input, and external expertise. A council has a research facilitator funded through NIHR who builds relationships with local universities and the council ethics team while managing research governance in-house. | Experience sharing systems: LGA (Local Government Association) provides examples of local authorities using behavioural insights.   Drawing from behavioural research across different sectors is helpful for adaptation. Health sector work provides inspiration, and cost-effectiveness evidence is powerful for informing interventions. Time and effort can be wasted starting from scratch rather than building on previous work.  There is a need for more cross-sector networks and opportunities to bridge gaps between fields, researchers, and NGOs. | Managing expectations involves addressing the slow pace of behavioural change delivery by postgraduate researchers that does not meet expectations. Strategic-level work requires significant thinking and time without immediate rewards, creating expectation challenges. Managing expectations requires leadership teams to understand how behavioural research works, including the time and resources required, and that it’s not a quick or guaranteed fix.  Funders often lack understanding of how behavioural science works, requiring detailed activity mapping before funding when interventions aren’t fully defined. Funding cycles may not allow enough time to demonstrate impact. There are difficulties with trust and understanding of each other’s work and roles, requiring recognition of expertise areas. Communication barriers arise when people do not speak each other’s language or understand contexts. These problems can occur even with partners within the same organisation. |
| Public&Third 3 | Behavioural data is kept separately rather than shared in the health sector, preventing aggregation of insights and learnings, and finding sharing is currently done informally. People work in silos with lack of integration across teams.  The increasing collaboration between academia and policy strengthens methodology and brings different disciplines together. | There is a need for mechanisms to share lessons learned, particularly through case studies. | None |
| SMEs 1 | There is a need to bridge the divide between academic/civic and commercial behavioural science sectors, as both can contribute positively. The UK has strong commercial, regulatory, and academic communities that need bringing together at leadership level to tackle complex issues. A new narrative is needed focusing on systematic, sustained behavioural change rather than anecdotal examples.  The existence of lots of collaborations and shared aims is recognised as a strength of behavioural research. | Behavioural science has a strong, tight-knit global community that shares best practices, which is beneficial especially for small teams. The UK is seen as the best place to practice behavioural science with strong infrastructure, described as a ‘melting pot of behavioural science,’ and benefits from recruiting diverse talent globally. London is unique (home to BIT) despite more practitioners in the USA - there is potential to learn from others, such as Manchester’s approach. | Managing expectations is important when fighting for behavioural research investment. Funders expect significant value for money, creating an immense challenge to show “big bang for your buck” with tight budgets. |
| SMEs 2 | Collaboration with other companies is valuable, exemplified by informal alliances with agencies in other countries. | Academia is siloed with colleagues unaware of each other’s work. Industry information is fragmented and difficult to find, with funding considerations creating barriers.  Networking challenges include expensive conferences requiring extended office absence, London-centric event locations creating access barriers. Shorter online events could improve accessibility. | Clients often expect quick results, but complex larger-scale behaviour problems can take longer time, so there is a matter of trustworthiness in the quick results. Clients sometimes lack sufficient understanding of research scope and timelines to realise projects may not address their specific expectations, particularly when research falls outside the remit of available funding. Communications cannot fix everything, requiring clear explanation of what can be addressed versus what needs other interventions. More client education about research processes and realistic expectations is important.  There are difficulties such as tension in blending behavioural science and single-minded communications approaches. Government reluctance to engage with companies perceived as “bad for you” leads to missed opportunities, despite successful examples like Scottish government collaborating with supermarket directors on food consumption data. Academic collaboration is valuable but challenging due to pace and budget constraints requiring longer-term commitments. |
| Cross-sector 1 | There is a need for more relationships to support nuances in behavioural research.   It is important to better understand people’s perspectives in behavioural research, particularly through co-design.  There's a need to create connection mechanisms like newsletters or yellow pages to link people with similar work. | Conferences help with connections and learning when support is not available. Proposed mechanisms include journals for failed studies and replications to avoid wasted resources, and funding top-up for improving and sharing research with the community. Canada’s approach to community events could serve as a template.  Available networks include BR-UK and CENTRE-UB which aim to fill gaps, and WhatsApp groups across disciplines that provide helpful advice. Twitter previously worked well but has declined, making WhatsApp the more active community platform. Informal mentorship cultivates a sense of community.  Despite having good screening processes, there is value in learning from others’ experiences. Professional membership bodies were expected to facilitate sharing and mentoring but this hasn’t materialised in practice. Mentorship and networks for advice are highly valuable, as training alone does  lead anywhere without ongoing support. Needed mechanisms include newsletters or yellow pages-style systems to connect with people doing similar work. There is a need to develop a sense of community across behavioural research. How networking is done matters - perceived hierarchy can discourage participation, requiring real-life rapport building beyond online interactions. | Access to public engagement involves using online approaches for greater reach, particularly for sensitive groups like domestic violence survivors where home-based participation is more appropriate. PPI groups are relied upon for advice and focus group insights, which is key to effective research. Engaging with PPI groups requires clear communication without appearing as “the experts” and involving people in analysis when appropriate.  Understanding user experience beyond analytics is valuable, as best practice based on “average users” is not realistic. There is a need to understand different needs and behaviours in context.  There are difficulties in stakeholder engagement when media attention leads to public concerns and project shutdowns, with ongoing worry about repetition. Political involvement rarely improves situations. Conflicting stakeholder views, such as young people supporting projects while parents oppose them, suggest a need for broader stakeholder engagement. |
| Cross-sector 2 | Cross-sector collaboration is valuable for PhD projects to avoid getting stuck in academic bubbles. Bringing problems to academics and researchers who provide thoughts on evidence and theories applied to experience is valued as a mutually beneficial collaboration.   Future collaboration can involve connecting behavioural research to personality profiling through platforms like LinkedIn polls, though this requires overcoming scepticism between different areas and willingness to join the dots. There is significant collective experience and knowledge available for collaboration, with organisations like Global Association for Behavioural Science (GABS) offering networking resources when personal connections aren’t available. | Peer groups are needed, similar to Market Research Society support networks. This would provide reactive access to sounding boards and confidential guidance for consolidating knowledge and confirming approaches, especially for isolated behavioural researchers.  There are many opportunities in behavioural research and policy and much community support in sustainability behavioural research.   Small-scale LinkedIn polls over two years, particularly on personality profiling, have shown potential for connecting behavioural research to this area. However, collaboration is challenging as some remain sceptical of either behavioural research or profiling. The key issue is joining up different fields and securing willingness across sectors to collaborate. | Decision tools for farmers failed because they weren’t co-designed with the farming community, demonstrating the need for proper stakeholder engagement.  There is a need to understand what people want from behavioural research, including entrepreneurial benefits for both leadership and consultants, and benefits for business clients.  Interdisciplinary research has a long way to go considering the difficulties in collaboration and engagement. |

## Appendix B4

**Table B4. Framework 4: Research practice and methodology**

| **Breakout room** | **Tailoring to contexts and systems thinking** | **Research best practice** |
| --- | --- | --- |
| Academic 1 | There are challenges in influencing decision-makers, and approaches often need to be tailored to specific contexts. There is a question of ‘can we change decision-makers’ views by adopting a systems-level approach?’ | A standardised “kite mark” to indicate what is scientifically backed, versus what is not, would help combat misinformation. Methodological and ethical standards vary across institutions, causing discrepancies. Researchers also face challenges when behavioural interventions clash with industry interests, such as resistance from the built environment sector to changing behaviours rather than technology. AI could handle routine data tasks, freeing researchers to focus on interpretation and the softer skills needed to apply behavioural science effectively. It is important to maintain openness and transparency in research and make open science more accessible. The growing use of behavioural research is driven by companies hiring analysts to leverage data for competitive advantage, especially in aggressive marketing environments. Participants stressed the importance of being open about expertise and focusing on quality over labels. Learning from what didn’t work is often seen as more valuable than successes. Recruitment for behaviour change studies is often harder than for medical research due to differing public perceptions. Contractual and administrative hurdles can delay or hinder the start of field trials. |
| Academic 2 | There is a need to identify which theories work for specific behaviours and which do not (a tailored approach). But there is also a need to find commonalities across behaviours and contexts to develop frameworks that address multiple behaviours, rather than one at a time. We need better evidence on sustaining behaviour change; and systems thinking may do this. Solving this could improve funding chances. | There is a need for access to large datasets, as relying on many small projects is less efficient. Using secondary data is seen as a more cost-effective approach. There is a responsibility to report failures and be honest with funders about what success realistically looks like. Behaviour change is often tailored to individuals, but identifying common patterns could help develop frameworks targeting multiple behaviours. Understanding how to sustain change remains limited; systems thinking may provide solutions and improve funding prospects. Capturing behavioural research outcomes is challenging because it is important to preserve the nuance involved. Data collection itself is a behaviour, and motivating people to participate requires targeted behavioural strategies. |
| Government 1 | Applying a behavioural lens in one sector, e.g., in government, does not always align with behavioural approaches in others and can sometimes backfire. Behavioural science often misses systems thinking, policy sequencing, and knock-on effects across sectors. Viewing one area behaviourally without considering others can backfire. A helicopter view is needed to spot cross-project impacts. | Academic literature can be hard to use when the methodology is not strong enough, whereas government applies a quality filter to ensure standards. There is a need for better coordination and shared access to existing research across academia, government, and the third sector. Expertise is not currently pooled or easily accessible. Participants suggested creating journals for failed studies and replications to avoid wasted effort. Access to academic journals is limited, making literature reviews difficult. |
| Government 2 | The timing for behavioural advice varies by context. During COVID, rapid input was crucial, but generally, the pace depends on the political cycle and the urgency of the issue—longstanding problems allow for a more relaxed timeline. The Department of Energy and Security offers valuable insights on green behaviours using systems thinking, but more work is needed. Existing models focus on individuals, yet organisations are complex groups of people, and organisational behavioural science remains underdeveloped. There is a recognised need for systems thinking in government to avoid rushing into risky interventions. | There is a need for clear, shared guidance and language, with tailored training for both beginners and those implementing it. Existing guidance is often too long - more accessible, user-centred resources is required. Long-term follow-up on interventions is often lacking, making it hard to assess lasting effects. AI could help monitor online content, but slow adoption is limiting its potential impact. Improving access to academic support and research could help organisations that lack in-house capacity. Many behavioural models focus on individuals, but organisations are more complex, comprising many individuals. Participants agreed that organisational behavioural science needs further development. In the UK, behavioural science in digital spaces is still emerging, with a growing but not yet mature academic community. Digital centres with experts are valuable. Market investigations often guide implementation, but rigorous testing is limited by reputational concerns, though new regulations may push firms to test remedies more. Data quality is limited due to infrequent and inconsistent collection. |
| Public & Third 1 | In Northern Ireland, the shift to opt-out organ donation was gradual, requiring careful assessment of readiness before changing legislation. Initial recommendations were not acted on immediately but were followed by monitoring until conditions were suitable for change. Participants noted fatigue with climate change messaging, emphasising the need to consider people’s broader context. Too much information without clear action can be demotivating and both the why and how need to be communicated, and that audiences progress at their own pace. Local authorities can engage audiences quickly, with streamlined processes for research and ethics. Pushback on interventions can be positive and it often indicates reaching disengaged or resistant groups, which may be the right audience to target. Positive change can happen when prioritising barriers that matter to people, e.g., moving from petitions to offering discounts on heat pumps. Practitioners need to adapt their work to anticipate what is next in the behavioural change process. Being prepared for the next step is key. | Sharing what has not worked is just as valuable as sharing successes—both are essential for learning. There is strong enthusiasm for pilot projects, but scaling them up for wider implementation remains difficult. Changing climate behaviours remains challenging, especially with the gap between what people say and what they actually do. In areas like mental health and suicide, agreeing on definitions and measures is difficult, affecting how work is carried out. Evaluating which intervention is most effective is also challenging due to varied evaluation methods. Smaller sample sizes make robust evaluation more challenging. Participants value having accessible resources and expertise to consult when facing challenges in behavioural research. |
| Public & Third 2 | Using relevant examples that clearly show the benefits of behavioural research for the target audience makes case studies very helpful, especially when highlighting how it differs from business-as-usual approaches. One organisation adapted existing resources to develop bespoke internal training. A positive example of commissioned work came from a private company that created a thorough, well-presented behavioural framework, tailored specifically to the organisation’s needs. Participants also highlighted the value of drawing on behavioural research from other sectors, particularly noting inspiration from successful work in the health sector. Participants described difficulties finding longitudinal studies relevant to their work but recently came across one that directly supported the development of their own programmes. Others also noted challenges in finding research examples specific to their areas of interest. | Participants highlighted challenges competing with privately commissioned research and raising awareness of quality work. Conducting research without academic partners raises questions about the rigor of in-house ethics, which is needed for academic publication. Many are early in developing research governance and feel uncertain about it. Emphasising a rigorous, science-based evidence base distinguishes research from usual approaches. Most feel they are just beginning their journey with research governance and experience frustration and uncertainty. Conducting research without academic partners raises concerns about the rigor of in-house ethics, which is needed for publishing in academic journals. Some have heard of hiring external ethics panels but view this sceptically. A few councils have NIH-funded research facilitators who manage ethics and help build links with universities, improving governance. Honesty about challenges, not just benefits and clear explanations of methods used is valued. There is often insufficient time to fully demonstrate the impact of behavioural research. |
| Public&Third 3 | Behavioural research is often viewed as focusing on individual behaviour change rather than addressing wider systems. | In the absence of clear leadership, there is a need for trusted guidelines, like those from NICE, to provide direction. Need to understand how AI could support BR. In the health sector, behavioural data is often collected but kept separate, preventing aggregation of insights. Sharing tends to be informal rather than systematic. Behavioural researchers in government often apply existing theories rather than developing new ones. |
| SMEs 1 | There is a tension between the need to test in real-world settings, where context matters, and the practical limitations of doing so because of system complexity. Participants expressed a desire to balance this by doing more upstream, controlled work first, e.g, lab-based research, before tailoring it to real-world contexts. Context matters, so real-world testing is essential, but complexity limits how much can be tested in practice. | The behavioural science community is seen as globally strong and collaborative, particularly helpful for smaller teams. The UK’s strong regulatory environment is viewed as a major asset, providing protection and credibility. There’s a call for leadership to unite sectors (academic, civic, commercial) to tackle complex issues like sustainability, health, and finance. Participants want to shift the narrative from entertaining anecdotes to systematic, data-driven, sustained behaviour change. BR-UK could lead on ethical, thoughtful use of AI in behavioural science, with clear guidance on when and how it should be applied. There is a perceived divide between academia/civic sectors and commercial behavioural science, where the former is seen as ‘doing good’ and the latter as profit-driven. There are strong, ethical practices in the commercial sector, supported by robust regulation (e.g., FCA, Competition and Markets Authority) that protect everyone and promote good outcomes. The UK benefits from a healthy mix of commercial, regulatory, academic, and civic behavioural science communities. Recommendations can be made using existing public data, like from charities, but limited data access in new sectors makes research challenging, especially when organisations are reluctant to share or publish information. There can be tension between wanting real-world, context-dependent testing and the desire to conduct upstream, controlled work first. |
| SMEs 2 | None | There is a significant lack of published case studies across industries because clients often withhold results or collaboration details, making it hard to share insights without breaching confidentiality. Working with SMEs involves researching harder-to-reach communities with larger sample sizes and often fewer bureaucratic barriers like ethics delays. Collaboration with academics is valuable but requires longer timelines, as pace and budget constraints can be limiting. |
| Cross-sector 1 | Often commissioned to tackle very specific behavioural issues relevant only to niche groups. ‘Average users’ don’t really exist—best practice needs to reflect diverse needs and behaviours and needs to consider context. Communications teams often take a flexible approach to behavioural science, using a mix of methods like quantitative research, focus groups, and interviews. While not always the most academic, this flexibility is seen as a strength. Moving more work online has also helped reach wider audiences, including those affected by domestic violence, where remote participation was safer and more appropriate than in-person sessions. Courses on behavioural science are good, but there’s a gap in understanding how to apply the learning to a specific area of work. Systems mapping and real-world research is a first step before engaging with people. | There is a reliance on PPI groups to guide research and improve focus group relevance. They advocate shifting from self-reporting to better measures of change. Openness about expertise is important, with emphasis on protecting quality over labels. A journal for failed studies and replications was suggested to reduce wasted effort. Early-career clinicians are encouraged to adopt new methods to improve practice. In industry, behavioural science drives creative measurement and experimentation beyond self-reporting. For example, studies on negative driving behaviours use behavioural approaches to uncover issues people wouldn’t admit in traditional surveys. Challenges like "imposter participants", those misrepresenting themselves for incentives also highlight the need for better participant verification. |
| Cross-sector 2 | Initial impressions were that Behaviour Change Wheel/COM-B could be useful, but it needs to be better presented and tailored to different contexts to gain credibility. Seen as having potential, but not yet proven in this area. | Capability in behavioural research is low and a low priority, leaving many unsure where to turn for guidance. There is an interest in using generative AI to consolidate existing data and apply insights across different fields. There is potential for a search engine or AI tool (by keyword) to quickly find tailored behavioural science examples across sectors. Confidentiality and preserving context are challenges. Much data remains unanalysed, so consolidating evidence is crucial for wider use. Video data holds untapped potential, such as analysing body language and emotional intelligence in virtual meetings. Qualitative research is sometimes undervalued in environments dominated by quantitative methods. There is also a lack of capability and metrics for effectively measuring the economic and environmental impacts of behaviour change. There is a growing push for cross-sector collaboration, especially in PhD projects, to avoid academic isolation and make research more accessible through concise summaries rather than just open access. |

## Appendix B5

**Table B5. Framework 5: Behavioural research impact**

| **Breakout room** | **BR and Policy** | **Considering impact in BR** |
| --- | --- | --- |
| Academic 1 | Influencing decision makers is challenging because interventions often need to be tailored to specific contexts. | Efforts to provide tools for healthier alcohol decisions face challenges because environmental influences often counteract them. The key issue is translating research into practical approaches that reach and benefit those most in need. Behavioural research can be experimentally tested to demonstrate impact. |
| Academic 2 | Local authorities and government need to be involved, as they play a crucial role in shaping national policy. | There is a need to understand how to design interventions that sustain behaviour change over the long term. Success should be defined realistically, with honesty about potential outcomes when engaging funders. There is also a need for greater emphasis on collective impact rather than solely individual achievements, particularly in relation to frameworks like the REF. |
| Government 1 | Behavioural research can be applied to narrow projects. One example involved engaging directly with people in arrears on rates bills, identifying barriers, and co-designing practical solutions, which led to reduced arrears and court costs despite the project’s small scale. Even with strong behavioural evidence and clear policy recommendations, outcomes are not always politically acceptable. | There needs to be more scepticism about the attitude-behaviour gap to encourages more theory-driven analysis of intervention impacts. However, behavioural science often overlooks knock-on effects across sectors, and applying it in one area without considering others can backfire. A coordinated overview is needed to identify cross-project consequences. The impact of behavioural science is difficult to define and measure in a clear, straightforward manner. |
| Government 2 | Behavioural science in government should focus both on producing high-quality work and advocating for its use. Embedding it more systematically, such as including a dedicated behavioural science check in policy development processes, would help ensure it is routinely considered. There is a challenge around having enough resource to produce what policy colleagues actually need from behavioural research. Behavioural research strengthens policy by improving accountability, helping to clarify the problem, and encouraging consideration of unintended consequences. Behavioural insights were applied effectively during COVID-19, raising the question of how to replicate this success in future crises. | Behavioural research is often cut early, so securing external funding and publishing successful outcomes can help sustain support. It is difficult to expand behavioural science use because people want to see clear impact in their own area first. Resources to support impact measurement would be helpful. Measuring the direct impact of behavioural research in government is difficult due to the complexity of issues and focus on problem definition. |
| Public&Third 1 | Work Northern Ireland revealed a lack of readiness for shifting from opt-in to opt-out for organ donation, with complexities distinct from the rest of the UK. For some behaviours, like smoking, policy change took a long time to translate into meaningful outcomes. Participants also suggested a ‘what works’ centre to give quick access to relevant behavioural insights, with the option to explore further for policy development. | Smaller sample sizes make evaluation more difficult. Differing methods complicate comparing intervention success. It is important to recognize that intervention outcomes often take time to materialize. In mental health and suicide research, complex definitions make agreement and measurement difficult, affecting how interventions are designed. Additionally, there is pressure to show short-term results, even when sustained change is the ultimate goal. Sustaining behavioural change is very difficult to achieve and translating learnings to wider, long-term behaviour change remains a challenge. Preparing for what comes next in the behaviour change process would be helpful. Funding for longer interventions beyond pilot stages is needed. However, comparing the success of different pilots is challenging because they are often evaluated using inconsistent methods. |
| Public&Third 2 | There is a need for more research funding specifically aimed at local authorities or partnerships with them to address health behaviours they are responsible for. More work is needed to translate behavioural research into tangible policy impact. | Small sample sizes make evaluation harder, and varied measurement approaches complicate comparing pilot success. It is also important to acknowledge that intervention impacts often take considerable time to become evident. Short funding cycles often do not allow enough time to fully demonstrate the impact of behavioural research. Work done at the strategic level demands significant time and effort and can be less immediately rewarding. |
| Public&Third 3 | Charismatic leadership is viewed as essential for gaining funding and influence in policy. There is scope to better understand and influence the behaviours of policymakers and professionals. | A clear, visual tool illustrating the impact of embedding behavioural science would be valuable. Funding timelines are often too short to fully demonstrate the impact of behavioural research. |
| SMEs 1 | None | Behavioural science has already been established; now the focus is on detailed application, such as helping charities tailor emails effectively to genuinely benefit the public rather than just serving company interests. There is a need to shift the narrative around behavioural science from being seen as a quick fix to focusing on sustained, long-term change. A key challenge with limited funding is demonstrating significant impact or value for money. |
| SMEs 2 | None | Return on investment is crucial; behavioural research should deliver actionable insights that benefit all stakeholders and can be practically applied within organisations. |
| Cross-sector 1 | None | Measuring impact in policy research is challenging due to uncertainty about which variables best capture meaningful change. There is hope that increased learning will enable more sustainable, long-term behavioural change. |
| Cross-sector 2 | None | There is a lack of capability and clear metrics for monitoring and evaluating economic and environmental impacts of behaviour change. |

## Appendix B6

**Table B6. Framework 6: Funding, resources, capacity and accessibility**

| **Breakout room** | **Accessibility of BR and related resources** | **Central BR resource bank strategies** | **Issue of funding and related challenges** | **Limited capacity and resources** |
| --- | --- | --- | --- | --- |
| Academic 1 | None | None | Translating academic outputs into policy takes a lot of funding. There is a lack of awareness across academia of how researchers can collaborate more on research funding proposals. Example of applying to get funding but turned down because it was being tailored to individual settings but the funders wanted one intervention that could be used in all schools. | None |
| Academic 2 | None | None | Academics need recognition from research councils that behavioural research is not cheap and cannot be funded cheaply – they need more resources. More money to social sciences is required and less time spent applying for resource grants. People are our resources and they are expensive – particularly when we need to do something quickly. Behavioural researchers are not bold enough in asking for funding compared to other scientific disciplines. Funding should be considered on an ongoing manner rather than just short-term funding –time is wasted on applying for funding during latter stages of projects in order to move onto next thing. Funding requirements are also inconsistent. Teams often have to strip back proposed projects to apply for grants that would not be able to fund bigger projects - we need a collective of numerous research councils and funding to create bigger budgets. It may also be helpful to provide some behavioural research education targeted at funders and grant reviewers as they should be aware of issues that might arise on funded projects and they need to know what is really possible. | There is no/limited capacity in universities. There is no time/resource to discuss collectively with colleagues about findings – teams are constantly downsizing workload to make room for new work. Co-production may fund applications but timelines of project do not allow for the proposed project, particularly when using co-production methods – the ‘spirit’ of projects may fail due to time constraints. It is difficult to do co-design and engage with relevant stakeholders appropriately when you are limited in resources (time/research staff/funding) and then when the focus is on research outputs (in most cases), when most researchers want to inform practice. |
| Government 1 | People working in government often do not have access to academic journals, so it can be difficult to do a literature review. Expertise is not pooled and available to access though. Government evidence does not even come up in a search. | A central repository for government records would be helpful. Repository of case studies of previous behavioural science programmes as a central resource would help to improve understanding. | Budget restrictions mean that behavioural research is often the first thing that goes. There is a desire for a pathway for training and development for behavioural scientists internally but there are budget and time restrictions on this. Behavioural research is being sidelined, and aiming for full cost recovery is a difficult model to sustain. There are cases where behavioural research could have a big impact but being consultancy-based cost is very often a barrier. Teams want to do appropriate research for each area but need to spend a lot more money to do it properly, and so they provide advice and consultancy. | Teams spend fair amount of time trying to raise capability, and as people move the teams lose expertise and learnings. At the moment teams rely on outsourcing a lot of behavioural science work. |
| Government 2 | The civil service does not have access to academic journals. There are also issues with the guidance that exists, sometimes it is very lengthy. Guidance needs to be good, user centred, accessible. | Behavioural research guidance needs to be good, user centred, accessible. | Lack of funding is a challenge and would be welcome as it could be used to hire someone to do the research. Giving certain teams/departments funding to utilise behavioural science can also be useful. Behavioural research is often cut first, if there is a potential to have a funding opportunity outside of the host organisation that can be helpful, as well as getting teams to publish successful results. | Commissioning externally happens due to lack of resources or capability internally. |
| Public&Third 1 | Academics often own opportunities for sharing insights and it can become geared towards these groups. If academics lead it, it should consider needs of other groups invited. Seeing bite sized case studies and webinars makes information accessible and relatable. Having resources to refer to is helpful. Examples of how behavioural research can deliver are needed. People struggle to translate academic information, which is more complex and not always accessible. There is a need to make behavioural research less academic, daunting and more accessible. People otherwise can get hostile or feel helpless. Participant from public health agency agrees demystifying behavioural change important. Academic findings are needed to take things seriously, but making the findings more accessible/shorter can be helpful. Sharing assets on strategic information but also more practical examples helps and having the evidence of why it works is helpful. It is tricky to know what other individuals are working on/ what are they doing well. Behavioural research needs a PR push - how it works, why it works, examples, terminology. People need access to expertise when you have challenges, how can this work? | One participant discussed a repository for behavioural interventions and what has worked could be helpful. A what works centre would be helpful in a way that can allow people to access insights quickly on what is relevant to them with the option to learn more would be useful for policy. What works Ireland evidence hub is a useful example. | Local government funding is low and behavioural research can be more prominent during funding cuts to help make decisions. There is a need for funding longer interventions beyond pilots. There is an overall question of who pays. | None |
| Public&Third 2 | Reports of struggling to find longitudinal studies in area of interest, but recently found studies that helped directly with developing their own programmes. There can be a lot of wasted time and effort starting again from scratch, rather than building on previous work, and there can be difficulties finding examples of research in specific areas. Most have found it helpful to look at examples of behavioural research from other sectors and adapt it to their own area. LGA website has examples of local authorities using behavioural insights. | None | We need more research calls targeted at local authorities, or partnerships including local authorities, that aim to investigate health behaviours that they are responsible for. There are limited examples of external training, due to lack of funds available. The funding cycle may not allow for enough time to show impact. There is a lack of understanding of how behavioural science works, and teams are often asked for all activities to be mapped out before funding will be given but they may not be able to do that before the intervention is fully defined. It is harder to use public funding for behavioural research, and research in general, as organisations need to focus on what they are contractually required to do. There is a lack of funding available for behavioural research, particularly in conservation work. | Capacity is an issue for many in behavioural research. Having someone in-post focused on embedding behavioural research would help (a “research facilitator”). Examples of using postgrads, but the slow pace of delivering behavioural change does not meet their expectations. Teams have worked with some universities but can be tricky for academics in terms of when the work has to be done and their capacity - there is a need for more trans-disciplinary roles so people can speak both languages and ensure previous work is built on. Teams may also have lots of data, but having the time to evaluate it and develop an intervention is a challenge. There was general agreement that partnerships can be beneficial but that it takes time to develop an effective partnership. Often the work teams are asked to do is at a strategic level, which requires a lot of thinking and time, and is not as immediately rewarding. Finally, there is often not enough time to demonstrate the impact of behavioural research. There is a lack of continuity (due to staff leaving) which can cause gaps in knowledge and result in work being done that is similar to what has been done before. |
| Public&Third 3 | Question of how courses can be more accessible and more visible. Teams try to be champions of behavioural research, but there are barriers for colleagues in attending internal training events or webinars. e.g. lack of time, other competing priorities, other processes, procedures and standards that people are required to do in their work. | Teams unsure how to commission behavioural research projects, there could be more guidance on developing appropriate specs. | Procurement is often slow, unless in emergency situations. | None |
| SMEs 1 | None | Funding to create an active repository of knowledge hub with AI appropriate prompts/ hierarchy could make things easy to find. | None | None |
| SMEs 2 | People usually rely on own networks or searching on LinkedIn. ESOMAR (European Society for Opinion and Marketing Research) have a search that can be used internationally to find an expert. Likely that AI could be used for this in the near future. Events are organised by lots of different bodies - centralising things would be helpful. Discussion and networking at conferences is great but getting to large events is often a challenge as they are often very expensive and require a longer stretch out of the office. If there were some shorter, online events might make it easier to attend and access. There is a lack of published case studies across industry breadth often due to client confidentiality concerns about results and their use of behavioural research consultancy. | Small bite sized presentations which can be shared easily with the rest of the organisation are helpful. A database that records what areas people work in that you could search for collaborators would be helpful. A central bank of training resources would be helpful. | Teams would like to see more funding available for collaboration between academia and industry. Funders can sometimes question the costs associated with working with external parties. Collaboration with academics is valuable but needs to be over a longer period - Pace and budget are sometimes prohibitive. | In many cases clients are not able to fully take advantage of everything that the research gives them.  For example, just having to focus on one particular strand, e.g., a comms strategy but the research has actually given them much than this - this is sometimes down to time, resources or funding. Sometimes clients haven’t really understood the research enough to realise that it does not always meet their expectations, as this often falls outside their remit of people dealing with the results. However, in the past there were relatively few clients who were up to speed with behavioural science and then when they move on that expertise gets lost, but this is happening less now that more people have experience and interest in behavioural science. |
| Cross-sector 1 | Sometimes support is not available but making connections and learning more through conferences, for example, will help. An example of a behavioural science project resulting in the development of a website – a one stop shop for young people on sexual health, comprehensive space. Evidence that bookings for STD tests increased as a result. Having something like BR-UK as a source of information is helpful. | It would be great to a have a journal for failed studies and journal where you can publish replications. Waste of resources goes into repeating things which others have done and have not worked. Having some sort of newsletter or way to connect to people who have done something similar to you, like a yellow pages to help find someone to connect to. | Cost can sometimes be an issue, particularly if stakeholders are not fully invested in the results. It would be useful to have a fund to apply to top up funding to improve research which can then be shared with the community. Teams would like more applied funding rather than theoretical developments. | None |
| Cross-sector 2 | Reports of not reading an academic paper in years, but instead seeing key research via LinkedIn and newsletters which is much more valuable than say Google scholar. We need behavioural research to be more accessible - summarised rather than open access. Most cannot just sit down for a couple of hours and read a paper, but broader case study or summarised evidence more valuable. There is lots to be said for academics making their research accessible as well, particularly in environmental side of BR, can get absorbed in own little world. There is an absence of tools and methods and capacity or ability to convince seniors in relation to the benefits of BR, including cost. Participants were interested in accumulation of information- generative AI to consolidate what is already know based on data we already have to apply to other areas. One participant has access to a resource that can store papers as a repository and uses AI to ask questions of it to save time. | Imagine a search engine or bot where could type e.g. ‘COM-B, leadership, automotive industry’ it came up with an example of where this was used. It is useful to be able to bring a problem to academics and researchers giving thoughts on evidence and theories applied to their experience. | Budgets are getting smaller and teams need to do more for less, leading to methods being decided by the budget rather than the need. Behavioural research has a premium and teams do not want to pay, or see it as an add on. When it is a small group with limited budget, they need to make the case for behavioural research and have to make a cost argument, but it can be hard to convince people at senior level that it is a cost saving exercise. Teams do not have the tools, methods, capacity or ability to convince seniors in relation to the benefits of behavioural research, including cost. An example of working with Defra and Natural England – they are happy to do behavioural research but limit the scope and scale, e.g. will do surveys but not the in-depth qualitative work that is required, due to the increased cost attached. | There is no time to just sit down and read a paper, so broader case study or summarised evidence is more valuable. Capability is quite low and low priority so teams would not know who to go to for behavioural research guidance. |

## Appendix B7

**Table B7. Framework 7: Training and career development**

| **Breakout room** | **Importance of, need for and barriers to training** | **Examples of and potential approaches to research training** | **BR career routes and researcher confidence** |
| --- | --- | --- | --- |
| Academic 1 | It is important to create a critical mass of early career researchers, PhD student and behavioural researchers who can integrate into other areas of research. | Behaviour change wheel and COM-B model to teach speech and language students is used and also used to develop training for individual settings. Using workshops to learn and promote different types of research are helpful. | None |
| Academic 2 | Theory is difficult to translate into practice. We need to build capacity in teaching. Behavioural research is also usually a ‘sexy add on’ which is tokenistic, and so often those using behavioural research do not have training. Researchers need to accept that behavioural research is hard and students can struggle to understand. | Training in influencing policy (including how to operate with policy makers and how to be in the environment alongside policy makers) and how to have conversations /create networks are needed. Agreement that training and guidance is needed both for people who can action it and also at an introductory level - a need for appropriate level of guidance across the organisation. Example given of using behavioural research case studies to improve understanding but students still struggle to understand. | There is a question around how we break into industry. Students ignore industry and focus on policy. How do we create opportunities for students to explore this area? Potential solution: alumni networks could be useful, as well as including industry in academic networks and forums. Money is the elephant in the room – behavioural scientists aren’t bold enough to ask for funding (in comparison with other sciences – e.g. physics). |
| Government 1 | Upskilling policy makers in government to embed behavioural research would be very beneficial, but challenging - capability or training programme to build on people’s interest and embed those in sector teams could help improve this. We want to be in a position to do good behavioural research when its required – e.g. in emergencies – what is needed is evidence but we are often not in a place to do the research. Helpful to scope pathways for training and developing behavioural scientists internally – but there are budget and time restrictions on this. For building broader capability in the system, it is a slow creep to building understanding. | None | It would be helpful to make behavioural research more externally visible and make routes into the civil service more accessible. Behavioural science does not have much of a presence in government, makes it harder to attract good quality behavioural scientists to government. It would be helpful to have scope for pathway for training and development for behavioural scientists internally – but there are budget and time restrictions on this. There is a perception that there is no progression for behavioural science within government and people leave because there is no scope for promotion. Many people who move into behavioural research are marketers and social researchers. |
| Government 2 | Resources for upskilling can be helpful where behavioural science is not embedded and to get organisations to think behaviourally, which is a big challenge. Both training and guidance are needed for people who use behavioural research and those at a more introductory level as there is such a hesitancy to use behavioural research and be evidence based. An appropriate level of guidance is needed across organisations. Training courses need to be more accessible, L&D can be quite expensive. The challenge with a more dispersed approach is lack of quality control and consistency sometimes, potentially something which having a more formal profession would solve (not that this does not bring its own challenges). | Upskilling in government in bite sized opportunities could be useful. Training could target specific professions and departments for whom behavioural research is most relevant and where upskilling makes sense | Secondments by behavioural researchers create opportunity and can involve people coming in to spend a year on a particular research question. There are more secondments going on generally and these are not just a one-way benefit. They also allow people to get experience on how organisations function. In an example from Wales, they created a mini SPI-B for cross department work, which also benefitted participants as they learnt how government works. |
| Public&Third 1 | There are two key elements: 1, getting people up to scratch so people know what behavioural research is, but 2, how it can actually be used. Training can feel disjointed and people will then not implement learnings from it. | Training needs to think about enablement. Example from a council that training may not work if not done well - the council changed their training to make behavioural change into a game. They had done behavioural analysis on why a training course alone did not work, then focused on enablement, which required showing a mechanism and thinking about how it works, then changing it. | We have a new generation now who had education in behavioural research and these people are starting to have more career growth. |
| Public&Third 2 | People try to use behavioural research (e.g. COM-B) in their work without having had training. There is a need for more people to be trained and need to recruit people who can upskill others in behavioural science. Some examples of in-house training exist but is overall limited due to capacity. There are also limited examples of external training, due to lack of funds available. | Examples of attending behavioural science training at a university, getting funding to develop research skills training for a council incorporating behavioural science, developing in house training on social sciences and behavioural science frameworks (although limited due to capacity), running behaviour change training for entire public health teams based on learnings from university courses and external teams/experts, and adapting resources to create bespoke internal training. | None |
| Public&Third 3 | We need training for leaders - how to champion behavioural research, how to build behavioural research into strategic planning. The group try to be champions of behavioural research, but there are barriers for colleagues in attending internal training events or webinars e.g. lack of time, other competing priorities, other processes, procedures and standards that people are required to do in their work. | Courses need to be more accessible and visible. Training and guidance also should be tailored to audiences. | Welsh Govt have recently brought in behavioural scientists, particularly over the past 3 years. There is variability across departments and organisations - some have had little growth and establishment, depending on the sector or organisation. There are not as many job titles with ‘Behaviour’ as expected. Some feel like a fraud in calling oneself a behavioural scientist if not formally had training in it. |
| SMEs 1 | None | There is a potential missed opportunity in government. We need to embed behavioural science in general skills and understanding before people become the decision makers e.g. biases, tricks, critical thinking; move upstream and teach children and young people. We should step away from sector/ discipline based learning. Training within agencies is good and adopted well. Helpful to provide continual nudging rather than assuming knowledge. Training within agencies is good and adopted well, but when working with big organisations there is often a groundswell of younger staff with BSc/Masters/in house training, but also a group of people who do not have this at a slightly older/senior level who want to progress. | People are naturally passionate about human behaviour, curiosity drives this forward. People with the same mindset, passion and willingness to grow in their careers. In big organisations it appears there is often a groundswell of younger staff with degrees or inhouse training, but a senior group who are going to be leaders who do not have behavioural research training - should behavioural science be incorporated into MBA programmes to ensure we have more behaviourally informed executives. Behavioural science appears to be an outlet for those with a background in psychology or behavioural science who perhaps did not find themselves working directly in that field. It is an opportunity to meaningfully apply the knowledge to a wider variety of industries. Lots of people from psychology backgrounds underestimate the skills they bring to a range of roles. Doing an MBA does not set you apart from other MBA people, but if you understand behavioural research it sets you apart – it is like you “bring a gun to a sword fight”. We need to help people to make the link between psychology and e.g. marketing, advertising. |
| SMEs 2 | None | Training mostly focuses on entry level/mid-level and there are gaps for senior career stages (where there is sometimes reluctance to go on training as maybe suggests a lack of knowledge which they perhaps would be expected to have). We need more forums rather than a taught course. Just knowing about courses, finding out about them in the first place, is challenging - things are quite fragmented. Sometimes there are funding considerations for attending. CPD is not necessarily that important, except if it is useful and relevant then that is more important. Certificates can be motivational for people in encouraging them to attend. Example of delivering internal training on behavioural science over five weeks based on their own knowledge/ expertise - it would have been helpful if there was a central bank of training resources. Second example of one person attempting to teach people/upskill people within the organisation, but this was too big of a hurdle so they now focus expertise within a smaller team. | It would be useful to have a centralised job board for behavioural science jobs. There are bodies trying to do that but would be helpful to graduates. There are a lot of courses available now, giving a big supply of graduates but there may not necessarily the jobs available to them. There are some people taking this into their own hands – including WhatsApp group that is for job adverts with 3000+ people. When hiring – is language a factor in finding a skills match? Describing the skill set rather than titles is a good way of finding the right people. Graduates with behavioural science background have strong background and good grasp on behavioural science and tend to be very high quality. |
| Cross-sector 1 | None | There are potential benefits to bringing behavioural research/science into the classroom to educate young children, this gives time to learn terminology and might improve understanding and inform career choices.  CPD is not necessarily that important, except if it’s useful and relevant then that is more important. Certificates can be motivational for people in encouraging them to attend. Within the teams, time is spent on what did not work, which often teaches more than the examples that did work. | What makes a behavioural researcher? When do you call yourself that? Does it maybe depend on how much you sell yourself. We need to make room for networking at events, to build rapport in real life situations – not all online. Sometimes perceived hierarchy might put people off asking questions, for example. Behavioural research and behavioural economics are fields that are expanding - 10-15 years ago we could not do it the way we can now. There are more options for people coming into the field. Bringing behavioural research into schools might also improve understanding and inform career choices. |
| Cross-sector 2 | None | Training to support someone to be a champion for behavioural research in your organisation would be helpful. | None |

## Appendix B8

**Table B8. Framework 8: Leadership, embedding and prioritisation**

| **Breakout room** | **Importance of buy in and leadership for BR** | **Embedding behavioural research into organisations and structures** | **Prioritisation of BR** |
| --- | --- | --- | --- |
| Academic 1 | None | Question of ‘Are there some sectors where Behavioural research is less dominant?’ Sometimes it is seen as more of an add on rather than being truly integrated, but behavioural science should be underpinning almost everything that we are teaching in some areas. | Academia sometimes sees important areas/questions which are not of interest to policy makers - how can we bridge that gap? Academics sometimes identify small changes which could potentially have big impact, but they do not appear of interest to funders/policy makers because they do not have large scale outcomes. |
| Academic 2 | None | None | None |
| Government 1 | Ministerial appetite for behavioural science varies (between governments but even just individual ministers), but also there were reflections that people have not been resistant to using behavioural science once they know about it. The senior people within a department can affect behavioural research budgets. If they are knowledgeable and in support of behavioural science then budgets are much more stable. Someone needs to have a helicopter view of all the different behavioural science projects that can interlink to see if there are consequences in other areas. | The government rely on outsourcing a lot of behavioural science work. There is an aim to embed behavioural research thinking with social researchers and policy makers to help make more effective and efficient policies. Social researchers are more often embedded in teams. A capability or training programme to build on people’s interest and embed those in sector teams could help improve embedding behavioural research. Behavioural science should be included at the start of policy process. Often people are asked to give advice at the last minute so they are limited in what can be done in the time frame. Teams are working on narrow scope projects and try to have behavioural research at centre of everything. Example: People have letters they want to send out, the team went to recipients and worked out what the barriers were to acting after receiving the letter, worked with the team to put practical things in place to help people, with great success - it was a small programme of work but a big impact. Success is in the way we change culture – getting policy colleagues to think behaviourally. Having research fellows in the team is really valuable and helps with forming links to academia. We need to be in a position to do good behavioural research when its required – e.g. in emergencies – what is needed is evidence but we are not in a place to do the research. Teams are trying to keep behavioural science on the agenda. If behavioural research is embedded into the programme budget then it can be more protected – if it sits centrally then it is more vulnerable. | A lot of the time it comes down to money. Budget restrictions mean that behavioural research is often the first thing that goes. There are cases where behavioural research could have a big impact, but being consultancy-based, cost is very often a barrier. Teams often asked to give advice at the last minute so often limited in what can be done in the time frame. |
| Government 2 | Not having formal leadership in government compromises behavioural research - in Welsh government they are working hard to gain senior leadership buy-in. It requires culture change and can create results people don't want to hear. | COVID created an opportunity to embed itself more broadly, needs to be taken this opportunity. There have been some successes in embedding behavioural research in digital contexts, which may or may not be intentional, and also in communications. Behavioural science as a profession in government has grown in recent years, more interest in GSR and more recognition of it. The professions are examples of where behavioural research is more enmeshed, and can serve as a model for enmeshment compared to in policy groups. Behavioural science is not always as embedded but resources for upskilling can be helpful. Agreement that behavioural science gets more recognition but we need to support policy colleagues more in using it. The challenge is when behavioural research is brought in too late in the process. Whenever policy is in development, there is a checklist that asks science advice in relation to this policy, having a separate box asking about behavioural science advice is a system change which would result in this issue being considered. Secondments by behavioural researchers can create opportunity; useful to have more secondments going on generally. Some government departments provide secondments and opportunities for people to come in a spend a year on particular research questions. People get experience on how organisations function would help change the dial. In Wales mini spi-b created for cross department work and felt it benefitted participants as well learning how government works. Secondment arrangements are not a one-way benefit. Teams are trying to build behavioural science into business as usual in structures that already exist e.g. Apprenticeships. | Behavioural research is often cut first, if there is a potential to have a funding opportunity outside of the organisation the that can be helpful, as well as getting teams to publish successful results. |
| Public&Third 1 | Sometimes senior leadership will not make changes based on behavioural research recommendations. Planning projects also requires multidisciplinary teams, which requires buy-in from multiple stakeholders - there is a question of who pays for this and supports it. There are lots of projects done on tight timeframes, and there is always time to publish results to create visibility. If more funded projects are published, it could create credibility for behavioural research. | Local government funding is low but behavioural research can be more prominent during funding cuts to help make decisions. Council participants have a process internally for behavioural research and this process is videoed/templated to share with others. In policy/ government it can be challenging for behavioural research to feed into fast paced process of decision makers. Going through the behavioural research-to-impact process is a journey and different groups can be successful at different points of the process. There are concerns that behavioural research has become a box tick. | None |
| Public&Third 2 | It is important to get buy-in from leadership. Leadership teams also need an understanding of how behavioural research works, the amount of time and resources required, and that it is not a quick or guaranteed fix. | Most have commissioned work externally, due to limited capacity in-house but with mixed results. Having someone in-post focused on embedding behavioural research in an organisation would help (a “research facilitator”). Some were in the process of developing / just developed research governance process for behavioural research. Examples include i) managing ethics in-house since they got NIH funding to have an embedded research facilitator, ii) a council having a research facilitator, funded through an NIHR call, who has helped build relationships with local Universities and Council ethics team, iii) getting funding to work with a team to develop research skills training for the council, incorporating behavioural science, iv) running behaviour change training for the whole public health team, based on learning gained from a behaviour change course, plus input from the Council’s Insights and Engagement Team and an external behaviour change expert. | Harder to use public funding for behavioural research, and research in general, organisation is focused on doing what they are contractually required to do. |
| Public&Third 3 | Some organisations do not know where to place behavioural science within the organisational structure - if there were more leadership roles in behavioural science, this would help. We need charismatic leadership type roles to elevate the status of behavioural research and to have impact within a policy space, including senior civil servant leadership. It is also important to consider how to increase attendance at behavioural research events. Whilst colleagues may show interest, we need to increase appetite to use behavioural research in one’s work. | Behavioural research is sometimes seen as a ‘nice to have’ – it does not have the profile for it to be a requirement i.e. not built into processes, not seen as necessary. We need training for leaders in how to build behavioural research into strategic planning. Also important to integrate behavioural research to other approaches e.g. quality improvement, implementation. There is little active or proactive work done to integrate behavioural research in teams. | None |
| SMEs 1 | Sometimes clients have specific ideas but will not have behavioural science at the forefront of their mind, so there is a need to help them shift their thinking to align. | Example of a strong behavioural thread that runs through their company - they have someone who drives forward the behavioural economics work. | Local Authorities have to make difficult decisions about where to spend their little amount of funding. Although some clients understand strengths of using a behaviourally led approach to marketing, there are some challenges e.g. budget cuts; almost always the first thing to be cut out, even when they point out this is counterproductive as you are essentially cutting out the strategy. |
| SMEs 2 | None | In projects that are ad-hoc with a quick turn-around, some companies overcome challenges by offering an incentive for interviews with experts or by pay someone as an associate/consultant. | None |
| Cross-sector 1 | There is a desire for leaders to use the research more. If you have a behavioural research/science champion within an organisation it really helps. When using behavioural science, it is not always understood what the benefits are, although some are happy to agree to use behavioural research - it can depend on the type of company. E.g. pharma companies are so used to experimenting and developing a product so are more open to behavioural science and research. A pure sales company may be less interested because they do not have that research background. However even in a design agency with good understanding of behavioural research, there are still creative leads who are resistant. People are excited in the beginning about behavioural research but can be hard to get them to actually use the methods and research. Behavioural science can be daunting if not from that background so demonstrating what value it brings is key. There is a challenge of getting clients to see the importance of taking a certain approach like behavioural research. Co-design is not always popular with creative directors but if they understand behavioural research then it helps. | At the moment behavioural research is embedded at the end of the process but actually would want to embed from the beginning. There are benefits to having core behavioural science team and if you have a behavioural research/science champion within an organisation it really helps. Behavioural research is sometimes more of a tick box exercise. In big companies with huge research teams, often the loudest person in the room gets to make the decisions regardless of evidence/research. | None |
| Cross-sector 2 | When you are a small group with a limited budget you need to make the case for behavioural research, and it can be hard to convince people at a senior level that it is a cost saving exercise. There is an absence of tools and methods and capacity or ability to convince seniors in relation to the benefits of behavioural research, including cost. We need some help in getting the conversation going. There needs to be an impetus from somewhere to convince more senior people in the organisation that there are gains to be made from incorporating BR or that what they are doing is already on the right path. There are also attempts to persuade people to have seniority in behavioural research, that person doesn’t have to be someone from academia. | In agri-environmental, behavioural research been used for a long time (though not always called behavioural research) but they have a good amount of evidence around barriers to behaviours and recognition by government so have employed social researchers in DEFRA and other agencies, though there is still a long way to go. In market and commercial research, approaches like COM-B and other behavioural research techniques have happened under a different name (advertisers have used this for a long time), but it is now starting to be embedded. This has given a language and label to things already being used e.g. using COM-B terms gives it credibility and a shared language, which helps to embed it in commercial research. | None |
