# Supplementary material for: Developing the UK’s capability in behavioural research: Identifying strengths, challenges and strategies through cross-sector workshops
Source: PLoS One. 2026 Sep 10;21(9):e0357909. doi: 10.1371/journal.pone.0357909 (PMC13561315; doi:10.1371/journal.pone.0357909)
Supplement: Supplementary file 4 [file pone.0357909.s004.docx]

# Appendix C: List of detailed strategies from each framework

### Framework 1 Public perceptions and understanding

### 1.1 Create clearer terminology definitions to combat misinformation and reduce confusion about what behavioural research involves.

1.2 Be transparent about the limitations and complexity of behavioural research while protecting nuances in findings.

1.3 Increase accessibility of behavioural research courses and use of social media platforms for promotion.

1.4 Develop new narrative emphasising sustained changes over “magic” solutions while leveraging current relevance to pressing societal issues.

### Framework 2 Communication and translation

### 2.1 Use bite-sized, digestible case studies for busy stakeholders; show clear examples of how behavioural research has been applied so people can see benefits. Case studies should clearly lay out methodology and how research was conducted.

2.2 Use straightforward, accessible language without oversimplifying content; make behavioural research less academic and daunting while maintaining credibility.

2.3 Use social media, LinkedIn, newsletters rather than just academic papers; create small, bite-sized presentations that can be easily shared.

2.4 Use storytelling and narratives to help people make sense of behavioural research.

### Framework 3 Collaboration, networking, and engagement

3.1 Establish mechanisms for networking and engagement across the behavioural research community, including newsletters, forums, conferences, mentorship, and case study sharing to connect practitioners across sectors and avoid duplicating failed approaches.

3.2 Create transdisciplinary roles to bridge gaps between fields and educate sectors on collaboration processes.

3.3 Educate clients about research processes while meaningfully including public involvement and lived experiences.

3.4 Expand networking and engagement events beyond London-centricity and provide accessible online events.

3.5 Create alumni networks, including industry alumni, academic networks and forums, and provide formal networking opportunities at events.

### 4 Research practice and methodology

4.1 Encourage cross-sector learning, e.g. drawing on successful case studies from other sectors (e.g., health) to inform others.

4.2 Develop sector-specific resources and case studies to make behavioural research more accessible and relevant.

4.3 Support training that includes practical application, beyond just theory.

4.4 Allow greater flexibility in applying behavioural frameworks, recognising the need to adapt rather than adopt whole models.

4.5 Use systems mapping as a foundational step before designing interventions.

4.6 Shift away from individual-only behaviour models to frameworks that incorporate group, organisational, or structural influences.

4.7 Invest in organisational-level behavioural science capability, particularly in government and large institutions.

4.8 Support research that examines long-term change across systems, rather than isolated short-term effects.

4.9 Design PhD programmes with embedded collaboration to reduce academic siloing and make research outputs more accessible.

4.10 Create open-access repositories or journals to publish null results and replication studies, reducing duplication, improving learning and encouraging honest reporting.

4.11 Develop AI-powered tools (e.g. a behavioural science search engine) to surface tailored case studies, examples, and insights across sectors.

4.12 Encourage the repurposing of existing datasets to extract behavioural insights cost-effectively.

4.13 Create clear guidance on when and how to use AI tools in behavioural research, particularly for monitoring, synthesis, and insight generation.

4.14 Establish trusted, sector-wide guidance on methodological standards, including ethical evaluation, research governance, and data protection.

4.15 Explore endorsement mechanisms (e.g. a “kite mark”) to help practitioners identify evidence-based, high-quality interventions and resources.

### 5 Behavioural research impact

5.1 Ensure that local authorities and national government are actively involved in behavioural research, as both are essential for shaping effective policy.

5.2 Co-design solutions directly with affected populations and advisory groups of stakeholders.

5.3 Embed behavioural science into standard policy development processes, for instance by instituting a routine “behavioural science check.”

5.4 Establish central “what works” centres to provide policymakers with quick, actionable access to behavioural insights.

5.5 Increase research funding targeted at local authorities and their partnerships to ensure capacity to develop and deliver behavioural interventions.

5.6 Test, compare, and refine interventions in a controlled environment before broader rollout and invest in follow-on phases of research to build momentum beyond pilot studies

5.7 Define impact realistically when working with funders, acknowledging the delayed nature of behavioural outcomes in both design and evaluation timelines.

5.8 Emphasise collective over individual success in systems like the REF.

5.9 Develop systems to identify cross-sector impacts and prevent unintended consequences and develop shared metrics that support long-term evaluation of behavioural change.

5.10 Create targeted resources and frameworks for evaluating impact.

5.11 Reframe behavioural science as a tool for sustainable, long-term change rather than a quick fix.

### 6 Funding, resources, capacity and accessibility

6.1 Create a research funder collective which could provide bigger budgets for large-scale projects.

6.2 Provide behavioural research education for funders and grant reviewers so that expectations of projects are better managed and understood.

6.3 Provide more funding dedicated to supporting cross-sector collaboration.

6.4 Provide top up funds to improve research mid-cycle, allowing for e.g. sharing results with communities and stakeholders.

6.5 Create high quality guidance for conducting and using behavioural research, or developing funding applications for behavioural research, that is user-centred and accessible to non-academic audiences.

6.6 Develop and maintain an active behavioural research repository that stores information from academic papers and uses AI to ask questions about the data.

6.7 Create a central resource bank of training opportunities and interventions and how they work.

### 7 Training and career development

7.1 Provide training on i) influencing policy, including how to operate with policy makers and how to be in the environment alongside policy makers, and ii) how to build networks.

7.2 Ensure that behavioural research training is provided alongside mentorship and support to ensure successful implementation of skills.

7.3 Compile a searchable list of reliable training courses to reduce fragmentation.

7.4 Provide training at different levels of behavioural research expertise, for different career stages and, in government, targeted at specific professions and departments for whom behavioural research is most relevant.

7.5 Training should be provided to leaders who need to consider how to strategically build behavioural research into their organisation.

7.6 Consider how to motivate people to attend training, including senior staff who may be reluctant to join, using methods such as providing certificates.

7.7 Make opportunities for behavioural research roles outside academia, such as in the civil service, more visible.

7.8 Create more opportunities for secondments for behavioural researchers, as these create two-way benefit.

7.9 Create a centralised job board for behavioural research jobs.

7.10 Hiring managers could consider write job descriptions around the skill sets required rather than around titles to find the right applicants.

7.11 Bring behavioural research into school education to improve understanding and inform career choices.

### 8 Leadership, embedding and prioritisation

8.1 Develop a government capability or training programme to build on people’s interest in behavioural research and embed those skills or individuals in teams.

8.2 Whenever government policy is in development, there is a checklist that asks about science advice provided in relation to this policy: suggest adding a separate box asking about behavioural science advice provided.

8.3 Have someone in-post who is focused on embedding behavioural research in an organisation (a “research facilitator”).

8.4 Create formal behavioural science leadership positions to help organisations know where to place behavioural science within their structure and to elevate its status.

8.5 Establish behavioural research champion roles within organisations to help advocate for and support behavioural research integration.

8.6 Provide training and education for leadership teams on how behavioural research works (including realistic expectations about timelines and resources) and how to strategically build it into organisational planning.
