# Supplementary material for: Developing the UK’s capability in behavioural research: Identifying strengths, challenges and strategies through cross-sector workshops
Source: PLoS One. 2026 Sep 10;21(9):e0357909. doi: 10.1371/journal.pone.0357909 (PMC13561315; doi:10.1371/journal.pone.0357909)
Supplement: Supplementary file 5 [file pone.0357909.s005.docx]

# Appendix D: Coding tree showing the correspondence between codes, key themes, and the eight frameworks.

The core analysis team (KS, GY, NH), in calibration with a fourth researcher (NC), agreed on 141 codes during the piloting and indexing phases. During charting, the codes were iteratively reviewed and refined: we combined closely related codes; renamed some to better reflect the content they captured; and removed a small number (n = 14), redistributing their coded content to the best-fitting codes. The refined codes were then grouped into key themes based on conceptual similarity and organised into the eight framework matrices presented in the Results. The table below shows the final coding tree, listing the codes under each key theme and framework.

**Table D1. Coding tree showing the correspondence between codes, key themes, and the eight frameworks**

| **Framework** | **Key theme** | **Codes** |
| --- | --- | --- |
| 1. Public perceptions and understanding | Misconceptions and related challenges | Non-experts' interpretation/perception of BR;  Difficulty understanding BR |
|  | Increased visibility and adoption of behavioural research | Visibility (BR more visible);  A need for BR to be visible;  Visibility (Increased recognition/visibility of beh change);  Selling BR |
|  | Strong interest in behavioural research | Interest in BR;  Public interest in BR;  Increased appeal of behavioural research |
|  | Characteristics of behavioural research | BR is a science;  BR provides strong evidence;  BR is complex / BR can't solve everything;  Strengths in BR do not mean that it is strong;  Missed cultural interpretations in BR;  Standardisation in BR;  BR provides a common language;  BR terms/concepts widely used;  Need for common language;  Lack of consistency within BR;  Value of BR;  BR adds human dimension;  Examples of success with BR;  Urgency of BR applications |
| 2. Communication and translation | Approaches and importance of research communication and translation | Communicating BR for different audiences;  Approaches to research translation;  Use of case studies |
|  | Challenges in communication, translation and implementation | Difficulty translating BR;  Problems with implementation;  Timing of implementation;  Gaps between theory and practice |
| 3. Collaboration, networking, and engagement | Cross-sector collaboration | Importance of collaboration;  Cross sector collaboration;  Cross sector engagement;  Cross-cultural collaboration;  Strong relationships with stakeholders;  Need for PESI;  BR opportunities in industry;  Benefits of multidisciplinarity;  Challenge of multidiscplinarity;  Access to PESI |
|  | Experience sharing, networking and community building | Networking for BR community;  Cross-sector networking;  Supportive community building; Challenges in networking;  Shared goals in BR;  Importance of networks in applying for funding;  Networking opportunities;  Importance of sharing experiences across sectors;  Opportunities to learn from others;  UK-global BR interconnectedness;  UK known internationally to have BR capability;  Strong global community;  Expansion beyond London |
|  | Stakeholder engagement | Managing expectations;  Client expectations;  Funder expectations;  Difficulty engaging with stakeholders;  Difficulty with collaborations;  Inclusion of lived experience |
| 4. Research practice and methodology | Tailoring to contexts and systems thinking | Tailoring to contexts;  Generalisability of BR;  Systems thinking;  Importance of systems thinking;  BR in tech;  Opportunities for AI |
|  | Research best practice (methodological innovation and challenges) | Guidance on research best practice in BR;  Research quality;  Ethics in BR;  Guidance on using AI in BR;  Methodological challenges for BR;  Constraints with academic research; Difficulties in data collection;  Difficulty capturing outcomes/impact;  Methodological innovation;  Methodological flexibility in BR;  Advancing theory development;  Access to data;  Transparency;  Developing research governance;  Research support needed |
| 5. Behavioural research impact | Behavioural research and policy | Importance of policy impact |
|  | Considering impact in behavioural research | Importance of considering impact;  Difficulty measuring impact;  Measuring impact;  Measuring success;  Defining impact;  Long-term thinking |
| 6. Funding, resources, capacity and accessibility | Accessibility of behavioural research | Accessibility of BR |
|  | Central resource bank strategies | Central BR resource bank |
|  | Issue of funding and related challenges | Lack of funding;  Importance of asking for funding;  BR is expensive;  Funder knowledge/awareness;  Reforming funding processes;  Focus on applied research |
|  | Limited capacity (time and human power) | Limited capacity (time & human power);  Limited skills;  Need to build capacity;  Transience issues for capability;  Limited skills (training) |
| 7. Training and career development | Importance of training | Importance of training;  Difficulty training;  Need to build capacity for teaching not just delivering |
|  | Approaches to research training | Approaches to research training;  [Importance of] Ways of teaching/ increasing capability;  Training on policy impact;  Training for networking;  Training in all career stages |
|  | Career routes and progression | BR career routes;  BR career progression;  Training for BR leadership;  Variability of BR usage and careers across departments and organisations;  BR graduates employability;  Diversity of BR researchers;  Certifications in BR;  Confidence of BR researchers;  Define who is a behavioural researcher |
| 8. Leadership, embedding and prioritisation | Leadership and buy-in | BR support from leadership;  Importance of BR leadership;  Organisational leadership in BR;  Buy-in into doing BR;  Reluctance / hesitation to use BR |
|  | Embedding behavioural research into organisations and structures | How BR is embedded;  Embedding beh res ways in processes |
|  | Prioritisation of behavioural research | Issues of prioritisation of BR;  Different BR priorities across sectors;  Pre-agreed beh sci to be responsive to emergencies |

Note: BR = behavioural research. Code labels are reproduced as they were agreed during analysis.
