# Supplementary material for: Developing the UK’s capability in behavioural research: Identifying strengths, challenges and strategies through cross-sector workshops
Source: PLoS One. 2026 Sep 10;21(9):e0357909. doi: 10.1371/journal.pone.0357909 (PMC13561315; doi:10.1371/journal.pone.0357909)
Supplement: Supplementary file 6 [file pone.0357909.s006.docx]

**Major comments**

**1. Methodological rigour and transparency need strengthening**

The manuscript is generally well structured and addresses an important topic. However,the methods section should provide more detail on the analytical process, particularly how the initial codes were generated, how coding consistency was ensured across researchers, how disagreements were resolved, and how the 141 codes were grouped into the eight final frameworks. A coding tree or a table showing the correspondence between codes, subthemes, themes, and final frameworks should be provided, preferably as a supplementary file.

**2. Need to discuss potential biases related to the qualitative workshop methodology**

The study uses online workshops as the main qualitative data collection method. but this approach is vulnerable to several biases that should be explicitly discussed.

The limitations section should include a more explicit discussion of the potential biases associated with qualitative workshop methods, including selection bias, self-selection bias, social desirability bias, group conformity, and dominance effects within breakout rooms. The authors should also explain what measures were taken to mitigate these risks, such as facilitation techniques, inclusive participation strategies, use of chat functions, anonymisation, validation of findings, or triangulation with transcripts.

**3. Clarify how strategies were generated and analysed**

The study first identifies strengths and barriers, then presents ten strategies to strengthen behavioural research capability. However, the methodological pathway from identifying strengths and barriers to generating strategies is not sufficiently clear.

The methods section states that participants discussed strategies during the workshops and that the authors later mapped actor groups with potential responsibility for change. However, the process by which the ten strategy areas were derived, structured, prioritised, or linked to the eight frameworks is not described in enough detail. The results section presents ten overarching strategies, but the analytical logic behind this step could be more systematic. The authors should describe how the ten overarching strategies were derived, how they correspond to the eight frameworks, and whether any prioritisation or validation process was used.

**4. Results section could be more clearly separated from interpretation and discussion**

The results section is rich, but in several places it appears to move beyond description and begins to interpret, explain, or contextualise the findings. In a qualitative paper, some interpretation is expected, but the distinction between **findings**, **interpretation**, and **discussion with literature** should remain clear.

The results section should be reviewed to ensure that it remains primarily focused on reporting findings from the workshops. Explanatory interpretation, comparison with previous studies, and broader implications should be moved to the discussion section where appropriate. The use of references in the results section should also be reconsidered, unless they are necessary to define specific concepts mentioned by participants.

**5. Tables require improvement in readability and presentation**

The tables contain useful information, but their current presentation is dense and difficult to read. Table 1 in particular contains many bullet points, sector abbreviations, and long text entries, which may reduce readability.
