# Supplementary material for: Developing the UK’s capability in behavioural research: Identifying strengths, challenges and strategies through cross-sector workshops
Source: PLoS One. 2026 Sep 10;21(9):e0357909. doi: 10.1371/journal.pone.0357909 (PMC13561315; doi:10.1371/journal.pone.0357909)
Supplement: Supplementary file 7 [file pone.0357909.s007.docx]

Developing the UK’s capability in behavioural research: Identifying strengths, challenges and strategies through cross-sector workshops

Guanyu Yang^1,5*¶^, Katherine R. K. Saunders^1,5¶^, Niamh Hart^2,5^, Joanna Davan Wetton^1,5^, Nia Coupe^3,5^, Isabelle Olson^1^, Lucy Porter ^1,5^, Sharon Cox^4,5^, Jo Hart^3,5^, Susan Michie^1,5^

^1^ Centre for Behaviour Change, University College London, London, United Kingdom

^2^ Usher Institute, University of Edinburgh, United Kingdom

^3^ Division of Medical Education, University of Manchester, United Kingdom

^4^ Department of Behavioural Science and Health, University College London, United Kingdom

^5^ Behavioural Research UK

* Corresponding author

Email: [guanyu.yang.20@ucl.ac.uk](mailto:guanyu.yang.20@ucl.ac.uk) (GY)

^¶^ Guanyu Yang and Katherine R. K. Saunders contributed equally to this work and are joint first authors.

# **Abstract**

Behavioural research shapes policy across multiple sectors; building strong capability for researchers and research users is essential to maximise its impact. This study aimed to identify strengths, barriers, and opportunities for improvement among those conducting or using behavioural research across UK sectors. We conducted five qualitative workshops, with 58 participants from academia, government, public and third sectors, and the private sector. Using framework analysis, growing visibility and successful embedding of behavioural research in some organisations were identified as key strengths. However, barriers included public misconceptions, limited funding and capacity, difficulties in research translation, siloed working, inconsistent training provision, and insufficient leadership support. Participants proposed actionable strategies, including making behavioural research more accessible, reforming funding structures, and enhancing cross-sector collaboration and training. Our findings highlight the need for sustained investment and stronger collaboration to better align behavioural research with public and policy priorities, address sector-specific challenges, and build on existing strengths.

**Keywords:** Behavioural research; Framework analysis; Multi-sector; Workshops; Capability

# **Introduction**

Human behaviour is central to addressing major societal challenges in health, sustainability, education, crime and justice, social welfare, engagement with digital technology and artificial intelligence, and beyond. High quality behavioural research has the potential to significantly strengthen policy and practice in these areas [1–5]. Behavioural research benefits from large-scale collaboration across disciplines and sectors, and explicit links between basic research, applied research, policy and practice [6–8]. However, the spread of behavioural research across many disciplines and sectors also means that behavioural evidence, theory and research methods can be fragmented, weakening the field and limiting its potential to be useful and impactful [8]. Improvements are also needed in scaling up interventions [9–11], safeguarding the ethical use of behavioural science [12–15], and accessing more comprehensive, diverse and high-quality data sources [5,16,17]. Much of the theoretical contributions of behavioural research pertains to processes of change; yet there is a need for more theoretical explanation of variations in behavioural outcomes and of the generalisability of behavioural research findings [18–21].

How these various challenges differ across sectors and disciplines is not clear. We currently lack a comprehensive picture of how well-equipped UK-based organisations are to conduct high-quality behavioural research to meet identified needs in policy and practice, and translate it into impactful outcomes. These organisations usually operate in silos with limited learnings from each other. This study formed part of a comprehensive assessment of UK behavioural research capability conducted by Behavioural Research-UK [22,23]. Building on a documentary review of national and international strategy documents for advancing behavioural research capability [15], organisational network mapping [24] and a UK-wide survey [25], these workshops provided essential qualitative insights into sector-specific challenges and opportunities for strengthening behavioural research capability. In investigating capability, we adopted a broad definition inspired by the capability approach [26]: “the opportunities and conditions that enable people, groups, and institutions, whether they are researchers doing behavioural research or users applying it, to carry out and meaningfully engage with research”. This extends beyond access to resources such as funding and time to encompass the autonomy, expertise, and supportive culture required to translate those resources effectively into impactful contributions.

Recognising the rapid development of behavioural research in recent decades, scholars have reflected on various aspects of research capabilities via narrative review, qualitative, quantitative and mixed-methods design [3,27–30]. While these studies have documented various progress and capability challenges such as funding constraints [28,29] and translation difficulties [31], most focused on specific sectors or contexts. Most existing meta-research concentrates on the producers of behavioural research, as seen in the work of Lecouturier et al. [28], leaving a limited voice for its users. While recent research has examined the collaborative work between researchers and public bodies, it is confined to collaboration on behavioural experiments [30], rather than the broad array of methods used in behavioural research (such as qualitative methods). There are other biases in our knowledge about behavioural research capability building, as although behavioural research encompasses a wide range of academic and professional disciplines, much of the research on capability building originates from the health community [27,31–33]. Relevant studies have primarily investigated behavioural research within health services [31], government behavioural science units [28], or academic psychology departments [34], each providing only partial perspectives on research capability. Thus, we need to develop a multi-sectoral, multi-disciplinary perspective, incorporating views of both researchers and research users.

This study targets researchers, organisational leaders, funders, and training bodies seeking to strengthen behavioural research capability across the UK. By mapping strengths, barriers and strategies for improvement across sectors, we aim to provide these stakeholders with evidence for strategic decision-making and coordinated action. Building on previous phases of the capability scoping study, as described above, we aimed to answer:

1. What are the current strengths of existing behavioural research, including its impact on policy and practice in each sector?
2. What are the barriers to and missed opportunities in conducting and using behavioural research in each sector?
3. In what ways (through what potential strategies) can research capability be strengthened in each sector to facilitate more, better quality behavioural research that meets the needs of both researchers and research users?

# **Methods**

## **Study design**

We conducted five online workshops with UK behavioural researchers and research users across different sectors (Protocol: https://osf.io/r2fhv/overview). Workshops are a well-established qualitative method for convening diverse stakeholders to collaboratively generate strategic insights by encouraging group creativity and deliberation [35–37]. Although traditionally conducted face-to-face, the shift to online formats post-COVID-19 has provided a convenient, flexible and inclusive option for convening stakeholders across geographic boundaries [38–41]. This study received ethical approval from the UCL Research Ethics Committee (27193/001) and was reported in accordance with the Standards for Reporting Qualitative Research (SRQR) guidelines (See S1 File for checklist) [42].

## **Participant recruitment and sampling**

### **Recruitment**

We organised workshops by sector; academia, government, public and third sector, and small and medium-sized enterprises (SMEs), to capture sector-specific perspectives on behavioural research capability. We primarily identified participants from the pool of respondents to the BR-UK Cross-Sector Survey who had consented to follow-up contact [25]. If potential participants did not clearly fall into one sector, we allocated them to a workshop based on which was most aligned with their role. Government participants were invited via a cross-government behavioural science network. Participants were offered a voucher for their participation at a rate of £10 per hour up to £30. Government participants were not permitted to take payment. All participants were offered support funds for attendance/access costs. Participants were recruited between 15/10/2024 and 06/12/2024.

To be eligible for inclusion, participants were required to (1) meet the definition of a behavioural researcher or behavioural research user as described previously [43]; (2) work in a relevant organisation, department, or on topics relevant to the focus of the workshop; and (3) work in the UK. We excluded co-investigators or researchers within our own research consortium (BR-UK), as well as start-up or scale-up employees, since workshops tailored for this latter group were conducted in a separate study by a partner organisation [44].

### **Sampling**

We used purposive sampling to ensure representation across research topics, seniority levels, geographical locations, and personal characteristics. We held a final “cross-sector” workshop to convene voices missing or underrepresented in the previous four workshops, as observed by the research team and identified by previous workshop participants via the Equality, Diversity, Inclusion and Intersectionality (EDII) follow-up questionnaire.

## **Workshop procedure and data collection**

We conducted five online workshops (2.5 hours each) between November and December 2024 on Microsoft Teams: academia (n=14), public/third sector (n=13), SMEs (n=9), government (n=9), and cross-sector (n=13). Due to low response levels from larger private sector organisations, participants in this group were invited to the cross-sector workshop. All participants provided written informed consent. We implemented measures to ensure inclusive participation [45], including offering access funds, holding sessions during core working hours, and enabling multiple forms of participation (e.g., chat and microphone).

Participants attended an opening plenary session before being divided into 2-3 breakout rooms per workshop, with 11 breakout rooms in total, each with a facilitator and note-taker. Facilitators and note-takers had experience in qualitative research methods and received briefing on the study protocol. Workshops followed a structured format with three facilitated group discussions in breakout rooms: (1) current strengths and examples of successes in behavioural research (approx. 20 minutes); (2) challenges and missed opportunities (approx. 20 minutes); and (3) strategies for capability improvement (approx. 40 minutes). Discussions were conducted under Chatham House Rules [46]. Workshops were recorded and transcribed, with detailed notes taken during discussions. Notes were anonymised and compiled as the primary data source for timely and efficient analysis considering the large amounts of parallel discussion data, with transcripts used to verify and illustrate key points as needed. Workshop materials to guide discussions and anonymised facilitator notes are available on Open Science Framework (See Data Availability Statement), with more detailed workshop procedure available in S2 File (Appendix A).

## **Data analysis**

Workshop data were analysed using framework analysis [47], a systematic method suitable for multi-participant workshop data where researchers can compare perspectives both within and across cases. As an iterative approach, it involved familiarising with data, coding, developing initial frameworks, charting data into matrices, and interpretation. We produced a matrix output with cases (breakout rooms in our study) as rows, and themes as columns, enabling us to examine patterns across sectors while maintaining the ability to trace findings back to specific workshop contexts [48,49]. Each matrix is referred to as a “framework”.

#### **Familiarisation and initial coding**

The core analysis team of three researchers familiarised themselves with the data. The analytical approach was piloted with a subset of notes from three selected breakout rooms. It became clear that participants discussed strengths, barriers and strategies across all three breakout room activities and developed conversations around key topics of interest to them, which were important to represent in the framework. Therefore, indexing and charting data strictly according to the key research questions (strengths, barriers and strategies) would not capture nuanced discussions and emergent themes, and we therefore adapted our approach.

#### **Indexing**

Taking a more inductive approach, the core analysis team iteratively coded the remainder of the notes, using agreed codes from the initial coding phase and creating new ones as necessary. These were iteratively reviewed and refined, resulting in 141 codes.

#### **Charting**

The indexed notes were structured into framework matrices using Microsoft Excel. The 141 codes were grouped into umbrella themes based on conceptual similarity and then were further organised into eight separate framework matrices based on thematic coherence. Areas of ambiguity or overlap between frameworks were reviewed and resolved through team discussion.

#### **Interpretation**

With data organised across eight thematic frameworks, which are presented in the results section of this paper, we analysed patterns both within and across sectors in line with our research questions. We identified challenges and strengths across and within sectors, and examined how proposed strategies varied, or were consistent, by sector. We narratively summarised each framework and tabulated key strategies. To inform the implementation of these strategies, we mapped actor groups with potential agency and responsibility to affect change, which was refined through consultation with our internal team. Workshop participants were invited to review and validate the draft results in July 2025 to ensure accuracy of the interpretation.

# **Results**

## **Strengths and barriers to strengthening behavioural research capability**

The workshops identified a range of strengths and barriers to building behavioural research capability across the UK. The eight frameworks developed through the analysis process centre around eight core themes are: (1) public perceptions and understanding, (2) communication and translation, (3) collaboration, networking and engagement, (4) research practice and methodology, (5) behavioural research impact, (6) funding, resources, capacity and accessibility, (7) training and career development, and (8) leadership, embedding and prioritisation. While these frameworks are presented separately for focused analysis, they are connected and overlapping in nature. A summary of the strengths and barriers across each of these central themes can be found in Table 1 and subsequent narrative summaries, with complete frameworks available in S3 File (Appendix B).

**Table 1. Summary of strengths and barriers in behavioural research capability identified across eight frameworks.**

| **Framework** | **Strengths** | **Sectors^1^** | **Barriers** | **Sectors^1^** |
| --- | --- | --- | --- | --- |
| **1. Public perceptions and understanding** | - Growing visibility and adoption | - All | - Oversimplification of behavioural research as just “nudging” | - G, S |
|  | - Use of common language | - All | - View of behavioural research as “social engineering” or manipulation | - G, P |
|  | - Widely used theories and frameworks | - A, G, C | - Confusion around terminologies and technical processes | - P, S, C |
|  | - Solid evidence base | - G, S, P, C | - Misconception that everyone understands human behaviour | - S, C |
| **2. Communication and translation** | - Successful cases of communication and translation | - P, S, C | - Difficulty in translating research outputs | - All |
|  |  |  | - Difficulty in bringing theory and research into action and practice | - A, P, S, C |
| **3. Collaboration, networking, and engagement** | - Existing effective collaborative and knowledge-sharing mechanisms | - All | - Academics working in silos | - A, P, S |
|  |  |  | - Lack of connection and linkage across sectors | - G, P, S |
|  |  |  | - Lack of trust and understanding | - P, S, C |
|  |  |  | - Low visibility of ongoing work | - P, S |
|  |  |  | - Difficulty in attending networking events | - A, S, C |
|  |  |  | - Difficulty in managing expectations | - A, P, S |
| **4. Research practice and methodology** | - Established ability to tailor to contexts | - A, P, C | - Difficulty in tailoring to complex contexts/translation | - All |
|  | - Progress in applying systems thinking | - A, G | - Still a heavy focus on individual behaviour and lack of accounting for context | - A, G, P |
|  | - Growing momentum in methodological innovation and ethical awareness; and upholding methodological standards | - G, S, C | - Continued inconsistency of methodological and ethical standards across behavioural research | - A, G, P |
|  |  |  | - Difficulty in capturing outcomes | - A, G, P, C |
|  |  |  | - Tensions between methodological ideals and real-world constraints | - A, S, C |
|  |  |  | - Impactful practice unintentionally affected by academic norms | - P, S |
|  |  |  | - Undervaluing of qualitative research | - C |
|  |  |  | - Concerns over data quality and participant integrity | - G, C |
| **5. Behavioural research impact** | - Demonstrated value in strengthening policy systems with a shift from general awareness to real-world impactful application | - G, S | - Difficulty in influencing policymakers | - A, G |
|  |  |  | - Constraint in capacity and time for meaningful outcomes | - G, P, S |
|  |  |  | - Difficulty in evaluating policy impact | - C, G, P |
|  |  |  | - Knock-on effects of policy intervention in other domains | - A, G |
|  |  |  | - Demand for clear, short-term results versus impact revealed over time | - P, G |
|  |  |  | - Short-term funding cycles | - P, S |
| **6. Funding, resources, capacity and accessibility** | None | None | - Lack of long-term, stable funding | - A, G, P, S |
|  |  |  | - Limited time, resource, and/or expert knowledge | - A, G, P, C |
|  |  |  | - Difficulty in accessing academic journal articles | - G |
| **7. Training and career development** | - A variety of established training provided across sectors | - S, C | - Difficulty in identifying suitable training opportunities | - G, C |
|  | - A new generation of behavioural researchers with more career growth and options | - P, S, C | - Limited resources for training | - G, P |
|  |  |  | - Difficulty in breaking into the industry and government | - A, G, S |
|  |  |  | - Difficulty in defining researcher identity | - C, P |
| **8. Leadership, embedding and prioritisation** | - Leadership/client appetite for behavioural research is good/improving | - G, C | - Lack of knowledge and understanding of behavioural research of senior leaders and stakeholders | - P, C |
|  | - Successful cases of embedding behavioural research | - G, P, S, C | - Lack of behavioural research integration/embedment in the research process | - A, G, P, C |
|  |  |  | - Difficulty in placing behavioural research within organisations without leadership as champions | - S, C |
|  |  |  | - Lack of interest from decision-makers | - A, P |

Note: ^1^ The Sector(s) columns denote the sector-specific workshops where the respective points of strengths or barriers were identified. “All” refers to all sector workshops: “A” = academic, “G” = government, “P” = public & third-sector, “S” = Small and medium enterprises, and “C” = cross-sector.

### **Framework 1: Public perceptions and understanding**

The four key themes within this framework are: 1) characteristics of behavioural research, 2) strong interest in behavioural research, 3) increased visibility and adoption of behavioural research, and 4) misconceptions and related challenges. Consensus across sectors suggests behavioural research has achieved significantly increased and improved visibility and adoption, expanding beyond health and environmental research areas. Other key strengths included frameworks like COM-B [50] providing a common language for collaboration and a robust, highly regarded evidence base (particularly in health applications, e.g., smoking cessation) delivering scientific credibility that appeals to many audiences. However, fundamental misconceptions persist across sectors. Stakeholders may oversimplify behavioural research as “nudging”, dismiss research or interventions as manipulation, or confuse terminology. The pervasive belief that everyone inherently understands human behaviour leads to resistance to evidence-based approaches.

### **Framework 2: Communication and translation**

The two key themes in this framework are: 1) approaches to and importance of research communication and translation and 2) challenges in communication, translation and implementation. Examples of communication and translation successes included the use of evidence-based advisory groups throughout a project on organ donation in Northern Ireland, the development of a sexual health website for young people that resulted in increased bookings for sexual health screenings, and charities optimising email language to focus on enabling recipients rather than appearing manipulative. A major challenge was translating complex behavioural research in an accessible way without compromising scientific rigour, with cross-sector participants recognising difficulties in explaining methods to external audiences. There were also significant time constraints for reading and digesting long reports, highlighting the need for succinct summaries. Government participants noted their continuing struggle to translate policy problems into behavioural research and vice versa, making it challenging to meet the specific needs of policy colleagues.

### **Framework 3: Collaboration, networking, and engagement**

The three key themes in this framework are: 1) cross-sector collaboration, 2) experience sharing, networking and community building, and 3) stakeholder engagement. There were successes in the development of cross-sector collaboration models: for instance, the Competition and Markets Authority’s adoption of choice architecture language enabled lawyers and economists to work together effectively. Experience-sharing systems included the Welsh government’s community of practice and cross-government behavioural insights networks, with SME participants describing a “strong, tight-knit global community” that positions the UK as optimal for behavioural research. Barriers included researchers working in silos, with government and SME discussions further highlighting the disconnect between academia, the third sector, and government. Mismatches in stakeholder expectations were reported across workshops, with demands for quick results and large-scale outcomes conflicting with the small changes typical of effective behavioural interventions. Experience sharing was limited by a lack of visibility of ongoing, external work and networking difficulties caused by costly, often London-centric events.

### **Framework 4: Research practice and methodology**

The two key themes within this framework are: 1) tailoring to contexts and systems thinking and 2) research best practices. Participants highlighted the ability to tailor behavioural research to contexts as a key strength. Cross-sector participants emphasised how flexible approaches enabled safer research engagement, such as remote participation for vulnerable populations, while public and third sector participants shared examples of adapting behavioural frameworks through bespoke internal training. SME participants also valued the strong ethical practices underpinned by the UK’s robust regulatory frameworks (i.e., the Financial Conduct Authority and the Competition and Markets Authority), which provide credibility. Key methodological challenges included a lack of systems thinking, with government participants warning of the potential for unintended knock-on effects across sectors and populations when behavioural research or interventions focus solely on individual behaviour, without consideration of context. Cross-sector participants also noted gaps between behavioural science training and practical application, while SME participants highlighted tensions between the for real-world testing and client demands for controlled evidence upfront.

### **Framework 5: Behavioural research impact**

The two key themes in this framework are: 1) considering impact in behavioural research and 2) behavioural research and policy. In terms of strengths, government participants highlighted the value of behavioural research in strengthening policymaking systems. Specifically, they noted that behavioural research enhances accountability, helps to clarify underlying problems, and prompts policymakers to consider the unintended consequences of proposed actions. Government participants also cited the successful application of insights from behavioural research during the COVID-19 pandemic as a good example of evidence-based policymaking, prompting questions about how similar approaches could be embedded in future crisis responses. Regarding barriers, academic participants reflected on their difficulties in influencing decision-makers, which was supported by government participants who emphasised that political realities may prevent implementation even when evidence is robust, thus limiting impact. Concerns across government and public and third sector groups also included demands for short-term results despite the long-term and non-linear nature of behaviour change.

### **Framework 6: Funding, resources, capacity and accessibility**

The four key themes in this framework are: 1) accessibility of behavioural research and related resources, 2) central resource bank strategies, 3) issue of funding and related challenges, and 4) limited capacity and resources. No strengths were identified, with the discussion concentrating on barriers. Participants from all sectors cited lack of long-term stable funding as a key barrier and felt that funders do not understand the costs involved in behavioural research and that impact can be difficult to demonstrate in short-term funding cycles. Both SME and academic participants desired more funding for longer-term collaborations. Government and cross-sector workshops noted behavioural research is often cut first during budget reductions, despite being core to the research strategy. Government teams also often lost expertise through staff turnover, leading to outsourcing of research. Lack of accessibility to resources was seen as a significant barrier to conducting and using behavioural research across sectors, for example, government participants noted that they are unable to access and make use of work contained within academic journals.

### **Framework 7: Training and career development**

The three key themes in this framework are: 1) importance of, need for and barriers to training, 2) examples of and potential approaches to research training, and 3) career routes and researcher confidence. Participants shared examples of effective training approaches, both in terms of providing or attending behavioural research training or using behavioural research techniques in delivering training. Examples from academia and the public and third sector respectively included i) using the Behaviour Change Wheel framework and COM-B model as teaching tools and ii) developing in-house training on social sciences and behavioural science frameworks. Participants also reported that behavioural research had increasing career options, with SME participants seeing behavioural science as an outlet for psychology graduates. Challenges included difficulty identifying opportunities outside academia, gaps in high-quality, consistent training provision, and limited external training budgets. Career barriers outside academia included difficulties in breaking into industry and low behavioural research presence in government, making it harder to attract quality researchers. In addition, public and third sector participants questioned what qualifications or experiences are required to claim the title of ‘behavioural researcher’. Some described feeling fraudulent identifying as behavioural researchers without formal training.

### **Framework 8: Leadership, embedding and prioritisation**

The three themes in this framework were 1) importance of leadership and ownership of engagement, i.e. buy-in, 2) embedding behavioural research into organisations and structures, and 3) prioritisation of behavioural research. Strengths included an increased appetite in government for behavioural research despite variations between governments and individual ministers, and the growth of behavioural science as a profession. Elsewhere, it was noted that behavioural research approaches, such as the COM-B model, have long been used (in markets and commercial research, for example) but not cited as such, and these techniques are now starting to be more clearly embedded. Challenges include limited impact due to behavioural research being considered at the last-minute, clients feeling excited about behavioural research initially but then failing to translate this into longer-term engagement, and requiring demonstration of cost savings to senior leaders who lack understanding of time and resource requirements. Public and third sector participants felt that charismatic leadership was needed to elevate the status of behavioural research.

## **Strategies to strengthen research capability**

Across the workshops, participants suggested and discussed strategies by which behavioural research capability could be improved in their sector and/or area of expertise. The strategies centre around ten overarching approaches to improving capability: 1) Creating accessible communication resources, 2) Establishing a central repository/resource bank, 3) Embedding behavioural science into policy and research processes, 4) Building multi-level training programmes, 5) Reforming funding structures, 6) Establishing behavioural research leadership and champions, 7) Strengthening stakeholder and public engagement, 8) Creating transdisciplinary roles and networks, 9) Shifting to systems-level approaches, and 10) Establishing quality standards and accessible guidance. Key strategies and examples can be found in Table 2, alongside supporting quotes from the workshops and key actors (individual and organisational) to implement each strategy. We used the notes from note-takers as the primary data source for the framework analysis. The example quotes presented in this table were extracted from the workshop transcripts for the purpose of illustration and to provide contextual detail. A complete list of strategies is available in S4 File (Appendix C).

**Table 2.** **Key strategies identified in workshops for capability building in behavioural research.**

| **Key strategies** | **Example participant quotes** | **Corresponding framework(s)** | **Key actors** |
| --- | --- | --- | --- |
| **1. Create accessible communication resources**   - Provide plain language summaries alongside technical details - Use bite-sized case studies with clear methodology - Use multiple channels (social media, newsletters, podcasts, and school education) to reach a diverse audience - Use storytelling and narratives | *“We’re coming to talk about behavioural science [...] and even words like “heuristic” can be a bit of a sort of a bridge too far. We're trying to [...] not dim it down, but communicate in a way which is not intimidating and therefore be easy to do business with.”* (SME)  *“I know my colleagues wouldn't have time to look at that [research papers], so seeing kind of bite size [...] case studies and then possibly kind of webinars explaining those things in more detail just makes it that much more kind of accessible and relatable.”* (Public & Third Sector)  *“People are interested in stories […] It could be a podcast series where it's like a limited time or I don't know, guest features on things and just getting it out there. […] It's storytelling which is important that I think people will take up.”* (Cross-sector) | Public perceptions and understanding;  Communication and translation;  Collaboration, networking, and engagement;  Training and career development | Researchers;  Research community/networks |
| 2. **Establish a central repository/resource bank**   - Create searchable databases of interventions, case studies, and evidence - Include failings, learnings, null results and replications - Provide AI-powered search functions to find tailored cases - Include job boards and career opportunities | *“[A] repository of knowledge, that would be great [...] one that's more aligned with AI would be great too.”* (SME)  *“It would have certainly been useful to us at the beginning of our programme - a repository of case studies on previous behavioural science programmes. How they were implemented, what worked, what didn't.”* (Government) | Research practice and methodology;  Behavioural research impact;  Funding, resources, and capacity;  Training and career development | Researchers;  Research community/networks;  Universities/research institutions |
| **3. Embed behavioural science into policy and research processes**   - Add behavioural science to policy development checklists - Position behavioural research earlier in decision-making - Invest in organisational-level capability, including formal facilitating roles - Create secondment opportunities | *“In government departments, I know in my case there's a checklist which currently asks ‘what is the science advice in relation to this policy and development?’. And I think to have a separate box or checklist which says ‘is there a behavioural science advice?’ [...] is a system change that at least ensures that the issues will be considered, providing that the appropriate expertise exists and is accessible.”* (Government)  *“Embedding behavioural researchers in government is important [...] if somebody is present then they're more likely to be able to influence policy”* (Government) | Research practice and methodology  Behavioural research impact;  Leadership, embedding and prioritisation;  Training and career development | Universities/research institutions;  National/local governments;  International organisations;  Industry partners (co-producing research);  Research user organisations (including public, private, third sector, applying research) |
| **4. Build multi-level training programmes**   - Provide training at different levels (introduction to advanced) - Include mentorship and implementation support - Build a searchable list of training opportunities | *“It would be really helpful to have training and guidance that's kind of tailored to different audiences [...] There's people like us that are doing behavioural research that may have more advanced skills, but a lot of the people that we're supporting are in public health roles or health and social care [...] they do want to kind of draw on people research and undertake it themselves, but often are scared by [...] the level of robustness required.”* (Public & Third Sector) | Research practice and methodology;  Funding, resources, and capacity  Training and career development;  Leadership, embedding and prioritisation | Universities/research institutions;  Research user organisations (including public, private, third sector, applying research) |
| **5. Reform funding structures**   - Create funder collectives for larger projects - Target local authority partnerships - Support cross-sector collaboration - Define impact and success realistically when working with funders | *“Behavioural research isn't always cheap, and it shouldn't be cheap. If we want to understand behaviour fully, you need money to do it [...] Research fellows cost money.”* (Academic)  *“One thing that was identified across [funding] applications is that we often get comments about the cost of our projects [...] I'm sorry for being alive and costing money, right? But this is often how I feel.”* (Academic)  *“People are kind of our resource. They're our Large Hadron Collider and they are expensive.”* (Academic) | Behavioural research impact;  Funding, resources, and capacity | Research funders |
| **6. Establish behavioural research leadership and champions**   - Create formal supportive leadership positions within organisations - Identify and support organisational champions - Train leaders on strategic planning and realistic expectations | *“I'd like to see changes [...] in the higher, higher leadership. There just seems to be not a clear understanding of what behavioural science is and how it can help so oftentimes [...] we get asked to contribute to things and we’re thought of as like a magic bullet. And that way if you get us involved, we'll fix it [...] it's not how we work at all.”* (Public & Third sector)  *“We've had very senior champions for behavioural science [...] so that kind of bypasses [...] the need for funding issues to be discussed because it's so obvious to them how important this stuff is. At a senior level, how one can ensure that senior people are behind behavioural science?”* (Government) | Public perceptions and understanding;  Leadership, embedding and prioritisation | Researchers;  Research users/practitioners;  Research community/networks;  Universities/research institutions;  Research funders;  National/local governments;  International organisations;  Industry partners (co-producing research);  Research user organisations (including public, private, third sector, applying research);  Publishers |
| **7. Strengthen stakeholder and public engagement**   - Co-design solutions with affected populations and advisory groups - Educate clients and stakeholders about research processes - Ensure local and national government involvement in research for policy impact | *“There's a need to have a conversation around the different stakeholders [...] for example, another sector might see a nudge differently from sectors such as marketing, for example.”* (SME) | Collaboration, networking, and engagement;  Behavioural research impact | Researchers;  Research users/practitioners;  National/local governments;  International organisations;  Industry partners (co-producing research);  Research user organisations (including public, private, third sector, applying research) |
| **8. Create transdisciplinary roles and networks**   - Bridge gaps between sectors and disciplines - Facilitate cross-sector learning and collaboration - Establish networking mechanisms (forums, conferences, mentorship) - Ensure geographic accessibility through online and regional events | *“More opportunities to bridge the gap between the fields would be great... also just improving networks for collaboration between like researchers, NGOs.”* (Public & Third Sector) | Collaboration, networking, and engagement;  Research practice and methodology | Research community/networks;  Universities/research institutions;  International organisations;  Industry partners (co-producing research);  Research user organisations (including public, private, third sector, applying research) |
| **9. Shift to systems-level approaches**   - Move beyond individual behaviour models - Use systems mapping as a foundation - Focus on long-term and sustainable change | *“Now we’re really interested in how you apply systems thinking in particular to creating sustainable behaviour change, because we recognise there’s a need for that in particular.”* (Academic) | Research practice and methodology;  Behavioural research impact | Researchers;  Research users/practitioners; Research community/networks;  Industry partners (co-producing research);  Research user organisations (including public, private, third sector, applying research) |
| **10. Establish quality standards and accessible guidance**   - Clear definitions and terminology - Methodological standards - Sector-specific guidance | *“We've been able to kind of lead on behavioural science or behavioural research, but in those circumstances it would be good to have sort of something to lean on external to the organisation […] like the NICE guidelines, you know, shape how healthcare happens and they're referred to.*  *I almost visualise something a bit like that, but a behavioural science lens [...] that is able to really advocate for behavioural research.”* (Public & Third Sector) | Public perceptions and understanding;  Research practice and methodology  Funding, resources, and capacity | Research community/networks;  Universities/research institutions;  International organisations;  Publishers |

# **Discussion**

## **Overview of key findings**

Our study identified current strengths, barriers to and missed opportunities in conducting and using behavioural research across sectors, and potential strategies which can strengthen research capability across sectors. Strengths included cross-sector increased visibility and institutional presence across the UK, perceived credibility rooted in a growing robust evidence base (particularly in health), successful applications in policy and practice such as during COVID-19, and strong collaborative and methodological innovation. Concerning the barriers and missed opportunities, the workshops revealed persistent misconceptions about behavioural research, siloed working, and challenges in translating evidence into practice. Cross-sector issues such as short-term funding, capacity limitations, and a lack of training were consistently reported, in addition to institutional resistance and a lack of leadership to sustain long-term behavioural research practices. Participants proposed ten priority strategy areas, including improving behavioural research accessibility and establishing central resources, structural embedment of behavioural research in research processes, and improving leadership engagement. The cross-sector workshop produced findings that were largely in agreement with our sector-specific workshop, emphasising the cross-sector nature of many of the strengths and challenges and the mutual need for collaboration and resource sharing.

## **Comparison with existing literature**

Our study found that behavioural research in the UK has gained significant public visibility and understanding over the last few years, which mirrors the global pattern [29]. Behavioural research has also taken root in an increasing number of UK organisations with dedicated teams being set up [3], indicating wider knowledge and acknowledgement of behavioural research. Our study also identified several successful practices in translating research into practice and existing networking mechanisms, reflecting strong collaborative relationships and knowledge exchange between researchers and stakeholders [28]. Behavioural research was seen as having an impact on policymaking, for instance, during the COVID-19 pandemic, and in private-sector applications, consistent with other findings [3,28].

No strengths were mentioned regarding funding, resources, and capacity, suggesting a lack of supportive resources and capacity in behavioural research, which is reflected also in the wider literature [15,31,34]. We identified gaps between research and practical application, with research silos and limited cross-sector connections constraining collaboration. This research-practice gap is consistently identified across the literature [15,28–30]. For example, policymakers often demand quick, conclusive results while researchers require time for rigorous evaluation; this short-term approach has been widely discussed (e.g. Sanders et al. [3]). Furthermore, participants’ call for systems-level approaches aligns with growing international recognition that individual behaviour change interventions face inherent limitations when structural constraints are present [15,51].

The ten strategies that emerged from workshops provide targeted responses to the barriers identified above. Creating accessible communication resources and central repositories directly addresses both the persistent translation gaps between research and practice [28,30], as well as public misconceptions about behavioural research. The emphasis on embedding behavioural science into policy processes and establishing formal leadership positions and champions responds to the institutional resistance and marginalisation challenges that prevent sustained impact [15,25]. Multi-level training programmes can address the fragmented and inconsistent training provision and the identity crisis facing behavioural researchers [25]. Transdisciplinary networks and roles specifically address siloed working and limited cross-sector connections. Meanwhile, strengthened stakeholder engagement will help manage the mismatched expectations between researchers who require rigorous evaluation and policymakers and industrial clients who demand quick results, as found in previous studies [44]. Notably, what distinguishes these strategies from recommendations in prior work is their specificity regarding implementation mechanisms: participants proposed concrete tools, such as formal facilitating roles, specific funding collective models and behavioural science checklists, to supplement abstract recommendations with more actionable steps.

## **Strengths and limitations**

This study benefitted from using workshops for data collection and the inductive approach of framework analysis, which allowed for easy stratification and synthesis of data from each workshop and across sectors and themes. The workshop guide was designed to generate actionable insights, resulting in the collection of concrete strategies for improving behavioural research to inform capability-building efforts. We gathered participants from multiple sectors, providing a rich mix of perspectives and diverse insights. The piloting of the analytical approach, using a purposefully selected small number of workshop notes and frequent meetings for calibration, ensured the team used a harmonised approach in coding the notes. Holding a final workshop for missing voices helped to capture additional perspectives. The analytical approach chosen also enabled us to show that the cross-sector workshop essentially confirming rather than contradicting sector-specific findings. While some topics were unique to specific sectors, core issues such as funding, translation, and collaboration appeared consistently across workshops. This study was limited by the potential for self-selection sampling bias as those already invested in increasing behavioural research capability may have agreed to participate, and the primary pool for invitees consisted of those who had already engaged with BR-UK in their initial survey, making them a particularly research-engaged group. Secondly, due to resource constraints, we primarily analysed workshop notes rather than transcripts verbatim, so there is the potential for some missed nuances of the discussions.

## **Implications for policy and practice**

This study has gathered and thematically organised key, actionable strategies suggested by behavioural researchers and behavioural research users from across sectors and topic areas. For example, government departments could benefit from making behavioural research roles in the civil service more visible and create more opportunities for secondments for behavioural researchers into government, to facilitate better research-policy translation. It would be beneficial for research funders to commit to increased, long-term funding for cross-sector multi-disciplinary collaboration, addressing the critical funding barriers identified across all sectors. This includes establishing longer funding cycles and creating funder collectives for large-scale initiatives. Training bodies and research institutions could helpfully develop a central resource bank of high-quality behavioural research examples and training, along with sector-wide methodological standards and guidelines, particularly relating to data protection and AI use. Organisational leaders across sectors could considering embedding behavioural research into organisational decision-making processes and establishing formal champion roles to address the leadership gap.

## **Implications for research**

Research should continue to prioritise a systems approach which is vital to ensure that behavioural research has the capability of being effective over the long term in addressing community and societal challenges. Behavioural research teams should consider including key stakeholders from the outset of projects and make efforts to effectively translate findings for their target audience. Learnings from high-impact research case studies should be highlighted and shared in keeping with open-science principles, in order to improve practice and support lesson sharing outside of academia. Where behavioural research forms a component of a broader, multifaceted project, it should be considered from the beginning and built into the overall project strategy.

# **Conclusion**

This study provides a multi-sector examination of behavioural research capability across the UK, revealing a field with significant visibility and institutional recognition but fundamental challenges that threaten its current and sustained impact. Our findings outline strategic priorities crucial for advancing the discipline and provide a comprehensive blueprint for coordinated action across stakeholder groups, from developing systems thinking capabilities among researchers to restructuring funding approaches for decision-makers. Only through coordinated investment in behavioural research infrastructure can we transform fragmented efforts into cohesive, transparent, evidence-based practice with greater societal impact.

# **Acknowledgments**

We would like to acknowledge the contributions of Julius Alejandre, Maria Fomina, Milly Massoura, Rosie Webster, and Lisa Zhang to the delivery of the sector workshops. We used AI-assisted tools (Claude Sonnet 4.5) to improve the language and clarity of this manuscript. All content was written, verified, and approved by the authors.

# **References**

1.	Cheung R, Ardolino A. Behavioural science in public health policy. Br J Healthc Manag. 2011;17: 140–144. doi:10.12968/bjhc.2011.17.4.140

2.	Chetty R. Behavioral Economics and Public Policy: A Pragmatic Perspective. Am Econ Rev. 2015;105: 1–33. doi:10.1257/aer.p20151108

3.	Sanders M, Snijders V, Hallsworth M. Behavioural science and policy: where are we now and where are we going? Behav Public Policy. 2018;2: 144–167. doi:10.1017/bpp.2018.17

4.	West R, Michie S, Chadwick P, Atkins L, Lorencatto F. Achieving Behaviour Change: A Guide for National Government and Partners. PHE publications; 2019. Available: https://assets.publishing.service.gov.uk/government/uploads/system/uploads/attachment_data/file/933328/UFG_National_Guide_v04.00__1___1_.pdf

5.	Hallsworth M. A manifesto for applying behavioural science. Nat Hum Behav. 2023;7: 310–322. doi:10.1038/s41562-023-01555-3

6.	Sumner JA, Carey RN, Michie S, Johnston M, Edmondson D, Davidson KW. Using Rigorous Methods to Advance Behaviour Change Science. Nat Hum Behav. 2018;2: 797–799. doi:10.1038/s41562-018-0471-8

7.	Flinders M. Fit for the Future: Research Leadership Matters. Economic and Social Research Council; 2020.

8.	Box-Steffensmeier JM, Burgess J, Corbetta M, Crawford K, Duflo E, Fogarty L, et al. The future of human behaviour research. Nat Hum Behav. 2022;6: 15–24. doi:10.1038/s41562-021-01275-6

9.	Indig D, Lee K, Grunseit A, Milat A, Bauman A. Pathways for scaling up public health interventions. BMC Public Health. 2017;18: 68. doi:10.1186/s12889-017-4572-5

10.	Beets MW, von Klinggraeff L, Weaver RG, Armstrong B, Burkart S. Small studies, big decisions: the role of pilot/feasibility studies in incremental science and premature scale-up of behavioral interventions. Pilot Feasibility Stud. 2021;7: 173. doi:10.1186/s40814-021-00909-w

11.	Saeri A, Slattery P, Tear MJ, Varazzani C. Scale up of behaviour change interventions: A rapid review of evidence and practice | Request PDF. Open Science Framework; 2021. doi:10.31219/osf.io/scd3k

12.	Lepenies R, Małecka M. The ethics of behavioural public policy. The Routledge Handbook of Ethics and Public Policy. Routledge; 2018.

13.	Kuehnhanss CR. The challenges of behavioural insights for effective policy design. Policy Soc. 2019;38: 14–40. doi:10.1080/14494035.2018.1511188

14.	UNICEF Innocenti Research Centre, Tindall K, Hayward L, Hunt E, Hickler B. Ethical Considerations When Applying Behavioural Science in Projects Focused on Children. 2022 [cited 22 July 2025]. doi:10.18356/25211110-2021/03

15.	Hart N, Davan Wetton J, Coupe N, Bauld L, Alejandre JC, Michie S. Current and future priorities for UK behavioural research: A review of national and international strategy documents. 2025 [cited 26 Sept 2025]. doi:10.7488/era/6399

16.	Shrout PE, Rodgers JL. Psychology, Science, and Knowledge Construction: Broadening Perspectives from the Replication Crisis. Annu Rev Psychol. 2018;69: 487–510. doi:10.1146/annurev-psych-122216-011845

17.	Bryan CJ, Tipton E, Yeager DS. Behavioural science is unlikely to change the world without a heterogeneity revolution. Nat Hum Behav. 2021;5: 980–989. doi:10.1038/s41562-021-01143-3

18.	Fernandes R, Job RFS, Hatfield J. A challenge to the assumed generalizability of prediction and countermeasure for risky driving: different factors predict different risky driving behaviors. J Safety Res. 2007;38: 59–70. doi:10.1016/j.jsr.2006.09.003

19.	Hagger MS, Chatzisarantis NLD, Barkoukis V, Wang JCK, Hein V, Pihu M, et al. Cross-cultural generalizability of the theory of planned behavior among young people in a physical activity context. J Sport Exerc Psychol. 2007;29: 2–20.

20.	Perry NS, Sullivan TJ, Leo K, Huebner DM, O’Leary KD, Baucom BRW. Using web-based technologies to increase reach, inclusion, and generalizability in behavioral observation research. J Fam Psychol JFP J Div Fam Psychol Am Psychol Assoc Div 43. 2021;35: 983–993. doi:10.1037/fam0000856

21.	Szaszi B, Goldstein DG, Soman D, Michie S. Generalizability of choice architecture interventions. Nat Rev Psychol. 2025;4: 518–529. doi:10.1038/s44159-025-00471-9

22.	BR-UK. Behavioural Research UK (BR-UK). In: Usher Institute [Internet]. 24 Sept 2024 [cited 28 Aug 2025]. Available: https://usher.ed.ac.uk/behavioural-research-uk

23.	Coupe N, Hart N, Yang G, Davan Wetton J, Fomina M, Webster R, et al. Building the UK’s Behavioural Research Capabilities: A scoping study and strategy from the Behavioural Research UK Leadership Hub. 2025 [cited 18 Dec 2025]. doi:10.7488/era/6036

24.	Behavioural Research UK. Behavioural Research Map. In: Usher Institute [Internet]. 24 Sept 2024 [cited 23 Oct 2025]. Available: https://usher.ed.ac.uk/behavioural-research-uk/our-research/br-uk-capability-scoping/br-uk-behavioural-research-map

25.	Coupe N, Davan Wetton J, Bauld L, Bordalo P, Byrne-Davis L, Cox S, et al. Understanding UK behavioural research capability in 2024: A cross-sector survey. SocArXiv; 2025. doi:10.31235/osf.io/vazwh_v1

26.	Sen A. Capability and Well‐Being. In: Nussbaum M, Sen A, editors. The Quality of Life. Oxford University Press; 1993. p. 0. doi:10.1093/0198287976.003.0003

27.	El Achi N, Honein-Abouhaidar G, Rizk A, Kobeissi E, Papamichail A, Meagher K, et al. Assessing the capacity for conflict and health research in Lebanon: a qualitative study. Confl Health. 2020;14: 59. doi:10.1186/s13031-020-00304-x

28.	Lecouturier J, Vlaev I, Chadwick P, Chater AM, Kelly MP, Goffe L, et al. The critical factors in producing high quality and policy-relevant research: insights from international behavioural science units. 2024 [cited 22 July 2025]. doi:10.1332/17442648Y2023D000000001

29.	Naru F. Behavioral public policy bodies: New developments & lessons. Behav Sci Policy. 2024;10: 1–17. doi:10.1177/23794607241285614

30.	Fels KM. Who nudges whom? Expert opinions on behavioural field experiments with public partners. Behav Public Policy. 2025;9: 212–248. doi:10.1017/bpp.2022.14

31.	Tazzini G, Gee B, Wilson J, Weber F, Brown A, Clarke T, et al. Overcoming challenges of embedding child and youth mental health research in community NHS services. J Public Ment Health. 2023;22: 275–286. doi:10.1108/JPMH-09-2023-0083

32.	Tinkler L, Robinson L. Clinical research nursing and factors influencing success: a qualitative study describing the interplay between individual and organisational leadership influences and their impact on the delivery of clinical research in healthcare. J Res Nurs JRN. 2020;25: 361–377. doi:10.1177/1744987120904778

33.	Puukko S, Heino MTJ, Kostamo K, Saurio K, Sniehotta FF, Hankonen N. How do behavioral public policy experts see the role of complex systems perspectives? An expert interview study. Transl Behav Med. 2024;14: 417–425. doi:10.1093/tbm/ibae024

34.	Silverstein P, Pennington CR, Branney P, O’Connor DB, Lawlor E, O’Brien E, et al. A registered report survey of open research practices in psychology departments in the UK and Ireland. Br J Psychol. 2024;115: 497–534. doi:10.1111/bjop.12700

35.	Hodgkinson GP, Whittington R, Johnson G, Schwarz M. The Role of Strategy Workshops in Strategy Development Processes: Formality, Communication, Co-ordination and Inclusion. Long Range Plann. 2006;39: 479–496. doi:10.1016/j.lrp.2006.07.003

36.	Nielsen MV, Bryndum N, Bedsted B. Organising stakeholder workshops in research and innovation – between theory and practice. J Deliberative Democr. 2017;13. doi:10.16997/jdd.285

37.	Fang ML, Grigorovich A, Wada M, Kontos P, Sixsmith J. Building Partnerships and Co-creating with Diverse Stakeholders. In: Sixsmith A, Sixsmith J, Mihailidis A, Fang ML, editors. Knowledge, Innovation, and Impact: A Guide for the Engaged Health Researcher: A Guide for the Engaged Health Researcher. Cham: Springer International Publishing; 2021. pp. 47–58. doi:10.1007/978-3-030-34390-3_6

38.	Marriott BR, Rodriguez AL, Landes SJ, Lewis CC, Comtois KA. A methodology for enhancing implementation science proposals: comparison of face-to-face versus virtual workshops. Implement Sci. 2016;11: 62. doi:10.1186/s13012-016-0429-z

39.	Becerra ZM, Fereydooni N, Kun AL, McKerral A, Riener A, Schartmüller C, et al. Interactive Workshops in a Pandemic: The Real Benefits of Virtual Spaces. IEEE Pervasive Comput. 2021;20: 35–39. doi:10.1109/MPRV.2020.3044072

40.	Zimmermann N, Pluchinotta I, Salvia G, Touchie M, Stopps H, Hamilton I, et al. Moving online: reflections from conducting system dynamics workshops in virtual settings. Syst Dyn Rev. 2021;37: 59–71. doi:10.1002/sdr.1667

41.	Egid B, Ozano K, Hegel G, Zimmerman E, López Y, Roura M, et al. Can everyone hear me? Reflections on the use of global online workshops for promoting inclusive knowledge generation. Qual Res QR. 2023;23: 195–216. doi:10.1177/14687941211019585

42.	O’Brien BC, Harris IB, Beckman TJ, Reed DA, Cook DA. Standards for Reporting Qualitative Research: A Synthesis of Recommendations. Acad Med. 2014;89: 1245. doi:10.1097/ACM.0000000000000388

43.	Davan Wetton J, Hart J, Bauld L, Bordalo P, Byrne-Davis LMT, Cox S, et al. Understanding UK behavioural research capability: A cross-sector survey. 2024. Available: https://doi.org/10.17605/OSF.IO/73BKF

44.	Fomina M, Webster R, Carey R. Building Behavioural Research Capabilities in UK Startups & Scaleups. Zinc VC; 2025. Available: https://www.zinc.vc/wp-content/uploads/2025/03/Zinc-Behavioural-Research-Report-2025.pdf

45.	Behavioural Research UK. Equality, Diversity, Inclusion and Intersectionality | Behavioural Research UK (BR-UK). In: Usher Institute [Internet]. 5 Mar 2025 [cited 9 Jan 2026]. Available: https://usher.ed.ac.uk/behavioural-research-uk-br-uk/about-us/principles-governance/equality-diversity-inclusion-and

46.	Chatham House. Chatham House Rule. n.d. Available: https://www.chathamhouse.org/about-us/chatham-house-rule

47.	Ritchie J, Spencer L. Qualitative Data Analysis for Applied Policy Research. In: Bryman A, Burgess B, editors. Analyzing Qualitative Data. London: Routledge; 1994. Available: http://dx.doi.org/10.4324/9780203413081

48.	Gale NK, Heath G, Cameron E, Rashid S, Redwood S. Using the framework method for the analysis of qualitative data in multi-disciplinary health research. BMC Med Res Methodol. 2013;13: 117. doi:10.1186/1471-2288-13-117

49.	Goldsmith LJ. Using Framework Analysis in Applied Qualitative Research. Qual Rep. 2021;26: 2061–2076. doi:10.46743/2160-3715/2021.5011

50.	Michie S, van Stralen MM, West R. The behaviour change wheel: A new method for characterising and designing behaviour change interventions. Implement Sci IS. 2011;6: 42. doi:10.1186/1748-5908-6-42

51.	Hofmann W, Betsch C, Böhm R, de Ridder D, Drews S, Ewert B, et al. Rethinking behaviour change interventions in policymaking. Nat Hum Behav. 2025; 1–3. doi:10.1038/s41562-025-02284-5

# **Supporting information**

S1 File. Standards for Reporting Qualitative Research (SRQR) checklist.

S2 File. Appendix A: Workshop procedure.

S3 File. Appendix B Framework tables.

S4 File. Appendix C: List of detailed strategies from each framework.
