# Supplementary material for: Developing the UK’s capability in behavioural research: Identifying strengths, challenges and strategies through cross-sector workshops
Source: PLoS One. 2026 Sep 10;21(9):e0357909. doi: 10.1371/journal.pone.0357909 (PMC13561315; doi:10.1371/journal.pone.0357909)
Supplement: Supplementary file 8 [file pone.0357909.s008.docx]

Dear Editor and Reviewers,

We are grateful for the review feedback. Below are our point-to-point responses.

Major comments:

| Reviewer comment | Changes (new text underlined) |
| --- | --- |
| 1. Methodological rigour and transparency need strengthening  The manuscript is generally well structured and addresses an important topic. However,the methods section should provide more detail on the analytical process, particularly how the initial codes were generated, how coding consistency was ensured across researchers, how disagreements were resolved, and how the 141 codes were grouped into the eight final frameworks. A coding tree or a table showing the correspondence between codes, subthemes, themes, and final frameworks should be provided, preferably as a supplementary file. | Thank you for this feedback. We have expanded the Data analysis section to describe how the initial codes were generated, how coding consistency was ensured, and how disagreements were resolved. In the Familiarisation and initial coding subsection, the text now reads: "The core analysis team of three researchers (KS, GY, NH) familiarised themselves with the data. The analytical approach was piloted with a subset of notes from three selected breakout rooms, which the three researchers first coded independently before comparing and calibrating their coding with a fourth researcher (NC). Codes agreed in this pilot phase formed a shared initial coding frame; disagreements were resolved through consensus discussion, and consistency was maintained through frequent team meetings throughout the analysis.”  In the Charting subsection, the text now read: “The 141 codes were grouped into 24 umbrella themes based on conceptual similarity and then were further organised into eight separate framework matrices based on thematic coherence”. And we also added “The full coding tree, showing the correspondence between the 141 codes, the key themes, and the eight final frameworks, is provided in S5 File (Appendix D).” |
| 2. Need to discuss potential biases related to the qualitative workshop methodology  The study uses online workshops as the main qualitative data collection method. but this approach is vulnerable to several biases that should be explicitly discussed.  The limitations section should include a more explicit discussion of the potential biases associated with qualitative workshop methods, including selection bias, self-selection bias, social desirability bias, group conformity, and dominance effects within breakout rooms. The authors should also explain what measures were taken to mitigate these risks, such as facilitation techniques, inclusive participation strategies, use of chat functions, anonymisation, validation of findings, or triangulation with transcripts. | Thank you for this feedback. We have addressed these comments across the manuscript.  Firstly, in our Strengths and limitations subsection we amended the text to read:  “Workshops as a data collection method can create challenges through group dynamics, including dominant voices, underrepresentation of dissenting views, social desirability and conformity pressures. This was addressed through i) the use of experienced and well-briefed facilitators, ii) agreeing at the outset that contributions would be treated as confidential to the workshop and not attributed to individuals or organisations, and iii) encouraging use of the chat function, and iv) in the analysis process, triangulating topics of discussion across transcripts ... There are a few key limitations of this study. There was a risk of selection and self-selection sampling bias as those already invested in increasing behavioural research capability may have agreed to participate, and the primary pool for invitees consisted of those who had already engaged with BR-UK in their initial survey, making them a particularly research-engaged group.”  We have suggested in the Implications for research subsection that future research can expand on our findings:  “As a study that builds directly on BR-UK’s UK-wide capability survey [25], this workshop study adds qualitative depth to previously identified quantitative patterns. In-depth individual interviews could further enrich specific findings without the social complexities of a group workshop.” |
| 3. Clarify how strategies were generated and analysed  The study first identifies strengths and barriers, then presents ten strategies to strengthen behavioural research capability. However, the methodological pathway from identifying strengths and barriers to generating strategies is not sufficiently clear.  The methods section states that participants discussed strategies during the workshops and that the authors later mapped actor groups with potential responsibility for change. However, the process by which the ten strategy areas were derived, structured, prioritised, or linked to the eight frameworks is not described in enough detail. The results section presents ten overarching strategies, but the analytical logic behind this step could be more systematic. The authors should describe how the ten overarching strategies were derived, how they correspond to the eight frameworks, and whether any prioritisation or validation process was used. | To address this, we have added the details to the Interpretation subsection (Methods). The new text reads:  “Although strategy was the focus of the final breakout session of each workshop, where participants were directly asked to explore strategies or solutions that would improve or strengthen behavioural research in their organisation, strategies and solutions were spontaneously discussed throughout the workshop sessions. Strategy data were mapped onto the same thematic frameworks as the strengths and challenges data, so that each framework contained its own set of participant-proposed strategies. From each framework, the research team selected the most salient strategies through team discussion; selection was guided by the level of discussion, agreement and support in workshops, and corroboration from the strengths and challenges data. For reporting, the selected strategies were then consolidated into 10 strategies by combining those that recurred across multiple frameworks. Each of the 10 strategies comprises a set of concrete operational actions proposed by participants. Each strategy was mapped back onto the framework(s) from which it originated and onto the actor groups, that is, the individuals and organisations with potential agency and responsibility to implement it (Table 3).  Workshop participants were invited to review and validate the initial framework draft in July 2025 to ensure accuracy of the interpretation. The consolidated strategy table, developed later, was shared with the work package group for expert input and confirmation. |
| **4. Results section could be more clearly separated from interpretation and discussion**  The results section is rich, but in several places it appears to move beyond description and begins to interpret, explain, or contextualise the findings. In a qualitative paper, some interpretation is expected, but the distinction between **findings**, **interpretation**, and **discussion with literature** should remain clear.  The results section should be reviewed to ensure that it remains primarily focused on reporting findings from the workshops. Explanatory interpretation, comparison with previous studies, and broader implications should be moved to the discussion section where appropriate. The use of references in the results section should also be reconsidered, unless they are necessary to define specific concepts mentioned by participants. | Thank you for this comment. We have reviewed the full Results section (Frameworks 1–8 and the Strategies subsection) to ensure it remains focused on reporting the findings from the workshops, and have made the following changes:  1) Interpretive and forward-looking content moved to the Discussion. The reflection on how the successful application of behavioural insights during the COVID-19 pandemic raises questions about embedding similar approaches in future crisis responses has been moved from the Results (Framework 5) to the Discussion.  2) Participants’ reasoning is now explicitly attributed. Where sentences could previously be read as authorial interpretation, they now report participants’ own inference. For example, “…a pervasive belief that everyone inherently understands human behaviour, which they felt led to resistance to evidence-based approaches." (Framework 1); “…which participants said made it harder to attract quality researchers” (Framework 7); "…with participants calling for succinct summaries" (Framework 2).  3) References in the Results reconsidered. Following your suggestion, we reviewed all citations in the Results: the only reference retained is COM-B [50], which participants named directly in the workshops and which requires a defining citation, is consistent with the exception you described. |
| **5. Tables require improvement in readability and presentation**  The tables contain useful information, but their current presentation is dense and difficult to read. Table 1 in particular contains many bullet points, sector abbreviations, and long text entries, which may reduce readability. | Thank you for this feedback. We have restructured both tables to improve readability. Table 1 has been split into two tables, Table 1 (Strengths) and Table 2 (Barriers), keeping one row per finding, with the wording of each entry shortened. Sector abbreviations have been removed: the Workshops column now gives the full workshop names, with “All workshops” used where a finding was identified across all five. A note beneath each table explains the column, and the complete item-level frameworks remain available in S3 File (Appendix B).  The strategies table (renumbered Table 3 following the split) has been streamlined: each strategy now carries a single illustrative quote. We also shortened the key actors list by moving recurring definitions moved to the table footnote. |

Reviewer comments supplied in word document

| **Original manuscript text (reviewer’s anchor highlighted)** | **Reviewer’s comment** | **Response and changes made (new text underlined)** |
| --- | --- | --- |
| **Much of the theoretical contributions of behavioural research pertains to processes of change**; yet there is a need for more theoretical explanation of variations in behavioural outcomes and of the generalisability of behavioural research findings [18–21]. | What do you mean by processes of change? define it | On reflection, the authors have agreed this line is not required in the manuscript. It has now been removed. |
| **How these various challenges differ across sectors and disciplines is not clear**. | To be reformulated | Thank you for highlighting this. On reflection, the authors agree this line is not required in the manuscript. It has now been removed. |
| We currently lack a comprehensive picture of how well-equipped UK-based **organisations** are to conduct high-quality behavioural research to meet identified needs in policy and practice, and translate it into impactful outcomes. | Could you please clarify what you mean by “organisation”? Does this refer to governmental bodies, non-governmental organisations, research institutions, or administrative entities? | Thank you for the suggestion. We have now clarified with the following text (Paragraph 2, Introduction): “We currently lack a comprehensive picture of how well-equipped UK-based organisations (including academic institutions, governmental departments and teams, and private, public and third sector companies or teams) are to conduct high-quality behavioural research to meet identified needs in policy and practice...” |
| Building on a documentary review of national and international strategy documents for advancing behavioural research capability [15], organisational network mapping [24] and a UK-wide survey [25], **these workshops** provided essential qualitative insights into sector-specific challenges and opportunities for strengthening behavioural research capability. | Whose workshops are these, and in what context were they conducted? To be reformulated: Something is missing to ensure proper understanding | Thank you for this suggestion. We have clarified with the following amended text (Paragraph 3, Introduction):  “Building on a documentary review of national and international strategy documents for advancing behavioural research capability [15], organisational network mapping [24] and a UK-wide survey [25], the workshops reported in this study represent the next phase in BR-UK’s capability building programme of work and provided essential qualitative insights into sector-specific challenges and opportunities for strengthening behavioural research capability.” |
| While these studies have documented various progress and capability challenges such as funding constraints [28,29] and translation difficulties [31], most focused on specific **sectors or contexts**. | Give examples | Thank you for the suggestion. We have explained further what these sectors and contexts are referring to (p.4, Paragraph 4, Introduction): “Most studies have concentrated on single sectors or contexts, such as behavioural science units advising policymakers [28,29], health services [31], or academic psychology departments [32]” |
| By mapping strengths, barriers and strategies for improvement across sectors, we aim to **provide these stakeholders with evidence for strategic decision-making and coordinated action**. | To be reformulated : for what? | Thank you for this suggestion. We have clarified with the following amended text (p. 5, Paragraph 5, Introduction):  “By mapping strengths, barriers and strategies for improvement across sectors, we aim to provide these stakeholders with 1) evidence for strategic decision-making and organisational improvement through outlining barriers and enablers and ii) actionable, participant-generated strategies for strengthening their production and use of behaviour research, and for coordinating behavioural research across and within teams, organisations and sectors.” |
| What are the current strengths of existing behavioural research, including its impact on policy and practice in **each sector**? | The sectors are not clearly identified. The choice of sectors needs to be justified, and the research objectives should be stated more precisely | Thank you – we have now clarified these points with the following amended text (p. 5, Paragraph 5, Introduction):  “ Based on the findings from BR-UK's previous capability building research phases and gaps identified in the wider literature, this study used inputs from researchers, organisational leaders, funders, and training bodies across academia, government, private, public and third sector bodies seeking to strengthen behavioural research capability across the UK. By mapping strengths, barriers and strategies for improvement across the sectors outlined above, we aim to provide these stakeholders with i) evidence for strategic decision-making and organisational improvement through outlining barriers and enablers and ii) actionable, participant-generated strategies for strengthening their production and use of behaviour research, and for coordinating behavioural research across and within teams, organisations and sectors.” |
| What are the barriers to and missed opportunities in conducting and using behavioural research in **each sector**? | Same comment | Each research question now includes the sectors in full for clarity (p. 5-6, Introduction):  “...in academia, government, private, public and third sector organisations?” |
| Workshop procedure and **data collection** | Place this in a separate section | Thank you for this suggestion. We have now separated the text into two sections: Workshop procedure, and Data collection. |
| **The workshops identified a range of strengths and barriers to building behavioural research capability across the UK. The eight frameworks developed through the analysis process centre around eight core themes are:** (1) public perceptions and understanding, (2) communication and translation, (3) collaboration, networking and engagement, (4) research practice and methodology, (5) behavioural research impact, (6) funding, resources, capacity and accessibility, (7) training and career development, and (8) leadership, embedding and prioritisation. | This sentence lacks grammatical clarity and fluency in English and would benefit from reformulation | We have reworded this paragraph for clarity with the following amended text:  “Participants identified a range of strengths and barriers to building behavioural research capability across academic, government, private, public and third sectors across the UK. These eight core themes were derived through the analysis: (1) public perceptions and understanding, (2) communication and translation, (3) collaboration, networking and engagement, (4) research practice and methodology, (5) behavioural research impact, (6) funding, resources, capacity and accessibility, (7) training and career development, and (8) leadership, embedding and prioritisation. Each is represented in a separate framework table for clarity, although some frameworks are connected and overlapping in nature. |
| **A, G, C** [sector-code cell in Table 1] | Do not use abbreviations | Thank you – the abbreviations have now been spelled out in Tables 1 and 2. |
| Framework 2: **Communication and translation** | These are two different competencies: communication relates to interactions and interpersonal action, whereas translation refers to the transformation and transfer of theory into actionGroup similar and related concepts together, and revise this theme/framework | Thank you for this comment, which we have considered carefully. We agree that communication and translation each mark distinct processes, actions and skills. On reviewing the workshop data itself, we have concluded that the overlap and complementarity of the concepts is too great to meaningfully pull the two apart in the report. However, to address the important point you raise, we have added text to clarify this decision (p. 20, Framework 2, Results): “While we recognise that communication and translation are distinct concepts, workshop participants discussed and explored them as intrinsically related concepts and therefore they have been analysed and reported together.” |
| Across the workshops, participants **suggested and discussed strategies by which behavioural research capability could be improved in their sector and/or area of expertise**. | It would be helpful to explain how the participants developed these SWOT-based strategies. Were they proposed through a structured discussion process? It would also be important to clarify to what extent the proposed strategies are considered effective and feasible, and whether they are strategic directions or concrete operational actions and activities???? | Thank you for these suggestions. To address how the strategies were developed, we have added the following line:  “Across the workshops, participants suggested and discussed strategies by which behavioural research capability could be improved in their sector and/or area of expertise. Strategy ideation was the focus of the final third breakout session of each workshop, but participants also raised and discussed strategies spontaneously throughout the workshops.”  To address your second point about feasibility and effectiveness of proposed strategies- we did not ask participants to explore feasibility or effectiveness of proposed strategies. We have outlined in the discussion where these strategies address known and pervasive gaps, and where they differ from other strategies. We also acknowledged this as a limitation (see our response below). |
| The strategies centre **around ten overarching approaches** to improving capability: 1) Creating accessible communication resources, 2) Establishing a central repository/resource bank, 3) Embedding behavioural science into policy and research processes, 4) Building multi-level training programmes, 5) Reforming funding structures, 6) Establishing behavioural research leadership and champions, 7) Strengthening stakeholder and public engagement, 8) Creating transdisciplinary roles and networks, 9) Shifting to systems-level approaches, and 10) Establishing quality standards and accessible guidance. | Ten strategies seem too many. It would be more effective to group them into a smaller number of broader strategic areas, for example four or five, and then present concrete operational actions under each strategy. This would make the framework clearer, more coherent, and easier to implement. Please also consider the previous comment regarding the distinction between strategies and concrete actions | Thank you for this suggestion, which we considered carefully, including drafting a version grouped into five broader strategic areas. On reflection, we concluded that the ten strategies as presented best preserve fidelity to the workshop data: each strategy was derived directly from participants’ discussions through the framework analysis described in the Analysis and reporting of strategies subsection (Methods), and the full set was validated with the internal work package team. Rather than introducing higher-level groupings that participants did not themselves generate, we have instead clarified the structure this comment raises: each of the ten strategies is a strategic direction, and the concrete operational actions beneath each one (presented in Table 3) describe how it can be implemented. We have added the following sentence to the Results: “Each strategy comprises a set of concrete operational actions proposed by participants; the strategies are thematic and are not presented in ranked order.” We have also substantially streamlined Table 3 to improve its clarity and coherence, using one illustrative quote per strategy, numbered framework correspondences, and condensed actor lists, with full detail in S4 File. |
| Strengths and **limitations** | Several additional methodological limitations could be further acknowledged. First, the study provides limited information on the composition and representativeness of participants across sectors. Second, workshops may be affected by group dynamics, including dominance of some voices, consensus-seeking, and underrepresentation of dissenting views. Third, the reliance on workshops limits the depth of individual perspectives, and complementary interviews could have provided richer insights. Fourth, it is not clear whether the proposed strategies were assessed in terms of feasibility, acceptability, priority, cost, or potential impact. Finally, the distinction between broad strategies and concrete operational actions should be clarified, as this affects the practical usefulness of the findings | Thank you – we have addressed your feedback across the manuscript. Under Strengths and limitations (Discussion), we amended the text:  “Workshops as a data collection method can create challenges through group dynamics, including dominant voices, underrepresentation of dissenting views, social desirability and conformity pressures. This was addressed through i) the use of experienced and well-briefed facilitators, ii) agreeing at the outset that contributions would be treated as confidential to the workshop and not attributed to individuals or organisations, and iii) encouraging use of the chat function, and iv) in the analysis process, triangulating topics of discussion across transcripts. … There are a few key limitations of this study. There was a risk of selection and self-selection sampling bias ...We did not collect detailed information on the composition and representativeness of participants across sectors, making us unable to comment on the generalisability of our findings across sectors. Furthermore, the proposed strategies were not implemented or evaluated in this study, resulting in limited information regarding feasibility, acceptability, priority, cost, or potential impact.”  In Implications for research subsection (p. 43), we added the following line:  “In-depth individual interviews could further enrich specific findings without the social complexities of a group workshop.”  We have addressed the final point on broad strategies versus actions in Methods and Results sections, as described in our responses above. For example, in Results, we clarified that: “. Each strategy comprises a set of concrete operational actions proposed by participants.” |
| This study has gathered and thematically organised key, **actionable** strategies suggested by behavioural researchers and behavioural research users from across sectors and topic areas. | For whom, in which context, and how? What evidence supports their “actionable” nature? Were they tested or validated? | In order to avoid ambiguity around, or overstating, the word ‘actionable’, we have reworded the text in the Implications for policy and practice subsection (Discussion), which now reads:  “Many of the strategies to improve behavioural research capability provide practical next steps for those wanting to support behavioural research and its use in their organisation in the UK. For example, government departments could benefit from making behavioural research roles in their civil services more visible and create more opportunities for secondments for behavioural researchers into government, to facilitate better research-policy translation... More strategically, organisational leaders across sectors could considering embedding behavioural research into organisational decision-making processes and establishing formal champion roles to address the leadership gap, while research funders could usefully commit to increased, long-term funding for cross-sector multi-disciplinary collaboration. This would address the critical funding barriers identified across all sectors by establishing longer funding cycles and creating funder collectives for large-scale initiatives.” |
